# Building-related health impacts in European and Chinese cities: a scalable assessment method

**DOI:** 10.1186/s12940-015-0082-z

**Published:** 2015-12-14

**Authors:** Jouni T. Tuomisto, Marjo Niittynen, Erkki Pärjälä, Arja Asikainen, Laura Perez, Stephan Trüeb, Matti Jantunen, Nino Künzli, Clive E. Sabel

**Affiliations:** Department of Environmental Health, National Institute for Health and Welfare, P.O.Box 95, FI-70701 Kuopio, Finland; Environmental protection services, P.O. Box 228, 70101 City of Kuopio, Finland; Swiss Tropical and Public Health Institute, Socinstrasse 57, 4002 Basel, Switzerland; University of Basel, Petersplatz 1, 4003 Basel, Switzerland; Department of Air Hygiene of Basel City and Basel County, Rheinstrasse 44, 4410 Liestal, Switzerland; School of Geographical Sciences, University of Bristol, University Road, Bristol, BS8 1SS UK

**Keywords:** Public health, Climate change, Building stock, Cities, Policy support, Health impact assessment, Heating, Fine particles, Energy production

## Abstract

**Background:**

Public health is often affected by societal decisions that are not primarily about health. Climate change mitigation requires intensive actions to minimise greenhouse gas emissions in the future. Many of these actions take place in cities due to their traffic, buildings, and energy consumption. Active climate mitigation policies will also, aside of their long term global impacts, have short term local impacts, both positive and negative, on public health.

Our main objective was to develop a generic open impact model to estimate health impacts of emissions due to heat and power consumption of buildings. In addition, the model should be usable for policy comparisons by non-health experts on city level with city-specific data, it should give guidance on the particular climate mitigation questions but at the same time increase understanding on the related health impacts and the model should follow the building stock in time, make comparisons between scenarios, propagate uncertainties, and scale to different levels of detail.

We tested The functionalities of the model in two case cities, namely Kuopio and Basel. We estimated the health and climate impacts of two actual policies planned or implemented in the cities. The assessed policies were replacement of peat with wood chips in co-generation of district heat and power, and improved energy efficiency of buildings achieved by renovations.

**Results:**

Health impacts were not large in the two cities, but also clear differences in implementation and predictability between the two tested policies were seen. Renovation policies can improve the energy efficiency of buildings and reduce greenhouse gas emissions significantly, but this requires systematic policy sustained for decades. In contrast, fuel changes in large district heating facilities may have rapid and large impacts on emissions. However, the life cycle impacts of different fuels is somewhat an open question.

**Conclusions:**

In conclusion, we were able to develop a practical model for city-level assessments promoting evidence-based policy in general and health aspects in particular. Although all data and code is freely available, implementation of the current model version in a new city requires some modelling skills.

**Electronic supplementary material:**

The online version of this article (doi:10.1186/s12940-015-0082-z) contains supplementary material, which is available to authorized users.

## Background

Public health is often affected by societal decisions that are not primarily about health. Even if health is considered a high priority, decision processes are not well equipped to systematically and quantitatively deal with health, if the primary focus of a decision is about, e.g., economy, construction of buildings, generation of energy, or traffic.

This is especially true for overarching policy issues such as climate change, which is the major environmental concern of our time. Then, decision processes aim to handle the primary focus and take account of also climate as an important secondary issue. This is already challenging, and tertiary issues such as public health in this case are rarely considered systematically. There is a clear and urgent need for tools that help quantification of public health impacts in such situations.

Cities contribute significantly to the overall greenhouse gas (GHG) emissions as most of the traffic, industry, commerce and more than 50 % of world's population are located in urban areas. Therefore, active climate mitigation policies are needed and are increasingly being implemented on city level [1–3].

Climate mitigation is more effective and cost-efficient, if it is implemented as a part of the normal development, maintenance, and other activities. Therefore, decision makers and authorities on city level need information about climate and health issues and tools to reflect that information in their decision making processes. Similarly, there is a need to assess climate adaptation decisions on city level, but that is beyond the scope of this article.

EU FP7 funded project URGENCHE studied the health impacts of climate policies in two cities in China (Suzhou and Xi’an) and 5 cities in Europe (Basel, Kuopio, Rotterdam, Stuttgart, and Thessaloniki). The main study areas included heat and power generation, traffic, buildings and their effect on health and well-being. The assessed climate policies for each city were defined and formulated based on the actual climate strategies of the cities.

The seven cities participating to the URGENCHE project varied vastly in their size, geographical location, climate conditions, income etc. Only Kuopio, Basel and Thessaloniki assessed climate policies targeted on buildings, but also Rotterdam and Suzhou indicated that some of their GHG mitigation policies are related to energy efficiency and sources of heat for the buildings. Rotterdam relies on the Dutch national program for insulating buildings and a large proportion of the buildings are switching from natural gas to waste heat from industrial sources and in Suzhou all new buildings in year 2050 should be "green", ie. heated by renewable energy. The proportion of energy used in buildings varies between cites from dominant (Kuopio 35 % of the total energy use) to marginal (5 % in the heavily industrial harbour city of Rotterdam, and even less in Suzhou), thus minimising the relative GHG and ambient air quality potentials of building related policies in some cities. The absolute impact, however, can be equally significant in both ends of the range.

Rotterdam has an exceptional level of energy intensive industry, and the role of buildings in the total energy consumption is only 5 %, similar to the two participating Chinese cities. In Suzhou and Xi’an the total energy demand and CO_2_ emissions are still rapidly increasing. The building stocks of the two cities, residential in particular, consume only marginal proportions of the total energy, which is dominated by industry. Respectively the energy conservation and GHG mitigation potentials of building policies are small. In Suzhou – one of the most advanced, modern and affluent cities of China – the role of buildings appears to be marginal also for local air pollution. In Xi’an, on the other hand, widespread small scale combustion of coke briquettes and coal for residential heating and cooking and the needs of various small businesses appear to be a key factor in the very high ambient air pollution levels, and, thus, cause of public health risks.

The building stock does not only consume energy, it also most significantly influences health and comfort of the population. In OECD countries 20 % of total energy was consumed in residential buildings in 2013 [4]. Key determinants for health and comfort in buildings are air quality, dampness, draught and indoor temperature, which are linked to energy efficiency via heating, ventilation, and insulation. Many climate policies aim at reducing the energy consumption of buildings and therefore influence health and comfort of the people. These influences can be positive and/or negative. Positive effects are usually mediated via better indoor air quality and decreased dampness through improvements in ventilation and increased thermal comfort through better insulation [5].

However, increased insulation may also cause negative effects via reduced air exchange, increased indoor temperature in the summer and increased moisture accumulation in the building structures. The latter applies especially in the cold climate zones: energy conservation regulations can only be met by thick insulation layer, where leaking indoor air may reach dew point and condense water. Where current insulation is inadequate, however, properly installed additional insulation is an investment which both saves money and improves health and comfort. Monetary savings by insulation can also have equity benefits as they are relevant for subgroups that have difficulties to afford cost of heating.

The critical question of building-related policies is how to include the secondary and tertiary aspects to decision making, i.e. how to reduce GHG emissions and promote health at the same time when managing the building stock of a city. The aim of this study was 1) to develop a modelling tool for the assessments of building stock impacts on various health parameters and GHG emissions, and 2) to demonstrate the applicability of this tool in the participating URGENCHE cities for dynamic (past and future) projections. The model should follow the building stock in time, make comparisons between scenarios, propagate uncertainties, and scale to different levels of detail. We demonstrate the building model with two policies in Kuopio and in Basel providing estimates of changes in the building stock i.e. changes in floor area of built space and in heating energy demand, GHG and PM_2.5_ emissions as well as health effects up to 2050. However, this model does not consider the health impacts of exposure to insulating materials or materials toxicity.

There is another modelling tool for similar city-level energy and climate assessments [6]. However, this tool is owned by a private company and it is used in consultancy projects. Therefore, there is a clear need for tools that are free and openly available for any interested city.

**Fig. 1:**
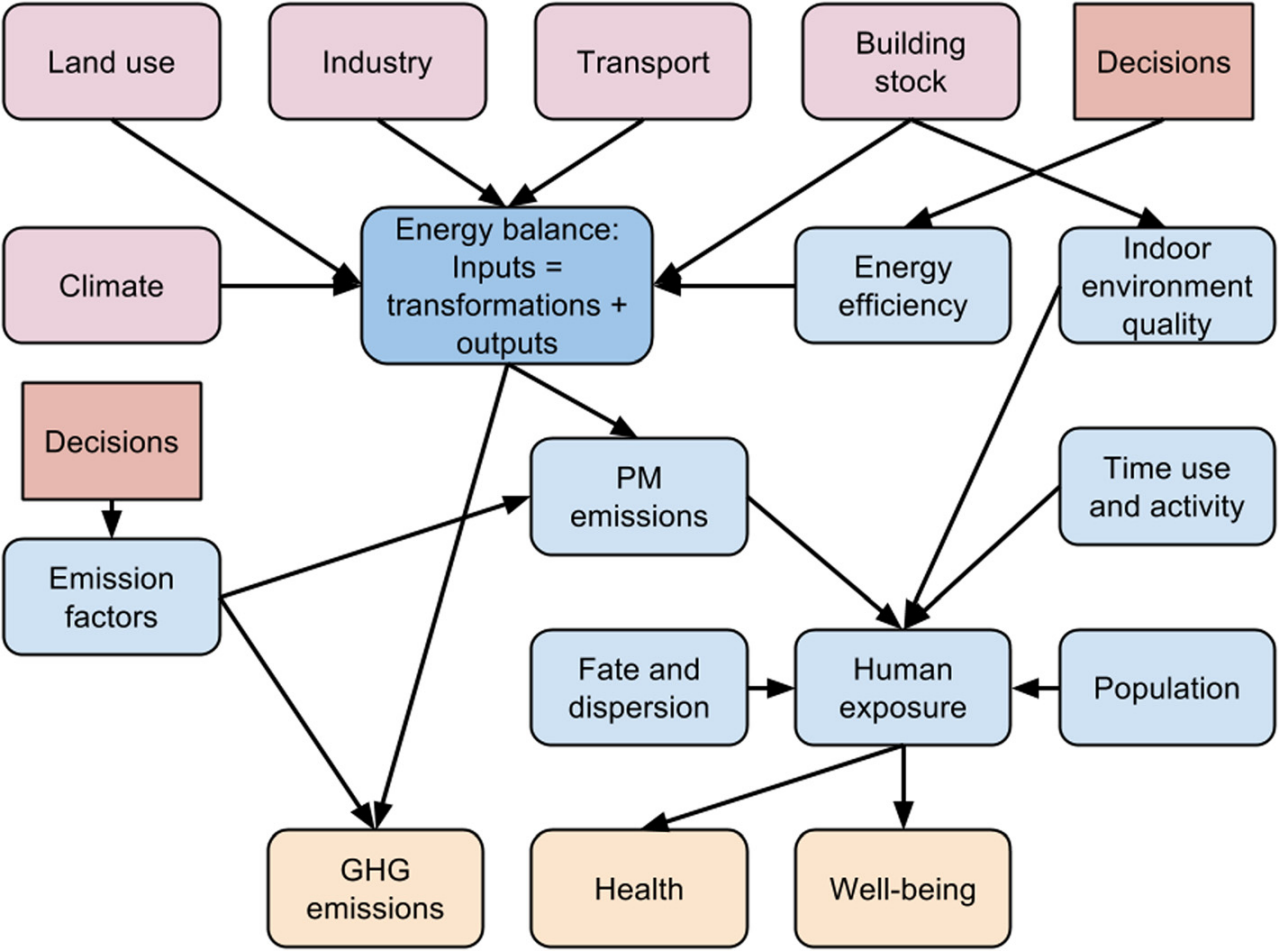
Conceptual model of important factors related to city-level energy balance and buildings. Driving forces (pink) and outcomes of interest (orange)

The conceptual placement of buildings in relation to energy balance of a city and health of population is shown in Fig. 1. In this article, we only concern the nodes that relate to the node *Building stock*. Other driving forces (i.e. climate, land use, industry, and transport; in pink) are discussed in other URGENCHE articles.

**Fig. 2:**
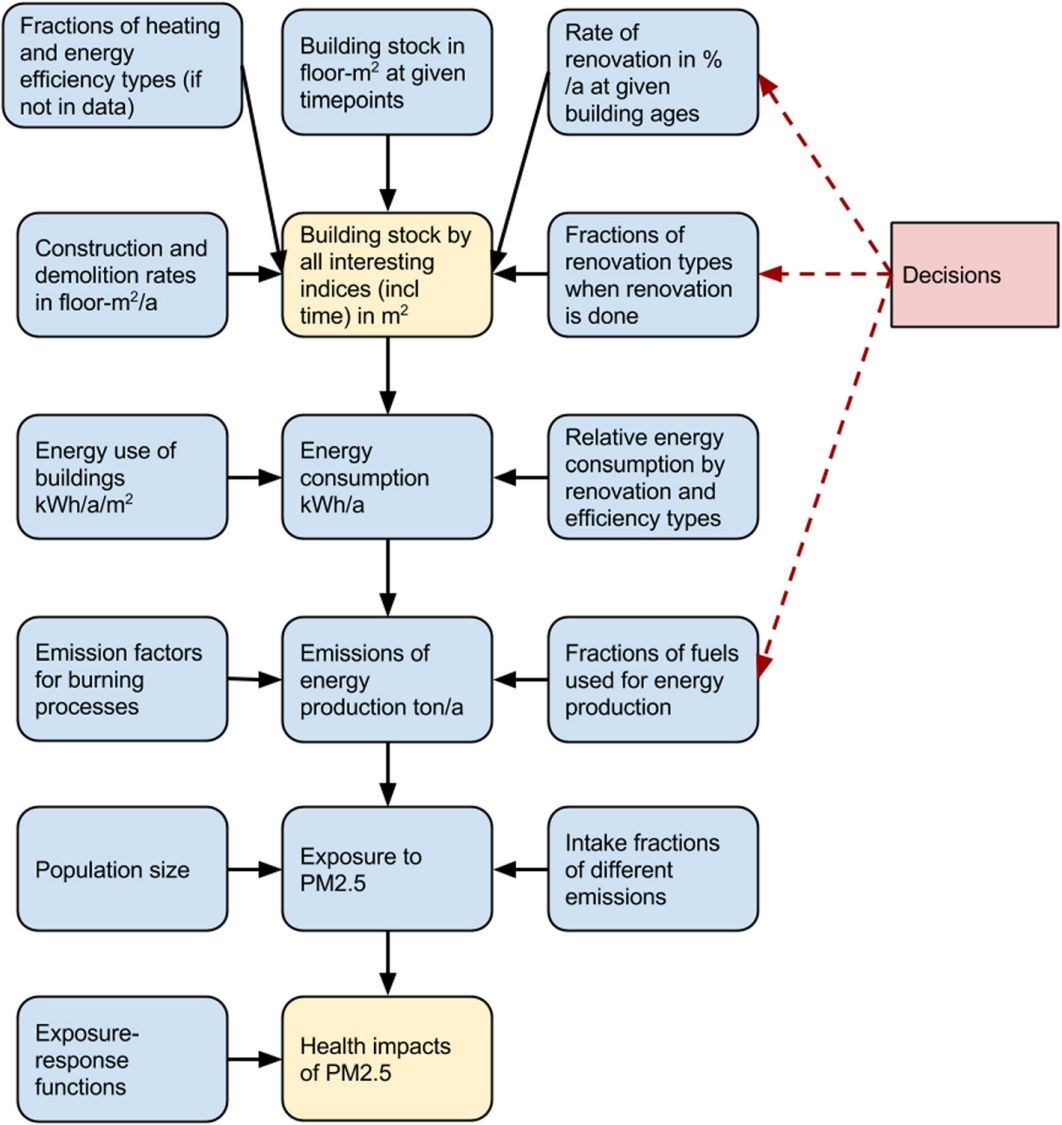
The actual modules of the computational model

## Methods

### Description of the study cities

The following cities participated in URGENCHE project: Basel (Switzerland), Kuopio (Finland), Rotterdam (Netherlands), Stuttgart (Germany), Thessaloniki (Greece), Suzhou (China), and Xi'an (China). Some basic statistics for each study city are given in Table 1.

**Table 1:** Basic description of URGENCHE cities

City	Population^a^	Population density / km^2^ ^a^	Area (km^2^)	Annual mean temperature^b^	Total annual precipitation^b^	GHG emissions Mt CO_2_-eq	Life expectancy male/female
Basel	192 000	7 564	23.9	9.5	784	2.4	76.1 / 81.6
Kuopio	105 000	46	3165.0	2.7	498	1.02	76.7 / 83.2^d^
Rotterdam	550 000	2 952	325.8	10.4	856	32.6	75.7 / 81.2
Stuttgart	590 000	2 958	207.4	9.6	689	5.1	78 / 83^e^
Suzhou	10.6 million (urban 5.5 million)	1 200 (urban 2 000)	8 488 (urban 2 743)	17.0	932	181	74 / 77^f^
Thessaloniki	1.1 million (urban 790 000)	692 (urban 7 080)	1 456 (urban 112)	15.6	458		78 / 84^g^
Xi’an	8.5 million (urban 6.5 million)	850 (urban 7 900)	9 983 (urban 826)	average high 19.3, average low 9.2	553	road traffic 15^c^	73.3 / 78.3

^a^Wikipedia, ^b^Basel, Kuopio, Stuttgart, Thessaloniki: climatemps.com; Rotterdam, Suzhou, Xi'an: Wikipedia, ^c^total not available, ^d^Terveyskirjasto (www.terveyskirjasto.fi), ^e^WHO 2011 Germany (www.who.int/gho/database/en), ^f^WHO 2011 China, ^g^WHO 2011 Greece

### Kuopio

**Fig. 3:**
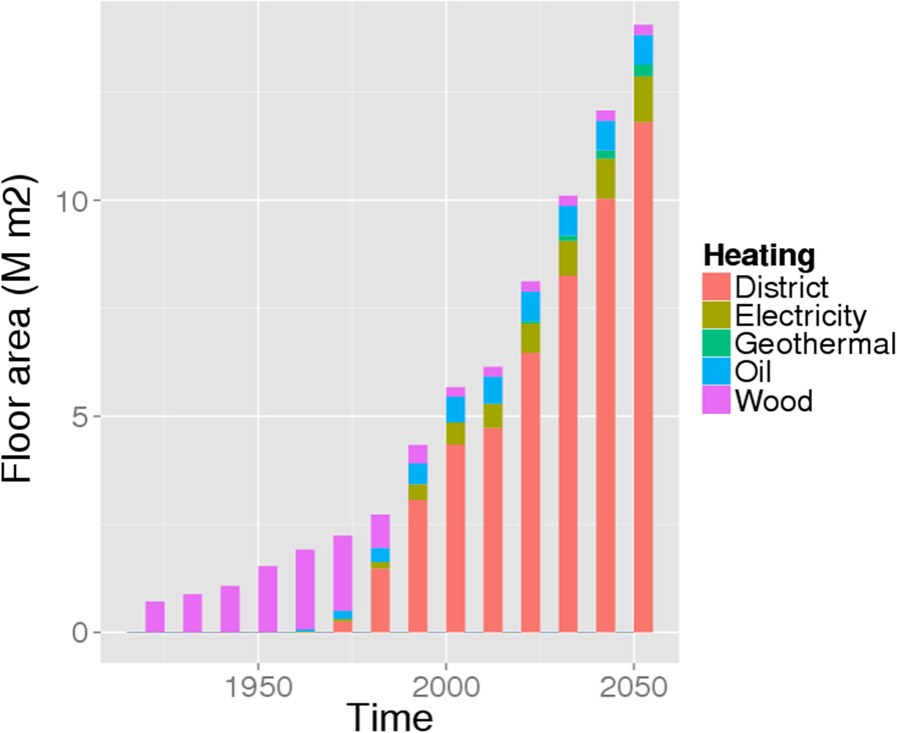
Building stock in Kuopio by heating type

Kuopio is the 8th largest city in Finland. It is located in eastern Finland (coordinates 62°53'33"N 027°40'42"E). In addition to the compact urban area, where 85 % of the population lives, the municipality includes large rural and lake areas. The building stock of Kuopio is heated mainly via a district heating network (88 %), by fuel oil (8 %) and electricity (4 %) (Fig. 3). In most detached homes wood is used for supplementary heating. In total there are 50 000 buildings and 60 000 dwellings in the whole Kuopio. Heating of buildings causes 43 % of the total GHG emissions (district heating 35 %, electric heating 4 %, separate heating 4 %).

### Basel

Basel is the third largest city of Switzerland. It is located in the corner where Switzerland, France and Germany meet (coordinates 47°34'N 7°36'E). Combustion makes up 56 %, road traffic 26 %, and waste management 18 % out of the total annual GHG emissions. 100 % of electricity used in Basel comes from renewable sources. Residential heating and hot water accounts for 22 % of the total energy use in Basel.

### Assessed policies

#### Climate policy of Kuopio 2009–2020

Key components of the climate policy of Kuopio are on one hand heat and power cogeneration, which provides 88 % of all space heating and 61 % of all electric power used within the municipality, and on the other hand replacement of the (semi)fossil peat with renewable biomass (Table 2). District heat for the building stock within the 440 km heat distribution network is supplied from the Haapaniemi 2 and 3 cogenerating stations with respective capacities of 120 MW heat and 60 MW power, and 80 MW heat and 49 MW power. Both apply fluidised bed combustion technologies, which enable flexible use of biofuels, SO_2_ removal and low NO_X_ emissions. The proportion of biofuels has increased from 4 % in 2009 to over 50 % in 2014, and, depending on the supply, will continue to increase. Oil fired boilers provide reserve and peak capacity, but generate on average only 2 % of the annual heat demand. The objective of Kuopio's climate policy is to reduce the fossil GHG emissions from the 1990 to 2020 by 40 %. Peat is classified as fossil fuel, with its CO_2_ emission factor of 380 kg/MWh, whereas the combustion of wood is assumed to be greenhouse neutral, i.e., its fossil CO_2_ emission factor is 0, although its direct/immediate CO_2_ emission factor is 420 kg/MWh (Additional file 1: Table S1–6).

**Table 2:** Studied climate policies of Kuopio and Basel and business as usual (BAU) scenarios

City	Renovation BAU	Active renovation	Efficient / Total renovation	Fuel BAU	Fuel policy
Kuopio	3 % of >30-year-old buildings renovated per year	4.5 % of >30-year-old buildings energy-renovated per year	BAU + sheath reform to all renovated buildings	84 % peat, 12 % heavy oil and 4 % biomass in Haapaniemi plant	49 % peat, 50 % wood biomass and 1 % heavy oil in Haapaniemi plant
Basel	1 % of >30-year-old residential buildings renovated per year	2 % of >30-year-old buildings renovated per year	All >30-year-old buildings renovated	50 % waste, 10 % wood, and 40 % gas in district heating	50 % waste, 30 % wood, and 20 % gas in district heating

Other measures of Kuopio's climate policy include reducing the energy used in public buildings by 9 % from 2005 to 2016 and conservation campaigns for the public. In addition the national building codes provide energy conservation incentives and regulations for all new and renovated buildings. In general, the GHG mitigation policies of Kuopio focus mostly on the public utilities and other public measures.

### Climate policy of Basel

The energy policies implemented during these past decades in Basel have resulted in CO_2_ emissions lower than the annual 2.4 Mt target of the Federal government. The city of Basel energy policy now revolves around reducing individual consumption and shifting towards renewable energy. The current policy relies on 4 pillars:

- Currently 100 % of the electricity used in Basel city originates from renewable sources, 90 % hydro, the rest from biomass, solar and wind. In 2010 gas and fuel oil provided almost half of the space heating for Basel. District heat provides the other half by a 25 MWt wood fired cogenerating station, a connected geothermal plant and a 17.2 MWt waste incineration plant. In addition to energy generation, the regulation also covers thermal insulation, ventilation and air conditioning systems and thermal insulation of the buildings. The canton has some of the strictest insulation regulations in Switzerland. For new construction or existing heating systems renovation, 50 % of the energy for hot water must come from renewable sources. In 2008, Basel-City also began a three-year building renovation programme.
- Since 1984, the canton of Basel-City has added a 9 % tax on electricity, which is invested into renovation of buildings, promoting renewable energy and energy efficiency, awareness raising and innovation. Although electric power demand has declined continuously since mid 1990’s' the canton of Basel-City added in 1998 a steering tax (Lenkungsabgabe) on electricity. The income from this tax is redistributed at a fixed annual rate to households on a per-capita basis and to companies in relation to total paid wages.
- The solar energy exchange (Solarstrombörse) guarantees to any local producer of photovoltaic electricity that all the produced electricity can be fed into the grid of the public provider, who pays a price set at a level to fully cover all costs of the producer. These incentives promote the installation of photovoltaic systems.
- The city of Basel promotes the "2000-watt-society" with the objective to reduce the per capita energy use from the current 4000 to 2000 W without sacrificing the quality of life.

Increasing the rate of building renovations is another policy focus in Basel and this was used for URGENCHE scenarios Table 2. In comparison to Kuopio, the GHG mitigation policies of Basel are more focussed on incentives targeting individuals, households and businesses.

### Policies used in the model

The renovation and fuel change policies of Kuopio and Basel that were tested by the model are shown in Table 2 and the energy conserving potentials of different renovations in Additional file 1: Table S1–5. The energy conserving and CO_2_ reduction potentials of the policies for the Municipality of Kuopio are based on a report by Pöyry consulting [7].

### Model development

We set the following objectives to the building model development: The model should

1. reflect important factors of a city building stock needed to quantitatively estimate its heat and power demand,
2. have a modular structure so that city-specific data modules can be attached to generic computational modules to create a complete model,
3. provide connections to energy balance model and other models to be developed about other driving forces,
4. present all the data used as open linked data (i.e. in machine-readable format in the Internet),
5. only use open source code and open licenses.

### Implementation

The building model developed and tested in this study was structured to include all important parts defined by this conception. Extensive amount of building and other data was collected about the two cities. Summary tables of the most important data can be found from supporting online material (tables Additional file 1: Table S1–6). In addition, detailed data can be found from these Opasnet pages:

- Building stock data in Kuopio: data on floor areas of buildings by building type, heating type, and age; data on renovation rates [8].
- Actual climate policies in Kuopio; the case-specific online model [9].
- Building model: the actual model code calculating the history and future of the building stock, its energy need, emissions, and health impacts in the surrounding population [10].
- Building stock data in Basel: data on floor areas of buildings by building type, heating type, and age; data on renovation rates [11].
- Climate policies in Basel and the case-specific model [12].
- Energy consumption, energy efficiency, and impacts of renovation used for both case studies [13].
- Emission factors of different heating types and different plants [14].
- Health impact assessment model used to assess health risks based on exposures from the building model [15].

The building floor area in Kuopio has grown constantly since the 1950's and the trend is assumed to continue. Based on future projections of population growth and housing trends of the area we expect that the construction rate remains the same until 2050.

The building model for city assessments was developed and can be found in Opasnet (http://en.opasnet.org/w/Building_model) [10], which is an online workspace e.g. for impact assessments and other decision support (Fig. 2). The upstream modules contain city-specific building data, and the model becomes increasingly generic in the downstream. Health impact module is actually another generic Opasnet model that is compatible with the building model and uses its outputs as inputs.

Each module has one of the two essential parts: a data table containing information about the key property of that module, or a formula that can be used to compute such a data table. A key property may be e.g. the building stock amount in floor-m^2^ (Additional file 1: Table S1–7), and this is further specified by indices defining the conditions when a particular value applies. For example, the floor area may have indices Time (time of observation), Built (time when the building was built), City_area (location on the building), Building (type of the building), and Heating (heating system used in the building). Some of the indices are always necessary in the building model, but in a specific city case the user may use additional indices without any change in the model code (however, there is a cost in computing time). The data may be on individual building level, or it may be aggregated, as long as the data table columns remain the same.

Because of the modular structure, the user can replace any of the city-specific building data modules with data from another city as long as the core structure and unit of the module stays the same. This can be implemented in Opasnet e.g. by creating a new city-specific page that calls the city-specific data tables from the database and then runs the generic model. An interested user can get a user account to Opasnet and then create such a page for their own purposes.

An important functionality of the model is that the core model contains no policies but the user can define policies outside the model. When the model is run, the code automatically checks whether there are policies defined. If there are, each policy option is run as a scenario and propagated through the model. A policy is shown in the output as an index, and the different values of the index differentiate the different options. The defining of policies can be done e.g. as a table on the case-specific wiki page (the user needs to know wiki editing), or a user-friendly interface can be built so that the user can define the actual policies using drop-down menus or entry boxes (the user needs no technical skills). The relationships between the modules are deterministic, but if the inputs are given with uncertainties, the uncertainties are propagated through the model using Monte Carlo simulation and the outputs are probability distributions.

### Energy demand of building stock

Heating energy demand of the building stock was calculated based on the current average heating energy need per m^2^ in building stocks of both cities. Changes in heating energy need were predicted based on the renovation rate defined by the renovation policies presented in Table 2 and on estimation of effects of renovation level (i.e. only windows or windows, sealing of building's sheath, improvement of building's technical systems or total sheath reform including windows, sealing of building's sheath, improvement of building's technical systems and significant reform of building's sheath) on heating energy need [13]. The model takes into account only heating energy need and use of electricity for other purposes is omitted.

In Kuopio the target policies are closely linked on operation of local CHP-plant. The studied renovation policy reduces heating demand and consequently also production of district heat, which may lead to decreased CHP electricity production. This decrease needs to be compensated by producing the electricity by some other way. In this study it was assumed that rest of the electricity is bought from elsewhere and it's emissions are out of scope of this study, which is concentrating only on local effects.

### Health effect assessment

Health effects were calculated for PM_2.5_ by estimating the emissions from heat generation, transforming emissions to annual average outdoor exposure levels of local population and consequently the health effects caused by the exposure. In this study, only premature deaths were selected as target health endpoint. Emission calculations take into account the method of heating energy production and amount and type of fuel used in production. Exposure of population was calculated based on intake fractions (fraction of emitted pollutant that is eventually inhaled by the target population) by Humbert et al. [16]. It was then used for the health impact assessment combining exposure with exposure-response functions and background health incidence data. Numbers of cases were then converted into burden of disease, which is expressed as DALY/year (disability-adjusted-life-years) [17]. DALY combines years lived with disability (YLD) and years of life lost (YLL) due to premature death, of which only the latter is relevant here.

### Operation

The case studies about Kuopio and Basel were performed in Opasnet. The case-specific data, models, and user interfaces are available in Opasnet [9, 12]. Case study results can be reproduced through these pages under subheading *Model* by clicking *Run code*. Furthermore, the model code can be edited by clicking the *Show code* and copying the whole code to R on a user's local computer. The newest version of R is recommended (3.1.2).

The user interface of the model is a wiki [18]. The data was stored in a MongoDB no-SQL database [19], and the actual model code was written in R (www.cran.r-project.org version 3.1.2). The model requires OpasnetUtils and ggplot2 packages, which are freely available at the CRAN repository. All data and module downloads take place automatically when online. All code and data were released using the Creative Commons Attribute - Share alike 3.0 license.

## Results

### Kuopio

The development of the building stock in Kuopio is shown in Fig. 3. There was a large shift from single-house wood heating to district heating between 1960 and 1985. Also the typical two-storey wooden buildings in the city centre were largely replaced by six-storey apartment buildings during that period.

The business as usual (BAU) rate of renovating 3 % of the building stock per year would mean that on year 2050 almost two thirds of the built space in Kuopio could be considered energy efficient according to 2010 requirements, or better. The more ambitious active renovation policy of 4.5 %/year would increase the proportion of improved energy efficiency to more than three quarters of the total indoor space due to both renovation and construction. The higher renovation rate reflects the upper limit of technical rather than economic feasibility.

**Fig. 4:**
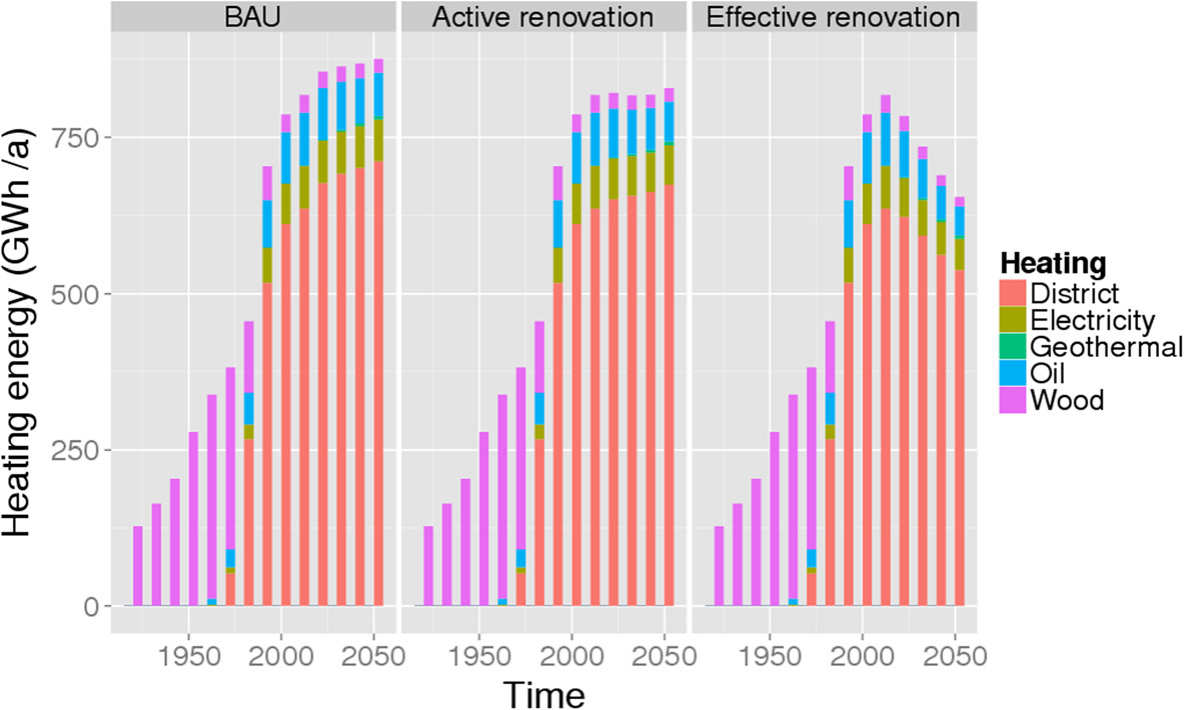
Heating energy used in Kuopio by heating type and renovation policy

The BAU renovation policy would keep the heat demand of the building stock in Kuopio nearly constant in around 800 GWh/year between 2010 and 2050, as the increased energy efficiency would cancel out the increased volume (Fig. 4.). Left panel is business as usual (3 %/year renovations), middle panel is active renovation (4.5 %/year renovations), and right panel is efficient renovation (3 %/year, sheath reform to all). Even the active renovation policy would not reduce the absolute heat demand by more than 20 % due to the projected doubling of the building stock.

**Fig. 5:**
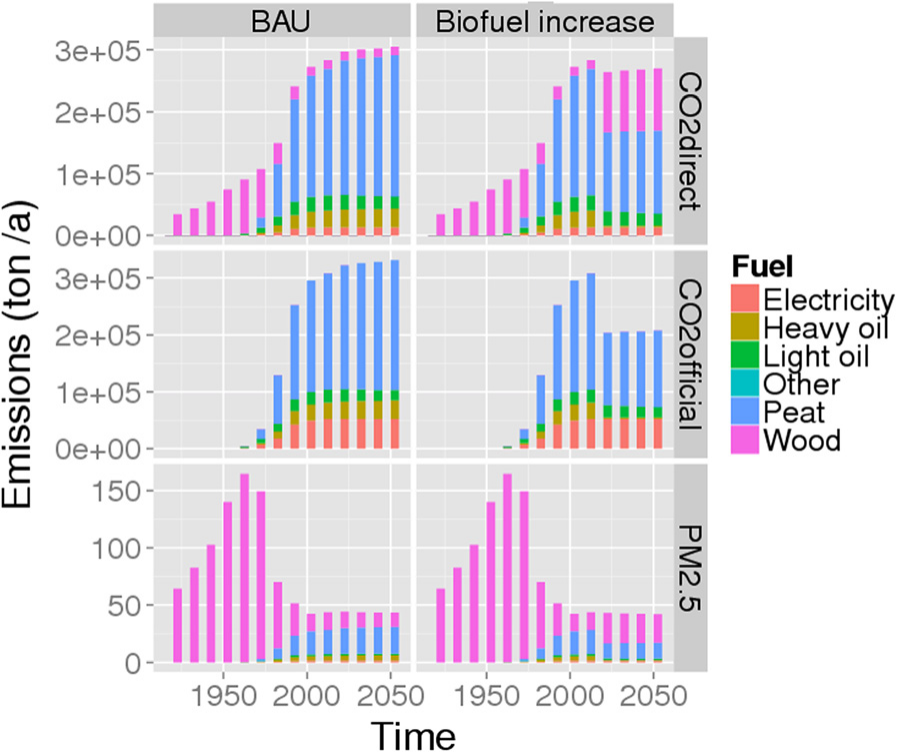
Emissions from heating in Kuopio by fuel type: estimated history and predictions 1920–2050

Due to the biofuel policy, the fossil CO_2_ emissions attributable to buildings would be reduced by a third but the respective PM_2.5_ emissions would decrease clearly less from 2010 to 2020 (Fig. 5). The total CO_2_ emission, however, would remain essentially stable for the assessment period. Left panels are for BAU, right panels are for biofuel policy. *CO*_*2*_*official* (middle horizontal panel) assumes that biofuel emissions are carbon neutral. Secondary wood heating is partly missing from the estimates although it is a substantial proportion of the current emissions.

The sum effect of renovation and biofuel policies is that premature deaths due to building related PM_2.5_ emissions decrease from 2010 by 13 % in 2020 and by 38 % in 2050 (Additional file 2). However, as already the starting level of the estimated premature deaths caused by PM_2.5_ exposure from the CHP-plant is small (around 1.5 deaths per year), the absolute effect would be minimal.

In Kuopio the assessment focused on fuel change from peat to biomass and buildings renovation (30–100 kWh/year reduction per renovated m^2^). The first demonstrates that replacing half of the peat, a fossil fuel, with wood in district heat and power cogeneration would, from 2000 to 2050, decrease the currently regulated fossil CO_2_ emission ("CO_2_ official") by a third but the total CO_2_ emission only marginally. The BAU renovation of 3 % of the building stock per year would not reduce the total heat demand, but an enhanced active renovation policy (4.5 %/year) would reduce it by a fifth. Implemented together, the policies’ impact would be multiplicative.

### Basel

**Fig. 6:**
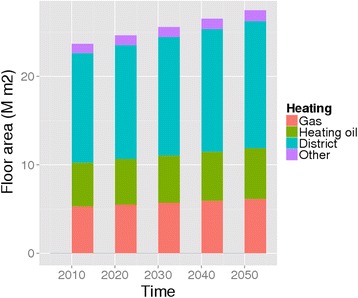
Building stock in Basel by heating type

In 2010 gas and fuel oil provided almost half of the space heating for Basel, district heating another half with a few percent from all other sources. While the overall building stock is expected to grow by over 20 % by 2030 these proportions are expected to remain essentially unchanged (Fig. 6).

**Fig. 7:**
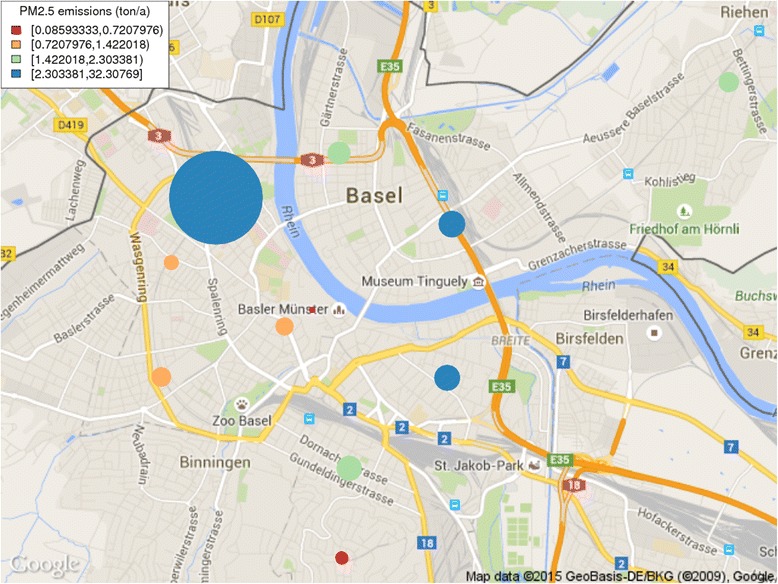
PM_2.5_ emissions from heating in Basel by postal code areas. Both the size of the sphere and the colour indicate the amount of emission

Figure 7 presents the areal PM_2.5_ emissions from space heating in Basel. The sphere size is proportional to the total emission. The largest blue sphere includes the emissions from three IWB power plants, namely Holzkraftwerk, Volta and Bahnhof. The emissions of district heating are located to the site of the power plant.

**Fig. 8:**
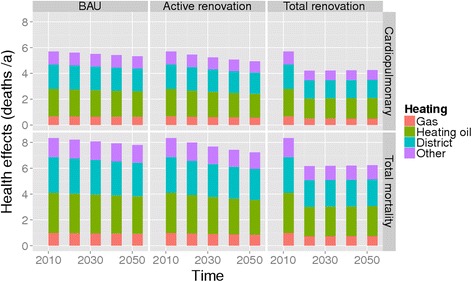
Impact on annual number of deaths attributable to heating related air pollutants (PM_2.5_). In Basel for BAU and the active renovation policy

Fossil CO_2_ contributes ca. 60 % of the total buildings related CO_2_ emissions in Basel. The Active renovation policy will not much reduce the CO_2_ emissions from 2010 to 2030, i.e. the impacts of the increased energy efficiency and the growth of the building stock almost cancel each other (Additional file 3). Total renovation policy, would, instead, reduce the GHG emissions by ca. 20 %.

In Basel the BAU renovation policy hardly reduces the PM_2.5_ emissions from the heat and power generation for the increasing building stock and respective health effects from 2010 to 2030 (Fig. 8). Doubling of the renovation rate according to the Active renovation policy, however, reduces the energy generation related mortality, although the benefit in 2050 will still remain ca. 10 %.

In Basel 80 % of all energy and 100 % of electric power is generated from renewable sources i.e. the building stock causes only marginal fossil CO_2_ emissions. Therefore, we focussed on the energy conservation impacts of the renovation (15–60 kWh/a reduction per renovated m^2^) of 1 % of the pre 1980 building stock per year in BAU, and 2 %/year in the policy scenario.

### Health effects

The assessed local public health benefits of the fuel change and energy conservation – buildings renovation – policies, compared to the BAU, are 9 disability-adjusted life years (DALY) per year in Kuopio and 15 DALY/year in Basel in 2030 (Table 3). The lack of more dramatic results is to be expected first because the thorough energy renovation of the building stock faces a huge inertia, and secondly because the increased energy efficiency of the renovated buildings will have to balance the increasing building stock. These estimates also underestimate the true policy impacts, because the renovation policy analysis does not assess the impacts on indoor environments. In addition, secondary wood heating in detached houses is a major source of PM_2.5_ in Kuopio even if it is not estimated here. There is an increasing trend to use wood in small scale combustion. This needs further scrutiny.

**Table 3:** Comparison of the health impacts of selected policies in Kuopio and Basel (DALY/a)

Time	Renovation policy	Fuel policy	Kuopio	Basel
2010	BAU	BAU	51.1	91.7
2030	BAU	BAU	47.8	88.8
2030	Active renovation	BAU	44.9	84.5
2030	Effective or total renovation	BAU	39.5	68.0
2030	BAU	Biofuel increase	47.2	96.6
2030	Active renovation	Biofuel increase	44.3	91.9
2030	Effective or total renovation	Biofuel increase	38.9	74.0

Similar studies conducted in other European cities participating in this research confirmed the two-city comparative study presented here. For example, Stuttgart demonstrated that small scale wood pellet combustion for residential heating generates only 1 % of the required heat, but from 2010 to 2025 the burden of disease could be decreased via its banning in the city centre by 15 DALY/year or be increased by its current growing trend by 200 DALY/year. An exception to this trend is in Thessaloniki, where Greek economic austerity has forced the replacement of oil and gas with cheaper wood and coal combustion, resulting in an overall decline of air quality [20].

### Importance analysis

The model can be used in two modes: a faster mode takes medians of each input probability distribution and treats the system deterministically. Most results shown were produced in this deterministic mode. With this level of detail in the data, a model run takes a few minutes or more depending on aggregation. A slower mode is to use Monte Carlo simulation to sample the input distributions, but the model structure itself is still deterministic. This of course multiplies the computing time, and for such runs, the data should be aggregated as much as possible.

To get an overview of the impact of uncertainties on the model results, we combined climate and health impacts by using nominal values of 15 €/ton for CO_2_ emissions and 50000 € per DALY for health impacts. The importance of input variables was analysed by correlating these values with input variables. We used two methods: either the values were compared as such, or the BAU option was first subtracted to correlate variables to incremental values (Additional file 4). The analyses produced multiple correlations for each variable, as there are several combinations of decision options. The results were produced with 1000 iterations (which took an hour) and are shown on Table 4.

**Table 4:** Importance analysis of the model

Variable	Impact values used in correlation	
	Absolute values	Incremental values relative to BAU
Exposure-response function of PM_2.5_	0.75-0.76	0-0.74
Shares of different heating types in the future	0.03-0.05	0.01-0.05
Amount of houses constructed in the future	0.02-0.03	0.01-0.05
Energy need of low-energy buildings	0.02-0.03	0.01-0.06
Shares of low-energy buildings in the future	0-0.01	0-0.01
Emission factors for PM_2.5_	0-0.01	0.05-0.05
Shares of renovation types in the future	0	0

The values are ranges of absolute rank correlations between the outcome and different input variables

We did not attempt to cover all input variables probabilistically. Rather, we described enough probabilities to demonstrate the functionality of the model and possibly identify development needs. For example, uncertainty on the CO_2_ life cycle emission factor of wood-based fuels should be quantified and elaborated in the future.

## Discussion

We developed and applied a model for estimating public health and CO_2_ emission effects of the energy consumption of an urban buildings stock. We used the model to produce guidance for actual policy questions by comparing options with city-specific data. The model successfully estimates health and climate impacts and helps to understand them.

The technical objectives were also achieved: the model follows the building stock in time, makes comparisons between scenarios, propagates uncertainties, and scales to different levels of detail. It estimates heat and power demand by using a modular structure, open linked data, and open licenses. Although in the URGENCHE project the model was operated by health experts, this is no longer needed as the health expertise is already embedded in the model structure.

A city-level view is critical when assessing options that can be decided and implemented on city level. For example, renovation policies are straightforward in the sense that if heating energy is saved locally, it also reduces respective climate impacts globally. However, other local decisions such as changes in combined heat and power production in one area might lead to energy balance complications nationally: If the city of Helsinki replaced CHP production with biofuel heat plants, that would lead to increased need for national electricity generation. This might have negative climate impacts depending on who would produce the electric power and how. This is a real-life example that is currently being assessed using the models described in this article [21].

The assessments were performed in collaboration with the cities of Kuopio and Basel, and the models informed decision makers of the cities. However, the models were not yet flexible and user-friendly enough to actually enable non-modellers to develop and test different policy options. That functionality is being improved and tested in the Helsinki assessment mentioned above. It requires modelling skills to adapt the model for a new city. However, already in the Helsinki assessment a non-expert can adjust decision options and run the model online using a simple user interface.

A major development need is to increase the user-friendliness and applicability of the model so that it cannot only be run but also set up for a new city by consultants or city authorities. This is already possible if there is enough interest and a few weeks work-time available. However, we have noticed that a critical factor is awareness that such modelling assessments could and should be performed to support practical decision making in cities. The case of Helsinki seems to promote this idea.

Of the URGENCHE cities Kuopio and Basel provided detailed data on city's building stock. Therefore, the actual modeling was tested for these two cities. However, the developed urban building stock model can be used for a city even if there is only aggregate data available. The assessed policies - not the model - determine how detailed data are needed for useful calculations.

Kuopio has a climate objective to reduce GHG emissions by 40 % between 1990 and 2020. This is a major challenge but it seems to be achievable if large fuel changes from peat to wood-based fuels are done in the district heating. However, this policy is based on the assumption that wood-based fuels are climate neutral. This assumption should be further studied, as different biofuels may differ widely in this respect. Health impacts of all studied policies are small and therefore not a major concern.

Basel also has an ambitious objective to reach 2000 watt society. Based on our analysis, it will be difficult to reduce energy consumption of the building stock. However, improvements are possible with systematic long-term policy. Indoor air issues should be kept in mind when renovating buildings.

Indoor environmental quality is a critical issue when considering health effects of indoor environments. The realism and even desirability of drastic building renovation policies raises questions. Very ambitious and schematic insulation and ventilation renovations with insufficient consideration of the energy and moisture physics of the different old individual buildings have caused material damage to many buildings and health problems to their occupants in the past. Furthermore, levels of indoor pollutants are affected by ventilation and there is a risk that energy savings by decreasing ventilation may cause higher exposures and increase harmful health effects.

Unfortunately, the model does not yet include indoor air quality aspects, which include the introduction of indoor air toxicants off-gassed from building finishing and decorative materials, a result of Chinese urban affluence and growing consumer preferences. However, thermal comfort in houses of Kuopio was evaluated separately by a questionnaire and a qualitative assessment. This evaluation indicated that thermal comfort is an important issue for well-being of the residents in the current building stock of Kuopio. A part of the residences are perceived as too cold in the winter and a bigger part too warm in the summer. Increased insulation required by the energy efficiency regulation could, for many, improve comfort in the winter but, without improved air exchange, decrease the thermal comfort during the summer.

## Conclusions

In conclusion, we developed an online model that fulfils its objectives and is capable of producing useful guidance on practical building-related policy questions on city level. Especially, it informs decision makers about public health and climate consequences of urban building and GHG mitigation policies.

In the assessed cases, all considered decision options had minimal health impacts of PM_2_ emissions as the current district heating systems are already clean. In addition, energy demand of building stock can be effectively reduced only with systematic policies that are implemented for decades. The model did not yet produce satisfactory guidance about indoor environmental quality and life cycle CO_2_ emissions of wood-based fuels, and these were identified as development needs.

Although the model is openly available, it requires some computing skills to adapt it to a new city. However, it offers the core functionality that can be used by a resourceful city. It also demonstrates the usefulness and need of such open web-based assessment models.

### Data and software availability

All data and software are published and available in Opasnet with Creative Commons Attribute - Share alike 3.0 license. The following pieces of data are available:

- Kuopio assessment description and data: http://en.opasnet.org/w/Climate_change_policies_and_health_in_Kuopio
- Basel assessment description and data: http://en.opasnet.org/w/Climate_change_policies_in_Basel
- Kuopio model output: http://en.opasnet.org/en-opwiki/index.php?title=Special:RTools&id=PWq7mHEWjyFReXDV
- Basel model output: http://en.opasnet.org/en-opwiki/index.php?title=Special:RTools&id=AOqn0FIvJu1Wxidg
- Kuopio importance analysis: http://en.opasnet.org/en-opwiki/index.php?title=Special:RTools&id=1zu5BF0w5a3miRtv
- You can even download all the R objects created in the assessment to your own computer and examine them. You must have R software and OpasnetUtils package installed (from https://www.r-project.org/). In the console, enter there commands:
- objects.get("PWq7mHEWjyFReXDV") # (for Kuopio)
- objects.get("AOqn0FIvJu1Wxidg") # (for Basel)
- objects.get("V3K6ePJVE3oinNyP") # (for Kuopio importance analysis with 1000 iterations)

## Additional files

Additional file 1:
**Summaries of input data used in the model.** 7 additional data tables of input data: Table S1 Building statistics of Kuopio in year 2010. Table S2 Building statistics of Basel in year 2010. Table S3 Energy demand of buildings in Kuopio in year 2010. Table S4 Energy demand of buildings in Basel in year 2010. Table S5 Energy saving potentials of different renovations in Basel and Kuopio. Table S6 PM2.5 and CO2 emission factors for different fuels and burners (mg/MJ). Direct CO2 is what comes out of the stack. We also calculated CO2official where biofuels (wood and waste) were assumed to be carbon neutral. Table S7 An example of an output table (data.frame in R) from the model: the first rows (out ot 67680) of the modelled building stock for Basel. (PDF 346 kb) Additional file 2:
**Model run for Kuopio.** R-model run for Kuopio case with the R-code and the results. (PDF 878 kb) Additional file 3:
**Model run for Basel.** R-model run for Basel case with the R-code and the results. (PDF 1558 kb) Additional file 4:
**Importance analysis for Kuopio with 1000 iterations.** R-model run for the importance analysis for Kuopio case with the R-code and results. (PDF 245 kb)

## Abbreviations

BAU: Business as usual
DALY: Disability-adjusted life year
EU FP7: European Union 7th Framework Program
GHG: Greenhouse gas
Mt: Megaton = 1,000,000,000 kg
MWt: Megawatts of thermal energy
NO_x_: Oxides of nitrogen (mainly NO and NO_2_)
PM_2.5_: Particulate matter smaller than 2.5 micrometres
