# Supplementary material for: Building-related health impacts in European and Chinese cities: a scalable assessment method
Source: Environ Health. 2015 Dec 14;14:93. doi: 10.1186/s12940-015-0082-z (PMC4678713; doi:10.1186/s12940-015-0082-z)
Supplement: Additional file 1: — Summaries of input data used in the model. 7 additional data tables of input data: Table S1 Building statistics of Kuopio in year 2010. Table S2 Building statistics of Basel in year 2010. Table S3 Energy demand of buildings in Kuopio in year 2010. Table S4 Energy demand of buildings in Basel in year 2010. Table S5 Energy saving potentials of different renovations in Basel and Kuopio. Table S6 PM2.5 and CO2 emission factors for different fuels and burners (mg/MJ). Direct CO2 is what comes out of the stack. We also calculated CO2official where biofuels (wood and waste) were assumed to be carbon neutral. Table S7 An example of an output table (data.frame in R) from the model: the first rows (out ot 67680) of the modelled building stock for Basel. (PDF 346 kb) [file 12940_2015_82_MOESM1_ESM.pdf]

Additional data tables

Table Additional file 1-1. Building statistics of Kuopio in year 2010.

**Building**	**Floor area**
Apartment houses	2417060.9
Commercial	553297.0
Detached houses	858954.8
Educational	412216.2
Health and social sector	270451.1
Industrial	319897.1
Leisure houses	484323.0
Offices	330593.0
Other	245381.1
Public	92082.9
Row houses	499020.2
Sports	95904.8
TOTAL	6579182

Table Additional file 1-2. Building statistics of Basel in year 2010.

Building class	Floor area	Number of buildings
Residential building with one dwelling	2272971	9719
Residential building with two dwellings	487379	1086
Residential building with three ore more dwellings	11682636	11898
Residential building for communities	482472	129
Hotels	399793	88
Other buildings for short host stay	26898	13
Office building	2458193	614
Retail and wholesale trade buildings	917470	182
Public transport stations, communication related buildings	123497	25
Garage	87287	63
Industry	1107927	185
Storage buildings	167818	58
Cultural and leisure related buildings	176756	71
Museum, libraries	135030	29
Schloos, Universities, Science buildings	643841	202
Hospitals and other health related buildings	627517	87
Sports halls (buildings)	9491	4
Agricultural buildings	19649	20

Church and other cultural buildings	96467	50
All other buildings	48324	75
TOTAL	23746143	25588

Table Additional file 1-3. Energy demand of buildings in Kuopio in year 2010.

**Building type**	**Heat (kWh/m2/a)**	**User electricity (kWh/m2/a)**	**Total electricity (kWh/m2/a)**
Detached houses	135	50	185
Row houses	169	74	74
Apartment houses	172	42	42
Commercial	162	230	230
Offices	161	93	93
Health and social sector	215	123	123
Public	165	110	110
Sports	121	86	86
Educational	170	116	116
Industrial	168	212	212
Other	138	170	170

Table Additional file 1-4. Energy demand of buildings in Basel in year 2010.

Use	Building type	Space heating demand (kWh/m2)	Warm water demand (kWh/m2)	Electricity demand (kWh/m2)
Working	Commercial building	113	123	8
	Industry	113	123	8
Mixed use	Old buildings <1840	95	20	31
	Buildings <1938	86	20	31
	Villas <1938	106	20	31
Residential	Multifamily house <1938	106	20	31
	Social flats >1950	111	20	31
	Building towers	104	20	31
	Flats 1960-80	97	20	31
	Flats since 1990	54	20	31
	Single-family house	69	13	31
	New buildings	63	20	31

Table Additional file 1-5. Energy saving potentials of different renovations in Basel and Kuopio.

Renovation	Relative saving (%)	Renovation details
General	15	General renovation
Windows	15	New windows and doors
Technical systems	50	New windows, sealing of building's sheath, improvement of building's technical systems
Sheath reform	65	New windows, sealing of building's sheath, improvement of building's technical systems, significant reform of building's sheath

Table Additional file 1-6. $PM_{2.5}$ and CO_2 emission factors for different fuels and burners (mg/MJ). Direct CO_2 is what comes out of the stack. We also calculated *CO_2official* where biofuels (wood and waste) were assumed to be carbon neutral.

**Burner**	**Fuel**	**$PM_{2.5}$**	**Direct CO_2**	**Description and assumptions**
Domestic	Wood	140 (65.8-263)	74200	Other stoves and ovens. Karvosenoja et al. 2008
Domestic	Light oil	0-10	74200	Light oil <5 MW Emission factors for burning processes. Light oil 267 kg /MWh
Domestic	Other sources	0-10	74200	Same as oil.
Domestic	Gas	0-3	55650	For $PM_{2.5}$: one third of that of oil. For CO_2: 3/4 of that of oil.
Large fluidized bed	Wood	2-20	74200	Large plant (100-300 MW). Karvosenoja et al., 2008
Large fluidized bed	Peat	2-20	106000	100-300 MW
Large fluidized bed	Heavy oil	8-22	106000	100-300 MW
Large fuildized bed	Gas	0-3	55650	For $PM_{2.5}$: one third of that of oil. For CO_2: 3/4 of that of oil.
Large fluidized bed	Coal	2-20	106000	Same as peat.
Large fluidized bed	Waste	2-20	74200	Same as wood.
Grid	Electricity	01-10	53000	50 % of large-scale burning (because of nuclear and hydro). Heavy oil 279 kg /MWh. Officially, electricity is not CHP but requires a double amount of coal to produce it.

Table Additional file 1-7. An example of an output table (data.frame in R) from the model: the first rows (out ot 67680) of the modelled building stock for Basel.

Row	Time	City_area	Building	Heating	Renovation	RenovationPolicy	buildingsResult	buildingsSource
1	2010	4055	Buildings with partial residential use	Centrifuge, hydro-extractor	None	Active renovation	4485.6	Formula
2	2010	4052	Multiple-family houses	Centrifuge, hydro-extractor	None	Active renovation	352.8	Formula
3	2010	4055	Multiple-family houses	Centrifuge, hydro-extractor	None	Active renovation	1078.2	Formula
4	2010	4054	Residential buildings with subsidiary use	Centrifuge, hydro-extractor	None	Active renovation	109.8	Formula
5	2010	4055	Residential buildings with subsidiary use	Centrifuge, hydro-extractor	None	Active renovation	1658.4	Formula
6	2010	4055	Single-family houses	Centrifuge, hydro-extractor	None	Active renovation	72	Formula
