# Supplementary material for: Building-related health impacts in European and Chinese cities: a scalable assessment method
Source: Environ Health. 2015 Dec 14;14:93. doi: 10.1186/s12940-015-0082-z (PMC4678713; doi:10.1186/s12940-015-0082-z)
Supplement: Additional file 2: — Model run for Kuopio. R-model run for Kuopio case with the R-code and the results. (PDF 878 kb) [file 12940_2015_82_MOESM2_ESM.pdf]

R-tools

Ran at 2015-07-24 05:24:26

```
> wiki_username <- "Jouni"
> server <- TRUE
> ### THIS CODE IS FROM PAGE [[Climate change policies and health in Kuopio]] (Op_en5461, code_name = "")
> library(OpasnetUtils)
> library(ggplot2)
> ### Technical parameters
> openv.setN(0) # use medians instead of whole sampled distributions
> objects.latest("Op_en6007", code_name = "answer") # [[OpasnetUtils/Drafts]] findrest
> BS <- 24 # base_size = font sixe in graphs
> figstofile <- FALSE
> saveobjects <- TRUE
> finnish <- FALSE
> suomenna <- function(ova) {
+ if(class(ova) == "ovariable") out <- ova@output else out <- ova
+ if("Heating" %in% colnames(out)) {
+ out$Heating <- as.factor(out$Heating)
+ levels(out$Heating)[levels(out$Heating) == "District heating"] <- "District"
+ }
+ if("Response" %in% colnames(out)) {
+ out$Response <- as.factor(out$Response)
+ levels(out$Response)[levels(out$Response) == "Cardiopulmonary mortality"] <- "Cardiopulmonary"
+ }
+ if("Pollutant" %in% colnames(out)) {
+ out$Pollutant <- as.factor(out$Pollutant)
+ levels(out$Pollutant)[levels(out$Pollutant) == "CO2trade"] <- "CO2official"
+ }
+ out$Time <- as.numeric(as.character(out$Time))
+ return(out)
+ }
> obstime <- Ovariable("obstime", data = data.frame(Obsyear = factor(seq(1920, 2050, 10), ordered = TRUE), Result = 1))
> ## Additional index needed in followup of ovariables efficiencyShares and stockBuildings
> year <- Ovariable("year", data = data.frame(
+ Constructed = factor(
+ c("1799-1899", "1900-1909", "1910-1919", "1920-1929", "1930-1939", "1940-1949",
+ "1950-1959", "1960-1969", "1970-1979", "1980-1989", "1990-1999",
+ "2000-2010", "2011-2019", "2020-2029", "2030-2039", "2040-2049"
+ ),
+ ordered = TRUE
+ ),
+ Time = c(1880, 1910 + 0:14 * 10),
+ Result = 1
+ ))
> ###################### Decisions
> decisions <- opbase.data('Op_en5461', subset = "Decisions") # [[Climate change policies and health in Kuopio]]
> DecisionTableParser(decisions)
> # Remove previous decisions, if any.
> forgetDecisions <- function() {
+ for(i in ls(envir = openv)) {
+ if("dec_check" %in% names(openv[[i]])) openv[[i]]$dec_check <- FALSE
+ }
+ return(cat("Decisions were forgotten.\n"))
+ }
> forgetDecisions()
Decisions were forgotten.

> ############################ IMPORT DATA AND MODELS
> objects.latest("Op_en5417", code_name = "initiate") # [[Population of Kuopio]]
> # population: City_area
> objects.latest("Op_en5932", code_name = "initiatetest") # [[Building stock in Kuopio]] Building ovariables:
> # buildingStock: Building, Constructed, City_area
> # rateBuildings: Age, (RenovationPolicy)
> # renovationShares: Renovation
> # construction: Building
> # constructionAreas: City_area
> # buildingTypes: Building, Building2
> # heatingShares: Building, Heating, Eventyear
> # heatingSharesNew: Building2, Heating
> # eventyear: Constructed, Eventyear
> ###################### Actual building model
> # The building stock is measured as m^2 floor area.
> objects.latest("Op_en6289", code_name = "buildingstest") # [[Building model]] # Generic building model.
> ###################### Energy and emissions
> objects.latest("Op_en5488", code_name = "energyUseAnnual") # [[Energy use of buildings]] energyUse
> objects.latest("Op_en5488", code_name = "efficiencyShares") # [[Energy use of buildings]]
> objects.latest("Op_en2791", code_name = "emissionstest") # [[Emission factors for burning processes]]
> objects.latest("Op_en2791", code_name = "emissionFactors") # [[Emission factors for burning processes]]
```

```
> objects.latest("Op_en7328", code_name = "emissionLocations") # [[Kuopio energy production]]
> objects.latest("Op_en7328", code_name = "fuelShares") # [[Kuopio energy production]]
> objects.latest("Op_en5141", code_name = "fuelUse") # [[Energy balance]]
> ## Exposure
> objects.latest("Op_en5813", code_name = "exposure") # [[Intake fractions of PM]] uses Humbert iF as default.
> ###################### Health assessment
> objects.latest('Op_en2261', code_name = 'totcases') # [[Health impact assessment]] totcases and dependencies.
> objects.latest('Op_en5461', code_name = 'DALYs') # [[Climate change policies and health in Kuopio]] DALYs, DW, L
> frexposed <- 1 # fraction of population that is exposed
> bgexposure <- 0 # Background exposure to an agent (a level below which you cannot get in practice)
> BW <- 70 # Body weight (is needed for RR calculations although it is irrelevant for PM2.5)
> ###################### CALCULATIONS
> renovationRate <- EvalOutput(renovationRate) * 10 # Rates for 10-year periods
> renovationRate@marginal[colnames(renovationRate@output) == "Age"] <- TRUE
> renovationShares <- EvalOutput(renovationShares)
> colnames(renovationShares@output)[colnames(renovationShares@output) == "Startyear"] <- "Obsyear"
> stockBuildings <- EvalOutput(stockBuildings)
> stockBuildings <- oapply(stockBuildings, cols = c("City_area"), FUN = sum)
> changeBuildings <- EvalOutput(changeBuildings)
[1] "Column EfficiencyPolicy treated with fillna (difference between historical buildings and construction)."

> changeBuildings <- oapply(changeBuildings, cols = c("City_area"), FUN = sum)
> buildings <- EvalOutput(buildings)
[1] "Column RenovationPolicy treated with fillna (difference between buil and renovate)."

> buildings@output$RenovationPolicy <- factor(
+ buildings@output$RenovationPolicy,
+ levels = c("BAU", "Active renovation", "Effective renovation"),
+ ordered = TRUE
+ )
> buildings@output$EfficiencyPolicy <- factor(
+ buildings@output$EfficiencyPolicy,
+ levels = c("BAU", "Active efficiency"),
+ ordered = TRUE
+ )
> energyUse <- EvalOutput(energyUse)
> fuelUse <- EvalOutput(fuelUse)
> fuelUse <- fuelUse * 1E-3 *3600 # kWh -> MJ
> emissions <- EvalOutput(emissions)
> population <- 1E+5 # stockBuildings is using another population to divide floor area into City areas.
> exposure <- EvalOutput(exposure)
> exposure@output <- exposure@output[exposure@output$Area == "Average" , ] # Kuopio is an average area,
> # rather than rural or urban.
> totcases <- EvalOutput(totcases)
> totcases <- oapply(totcases, cols = c("Age", "Sex"), FUN = sum)
> DALYs <- EvalOutput(DALYs)
> ###################### GRAPHS AND OUTPUTS
> bui <- suomenna(oapply(buildings * 1E-6, cols = c("City_area", "buildingsSource"), FUN = sum))
> ggplot(subset(bui, RenovationPolicy == "BAU" & EfficiencyPolicy == "BAU"), aes(x = Time, weight = buildingsResult, fill = Heating)) +
geom_bar(binwidth = 5) +
+ theme_gray(base_size = BS) +
+ labs(
+ title = "Building stock in Kuopio",
+ x = "Time",
+ y = "Floor area (M m2)"
+ )
> if(figstofile) ggsave("Figure3.eps", width = 8, height = 7)
> ggplot(bui, aes(x = Time, weight = buildingsResult, fill = Building))+geom_bar()+facet_grid(Efficiency~Heating)
> ggplot(subset(bui, EfficiencyPolicy == "BAU"), aes(x = Time, weight = buildingsResult, fill = Renovation)) +
+ geom_bar(binwidth = 5) +
+ facet_grid(. ~ RenovationPolicy) + theme_gray(base_size = BS) +
+ labs(
+ title = "Building stock in Kuopio by renovation policy",
+ x = "Time",
+ y = "Floor area (M m2)"
+ )
> ggplot(subset(bui, RenovationPolicy == "BAU"), aes(x = Time, weight = buildingsResult, fill = Efficiency)) + geom_bar(binwidth = 5) +
+ facet_grid(. ~ EfficiencyPolicy) + theme_gray(base_size = BS) +
+ labs(
+ title = "Building stock in Kuopio by efficiency policy",
+ x = "Time",
+ y = "Floor area (M m2)"
+ )
> ggplot(subset(bui, RenovationPolicy == "BAU" & EfficiencyPolicy == "BAU"), aes(x = Time, weight = buildingsResult, fill = Building)) +
geom_bar(binwidth = 5) +
+ theme_gray(base_size = BS) +
+ labs(
+ title = "Building stock in Kuopio",
+ x = "Time",
+ y = "Floor area (M m2)"
+ )
> ggplot(subset(suomenna(energyUse), EfficiencyPolicy == "BAU"), aes(x = Time, weight = energyUseResult * 1E-6, fill = Heating)) + geom_bar(binwidth =
```

```
5) +
+ facet_wrap( ~ RenovationPolicy) + theme_gray(base_size = BS) +
+ labs(
+ title = "Energy used in heating in Kuopio",
+ x = "Time",
+ y = "Heating energy (GWh /a)"
+ )
> if(figstofile) ggsave("Figure4.eps", width = 11, height = 7)
> ggplot(suomenna(energyUse), aes(x = Time, weight = energyUseResult * 1E-6, fill = Heating)) + geom_bar(binwidth = 5) +
+ facet_grid(EfficiencyPolicy ~ RenovationPolicy) + theme_gray(base_size = BS) +
+ labs(
+ title = "Energy used in heating in Kuopio",
+ x = "Time",
+ y = "Heating energy (GWh /a)"
+ )
> emis <- suomenna(truncateIndex(emissions, cols = "Fuel", bins = 5))
> ggplot(subset(emis, EfficiencyPolicy == "BAU" & RenovationPolicy == "BAU" & Pollutant != "CO2eq"), aes(x = Time, weight = emissionsResult, fill =
Fuel)) + geom_bar(binwidth = 5) +
+ facet_grid(Pollutant ~ FuelPolicy, scale = "free_y") + theme_gray(base_size = BS) +
+ labs(
+ title = "Emissions from heating in Kuopio",
+ x = "Time",
+ y = "Emissions (ton /a)"
+ )
> if(figstofile) ggsave("Figure5.eps", width = 8, height = 7)
> ggplot(subset(emis, EfficiencyPolicy == "BAU" & RenovationPolicy == "BAU"), aes(x = Time, weight = emissionsResult, fill = Fuel)) +
geom_bar(binwidth = 5) +
+ facet_grid(Pollutant ~ ., scale = "free_y") + theme_gray(base_size = BS) +#FuelPolicy
+ labs(
+ title = "Emissions from heating in Kuopio",
+ x = "Time",
+ y = "Emissions (ton /a)"
+ )
> ggplot(subset(emis, EfficiencyPolicy == "BAU" & FuelPolicy == "BAU"), aes(x = Time, weight = emissionsResult, fill = Emission_site)) +
geom_bar(binwidth = 5) +
+ facet_grid(Pollutant ~ RenovationPolicy, scale = "free_y") + theme_gray(base_size = BS) +
+ labs(
+ title = "Emissions from heating in Kuopio",
+ x = "Time",
+ y = "Emissions (ton /a)"
+ )
> ggplot(subset(emis, EfficiencyPolicy == "BAU" & FuelPolicy == "BAU"), aes(x = Time, weight = emissionsResult, fill = Fuel)) + geom_bar(binwidth = 5)
+
+ facet_grid(Pollutant ~ RenovationPolicy, scale = "free_y") + theme_gray(base_size = BS) +
+ labs(
+ title = "Emissions from heating in Kuopio",
+ x = "Time",
+ y = "Emissions (ton /a)"
+ )
> ggplot(subset(suomenna(exposure), RenovationPolicy == "BAU" & EfficiencyPolicy == "BAU" & FuelPolicy == "BAU"), aes(x = Time, weight =
exposureResult, fill = Heating)) +
+ geom_bar(binwidth = 5) + facet_grid(Area ~ Emission_height) + theme_gray(base_size = BS) +
+ labs(
+ title = "Exposure to PM2.5 from heating in Kuopio",
+ x = "Time",
+ y = "Average PM2.5 (µg/m3)"
+ )
> ggplot(subset(suomenna(exposure), EfficiencyPolicy == "BAU"), aes(x = Time, weight = exposureResult, fill = Heating)) + geom_bar(binwidth = 5) +
facet_grid(FuelPolicy ~ RenovationPolicy) + theme_gray(base_size = BS) +
+ labs(
+ title = "Exposure to PM2.5 from heating in Kuopio",
+ x = "Time",
+ y = "Average PM2.5 (µg/m3)"
+ )
> ggplot(subset(suomenna(totcases), EfficiencyPolicy == "BAU" & FuelPolicy == "BAU"), aes(x = Time, weight = totcasesResult, fill =
Heating))+geom_bar(binwidth = 5) +
+ facet_grid(Response ~ RenovationPolicy) +
+ theme_gray(base_size = BS) +
+ labs(
+ title = "Health effects of PM2.5 from heating in Kuopio",
+ x = "Time",
+ y = "Health effects (deaths /a)"
+ )
> cat("Total DALYs/a by different combinations of policy options.\n")
Total DALYs/a by different combinations of policy options.

> dal <- subset(suomenna(DALYs), Response == "Total mortality")
> oprint(aggregate(dal["DALYsResult"], by = dal[c("Time", "EfficiencyPolicy", "RenovationPolicy", "FuelPolicy")], FUN = sum))
```

	Time	EfficiencyPolicy	RenovationPolicy	FuelPolicy	DALYsResult
1	1920.00	BAU	BAU	BAU	139.77

2	1930.00	BAU	BAU	BAU	179.32
3	1940.00	BAU	BAU	BAU	222.64
4	1950.00	BAU	BAU	BAU	304.28
5	1960.00	BAU	BAU	BAU	357.78
6	1970.00	BAU	BAU	BAU	321.03
7	1980.00	BAU	BAU	BAU	134.56
8	1990.00	BAU	BAU	BAU	76.35
9	2000.00	BAU	BAU	BAU	50.07
10	2010.00	BAU	BAU	BAU	51.08
11	2020.00	BAU	BAU	BAU	49.75
12	2030.00	BAU	BAU	BAU	47.81
13	2040.00	BAU	BAU	BAU	46.32
14	2050.00	BAU	BAU	BAU	45.38
15	1920.00	Active efficiency	BAU	BAU	139.77
16	1930.00	Active efficiency	BAU	BAU	179.32
17	1940.00	Active efficiency	BAU	BAU	222.64
18	1950.00	Active efficiency	BAU	BAU	304.28
19	1960.00	Active efficiency	BAU	BAU	357.78
20	1970.00	Active efficiency	BAU	BAU	321.03
21	1980.00	Active efficiency	BAU	BAU	134.56
22	1990.00	Active efficiency	BAU	BAU	76.35
23	2000.00	Active efficiency	BAU	BAU	50.07
24	2010.00	Active efficiency	BAU	BAU	51.08
25	2020.00	Active efficiency	BAU	BAU	49.67
26	2030.00	Active efficiency	BAU	BAU	47.56
27	2040.00	Active efficiency	BAU	BAU	45.95
28	2050.00	Active efficiency	BAU	BAU	45.04
29	1920.00	BAU	Active renovation	BAU	139.77
30	1930.00	BAU	Active renovation	BAU	179.32
31	1940.00	BAU	Active renovation	BAU	222.64
32	1950.00	BAU	Active renovation	BAU	304.28
33	1960.00	BAU	Active renovation	BAU	357.78
34	1970.00	BAU	Active renovation	BAU	321.03
35	1980.00	BAU	Active renovation	BAU	134.56
36	1990.00	BAU	Active renovation	BAU	76.35
37	2000.00	BAU	Active renovation	BAU	50.07
38	2010.00	BAU	Active renovation	BAU	51.08
39	2020.00	BAU	Active renovation	BAU	47.51
40	2030.00	BAU	Active renovation	BAU	44.88
41	2040.00	BAU	Active renovation	BAU	43.36
42	2050.00	BAU	Active renovation	BAU	42.72
43	1920.00	Active efficiency	Active renovation	BAU	139.77
44	1930.00	Active efficiency	Active renovation	BAU	179.32
45	1940.00	Active efficiency	Active renovation	BAU	222.64
46	1950.00	Active efficiency	Active renovation	BAU	304.28
47	1960.00	Active efficiency	Active renovation	BAU	357.78
48	1970.00	Active efficiency	Active renovation	BAU	321.03
49	1980.00	Active efficiency	Active renovation	BAU	134.56
50	1990.00	Active efficiency	Active renovation	BAU	76.35
51	2000.00	Active efficiency	Active renovation	BAU	50.07
52	2010.00	Active efficiency	Active renovation	BAU	51.08
53	2020.00	Active efficiency	Active renovation	BAU	47.42
54	2030.00	Active efficiency	Active renovation	BAU	44.63
55	2040.00	Active efficiency	Active renovation	BAU	42.99
56	2050.00	Active efficiency	Active renovation	BAU	42.39
57	1920.00	BAU	Effective renovation	BAU	139.77
58	1930.00	BAU	Effective renovation	BAU	179.32
59	1940.00	BAU	Effective renovation	BAU	222.64
60	1950.00	BAU	Effective renovation	BAU	304.28

61	1960.00	BAU	Effective renovation	BAU	357.78
62	1970.00	BAU	Effective renovation	BAU	321.03
63	1980.00	BAU	Effective renovation	BAU	134.56
64	1990.00	BAU	Effective renovation	BAU	76.35
65	2000.00	BAU	Effective renovation	BAU	50.07
66	2010.00	BAU	Effective renovation	BAU	51.08
67	2020.00	BAU	Effective renovation	BAU	45.01
68	2030.00	BAU	Effective renovation	BAU	39.48
69	2040.00	BAU	Effective renovation	BAU	35.14
70	2050.00	BAU	Effective renovation	BAU	32.04
71	1920.00	Active efficiency	Effective renovation	BAU	139.77
72	1930.00	Active efficiency	Effective renovation	BAU	179.32
73	1940.00	Active efficiency	Effective renovation	BAU	222.64
74	1950.00	Active efficiency	Effective renovation	BAU	304.28
75	1960.00	Active efficiency	Effective renovation	BAU	357.78
76	1970.00	Active efficiency	Effective renovation	BAU	321.03
77	1980.00	Active efficiency	Effective renovation	BAU	134.56
78	1990.00	Active efficiency	Effective renovation	BAU	76.35
79	2000.00	Active efficiency	Effective renovation	BAU	50.07
80	2010.00	Active efficiency	Effective renovation	BAU	51.08
81	2020.00	Active efficiency	Effective renovation	BAU	44.93
82	2030.00	Active efficiency	Effective renovation	BAU	39.24
83	2040.00	Active efficiency	Effective renovation	BAU	34.77
84	2050.00	Active efficiency	Effective renovation	BAU	31.72
85	1920.00	BAU	BAU	Biofuel increase	139.77
86	1930.00	BAU	BAU	Biofuel increase	179.32
87	1940.00	BAU	BAU	Biofuel increase	222.64
88	1950.00	BAU	BAU	Biofuel increase	304.28
89	1960.00	BAU	BAU	Biofuel increase	357.78
90	1970.00	BAU	BAU	Biofuel increase	321.03
91	1980.00	BAU	BAU	Biofuel increase	134.56
92	1990.00	BAU	BAU	Biofuel increase	76.35
93	2000.00	BAU	BAU	Biofuel increase	50.07
94	2010.00	BAU	BAU	Biofuel increase	51.08
95	2020.00	BAU	BAU	Biofuel increase	49.11
96	2030.00	BAU	BAU	Biofuel increase	47.16
97	2040.00	BAU	BAU	Biofuel increase	45.66
98	2050.00	BAU	BAU	Biofuel increase	44.71
99	1920.00	Active efficiency	BAU	Biofuel increase	139.77
100	1930.00	Active efficiency	BAU	Biofuel increase	179.32
101	1940.00	Active efficiency	BAU	Biofuel increase	222.64
102	1950.00	Active efficiency	BAU	Biofuel increase	304.28
103	1960.00	Active efficiency	BAU	Biofuel increase	357.78
104	1970.00	Active efficiency	BAU	Biofuel increase	321.03
105	1980.00	Active efficiency	BAU	Biofuel increase	134.56
106	1990.00	Active efficiency	BAU	Biofuel increase	76.35
107	2000.00	Active efficiency	BAU	Biofuel increase	50.07
108	2010.00	Active efficiency	BAU	Biofuel increase	51.08
109	2020.00	Active efficiency	BAU	Biofuel increase	49.03
110	2030.00	Active efficiency	BAU	Biofuel increase	46.92
111	2040.00	Active efficiency	BAU	Biofuel increase	45.31
112	2050.00	Active efficiency	BAU	Biofuel increase	44.38
113	1920.00	BAU	Active renovation	Biofuel increase	139.77
114	1930.00	BAU	Active renovation	Biofuel increase	179.32
115	1940.00	BAU	Active renovation	Biofuel increase	222.64
116	1950.00	BAU	Active renovation	Biofuel increase	304.28
117	1960.00	BAU	Active renovation	Biofuel increase	357.78
118	1970.00	BAU	Active renovation	Biofuel increase	321.03
119	1980.00	BAU	Active renovation	Biofuel increase	134.56

120	1990.00	BAU	Active renovation	Biofuel increase	76.35
121	2000.00	BAU	Active renovation	Biofuel increase	50.07
122	2010.00	BAU	Active renovation	Biofuel increase	51.08
123	2020.00	BAU	Active renovation	Biofuel increase	46.89
124	2030.00	BAU	Active renovation	Biofuel increase	44.26
125	2040.00	BAU	Active renovation	Biofuel increase	42.73
126	2050.00	BAU	Active renovation	Biofuel increase	42.09
127	1920.00	Active efficiency	Active renovation	Biofuel increase	139.77
128	1930.00	Active efficiency	Active renovation	Biofuel increase	179.32
129	1940.00	Active efficiency	Active renovation	Biofuel increase	222.64
130	1950.00	Active efficiency	Active renovation	Biofuel increase	304.28
131	1960.00	Active efficiency	Active renovation	Biofuel increase	357.78
132	1970.00	Active efficiency	Active renovation	Biofuel increase	321.03
133	1980.00	Active efficiency	Active renovation	Biofuel increase	134.56
134	1990.00	Active efficiency	Active renovation	Biofuel increase	76.35
135	2000.00	Active efficiency	Active renovation	Biofuel increase	50.07
136	2010.00	Active efficiency	Active renovation	Biofuel increase	51.08
137	2020.00	Active efficiency	Active renovation	Biofuel increase	46.81
138	2030.00	Active efficiency	Active renovation	Biofuel increase	44.02
139	2040.00	Active efficiency	Active renovation	Biofuel increase	42.38
140	2050.00	Active efficiency	Active renovation	Biofuel increase	41.77
141	1920.00	BAU	Effective renovation	Biofuel increase	139.77
142	1930.00	BAU	Effective renovation	Biofuel increase	179.32
143	1940.00	BAU	Effective renovation	Biofuel increase	222.64
144	1950.00	BAU	Effective renovation	Biofuel increase	304.28
145	1960.00	BAU	Effective renovation	Biofuel increase	357.78
146	1970.00	BAU	Effective renovation	Biofuel increase	321.03
147	1980.00	BAU	Effective renovation	Biofuel increase	134.56
148	1990.00	BAU	Effective renovation	Biofuel increase	76.35
149	2000.00	BAU	Effective renovation	Biofuel increase	50.07
150	2010.00	BAU	Effective renovation	Biofuel increase	51.08
151	2020.00	BAU	Effective renovation	Biofuel increase	44.43
152	2030.00	BAU	Effective renovation	Biofuel increase	38.93
153	2040.00	BAU	Effective renovation	Biofuel increase	34.61
154	2050.00	BAU	Effective renovation	Biofuel increase	31.53
155	1920.00	Active efficiency	Effective renovation	Biofuel increase	139.77
156	1930.00	Active efficiency	Effective renovation	Biofuel increase	179.32
157	1940.00	Active efficiency	Effective renovation	Biofuel increase	222.64
158	1950.00	Active efficiency	Effective renovation	Biofuel increase	304.28
159	1960.00	Active efficiency	Effective renovation	Biofuel increase	357.78
160	1970.00	Active efficiency	Effective renovation	Biofuel increase	321.03
161	1980.00	Active efficiency	Effective renovation	Biofuel increase	134.56
162	1990.00	Active efficiency	Effective renovation	Biofuel increase	76.35
163	2000.00	Active efficiency	Effective renovation	Biofuel increase	50.07
164	2010.00	Active efficiency	Effective renovation	Biofuel increase	51.08
165	2020.00	Active efficiency	Effective renovation	Biofuel increase	44.35
166	2030.00	Active efficiency	Effective renovation	Biofuel increase	38.69
167	2040.00	Active efficiency	Effective renovation	Biofuel increase	34.26
168	2050.00	Active efficiency	Effective renovation	Biofuel increase	31.22

```
> ggplot(subset(dal, FuelPolicy == "BAU"), aes(x = Time, weight = DALYsResult, fill = Heating))+geom_bar(binwidth = 5) +
+ facet_grid(EfficiencyPolicy ~ RenovationPolicy) +
+ theme_gray(base_size = BS) +
+ labs(
+ title = "Health effects in DALYs of PM2.5 from heating in Kuopio",
+ x = "Time",
+ y = "Health effects (DALY /a)"
+ )
> ggplot(subset(dal, Time == 2030), aes(x = RenovationPolicy, weight = DALYsResult, fill = Heating))+geom_bar() +
+ facet_grid(EfficiencyPolicy ~ FuelPolicy) +
+ theme_gray(base_size = BS) +
+ labs(
```

```
+ title = "Health effects in DALYs of PM2.5 from heating in Kuopio 2030",
+ x = "Biofuel policy in district heating",
+ y = "Health effects (DALY /a)"
+ )
> ######## Buildings in Kuopio on map
> if(FALSE){
+ # Calculate locations for Kuopio districts
+
+ temp <- buildings
+ temp@output <- subset(temp@output,
+ Time == 2030 & EfficiencyPolicy == "BAU" & RenovationPolicy == "BAU"
+ )
+ temp <- unkeep(temp, sources = TRUE, prevresults = TRUE)
+ temp <- oapply(temp, cols = c("Building", "Heating", "Efficiency", "Renovation"), FUN = sum)
+
+ ####!------------------------------------------------
+ districts <- tidy(opbase.data("Op_en5932.kuopio_city_districts"), widecol = "Location") # [[Building stock in Kuopio]]
+ ####i------------------------------------------------
+
+ colnames(districts) <- gsub("[ \\.]", "_", colnames(districts))
+ districts <- Ovariable("districts", data = data.frame(districts, Result = 1))
+
+ temp <- temp * districts
+
+ MyRmap(
+ ova2spat(
+ temp,
+ coord = c("E", "N"),
+ proj4string = "+init=epsg:3067"
+ ), # National Land Survey uses EPSG:3067 (ETRS-TM35FIN)
+ plotvar = "Result",
+ legend_title = "Floor area",
+ numbins = 8,
+ pch = 19,
+ cex = 2
+ )
+ }
> if(saveobjects) {
+ objects.put(list = ls())
+ cat(c("All objects archived. Write down the key of the run to retrieve them with objects.get. Objects: ",
+ ls(), "\n"))
+ }
All objects archived. Write down the key of the run to retrieve them with objects.get. Objects: ana2ova bgexposure BS bui buildings buildingTypes BW changeBuildings collapsemarg construction constructionAreas dal DALYs DecefficiencyShares DecfuelShares decisions DecrenovationRate DecrenovationShares disincidence dose dummy DW efficiencyRatio efficiencyShares emis emissionFactors emissionLocations emissions energyFactor energyUse ERF ERF_diox ERF_env ERF_mehg ERF_omega3 exposure figstofile findrest finnish forgetDecisions frexposed fuelShares fuelSharesgeneric fuelUse heatingShares iF L makeTimeline MyPlotKML MyPointKML MyRmap obstime ograph orbind2 ova2spat population renovationRate renovationRatio renovationShares RR saveobjects server stockBuildings suomenna testforrow threshold threshold_diox threshold_env threshold_mehg threshold_omega3 timepoints timing totcases truncateIndex wiki_username year
```

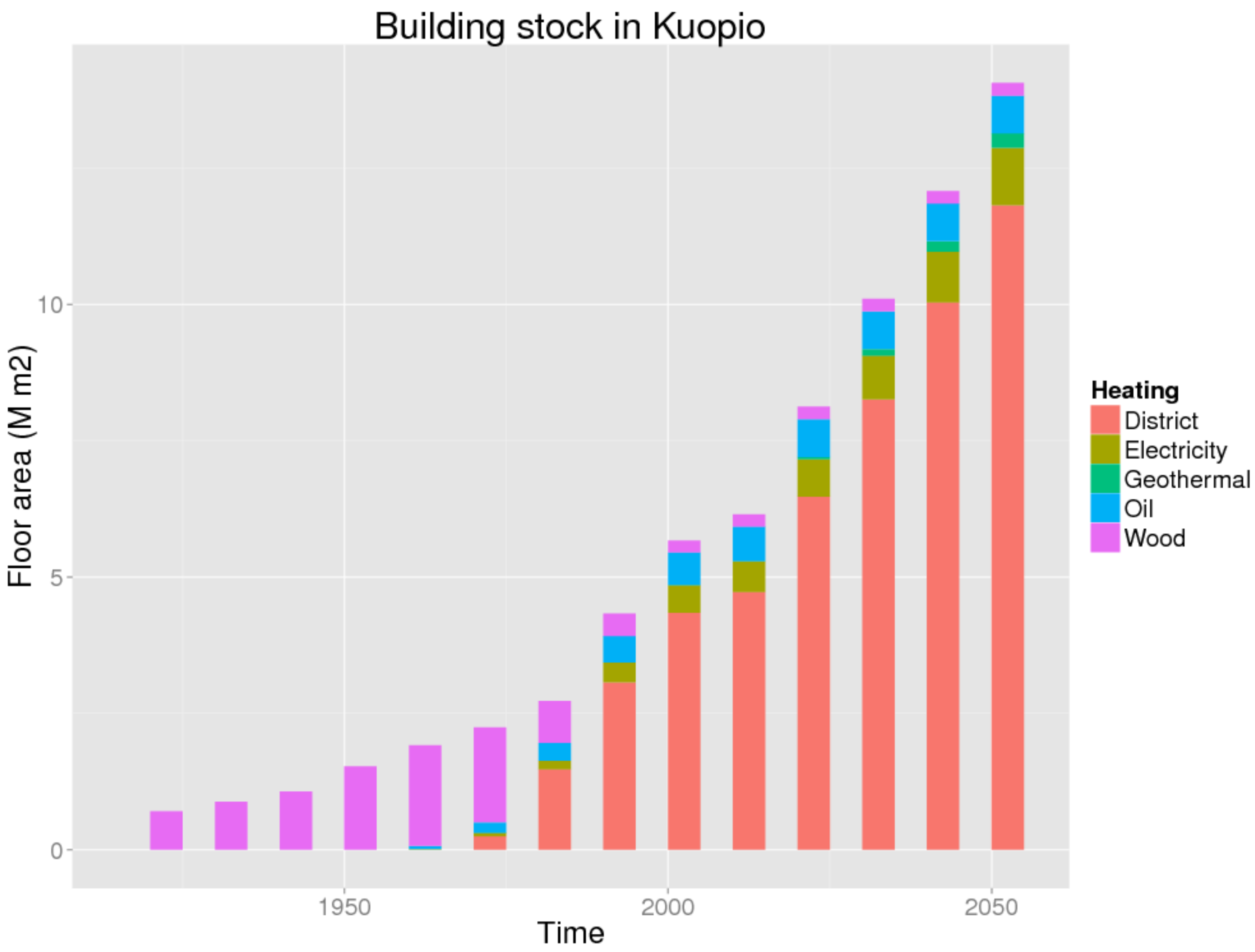
Building stock in Kuopio
Floor area (M m2)
0
5
10
1950
2000
2050
Time
Heating
District
Electricity
Geothermal
Oil
Wood

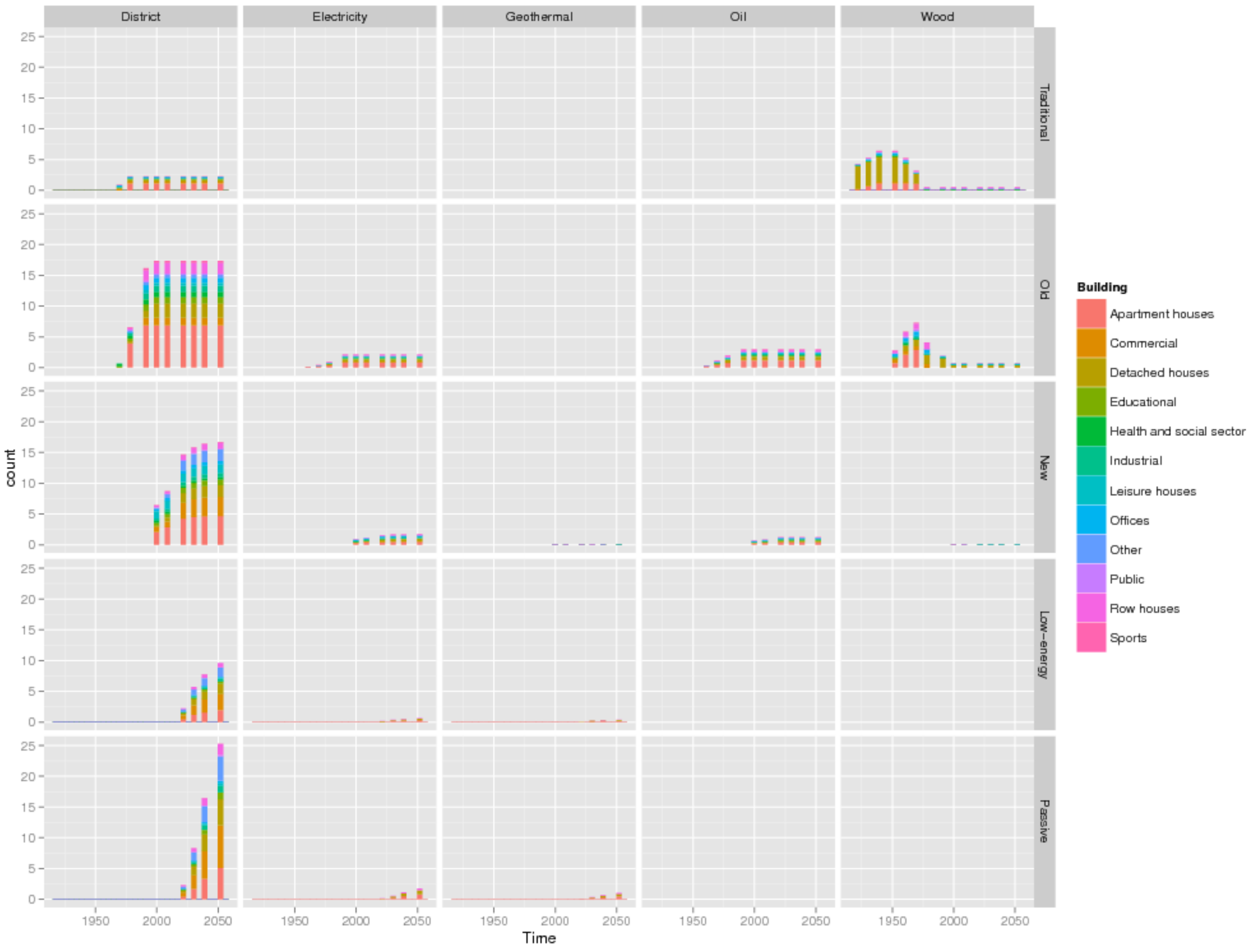
District
Electricity
Geothermal
Oil
Wood
Traditional
Old
New
Low−energy
Passive
count
Time
Building
Apartment houses
Commercial
Detached houses
Educational
Health and social sector
Industrial
Leisure houses
Offices
Other
Public
Row houses
Sports

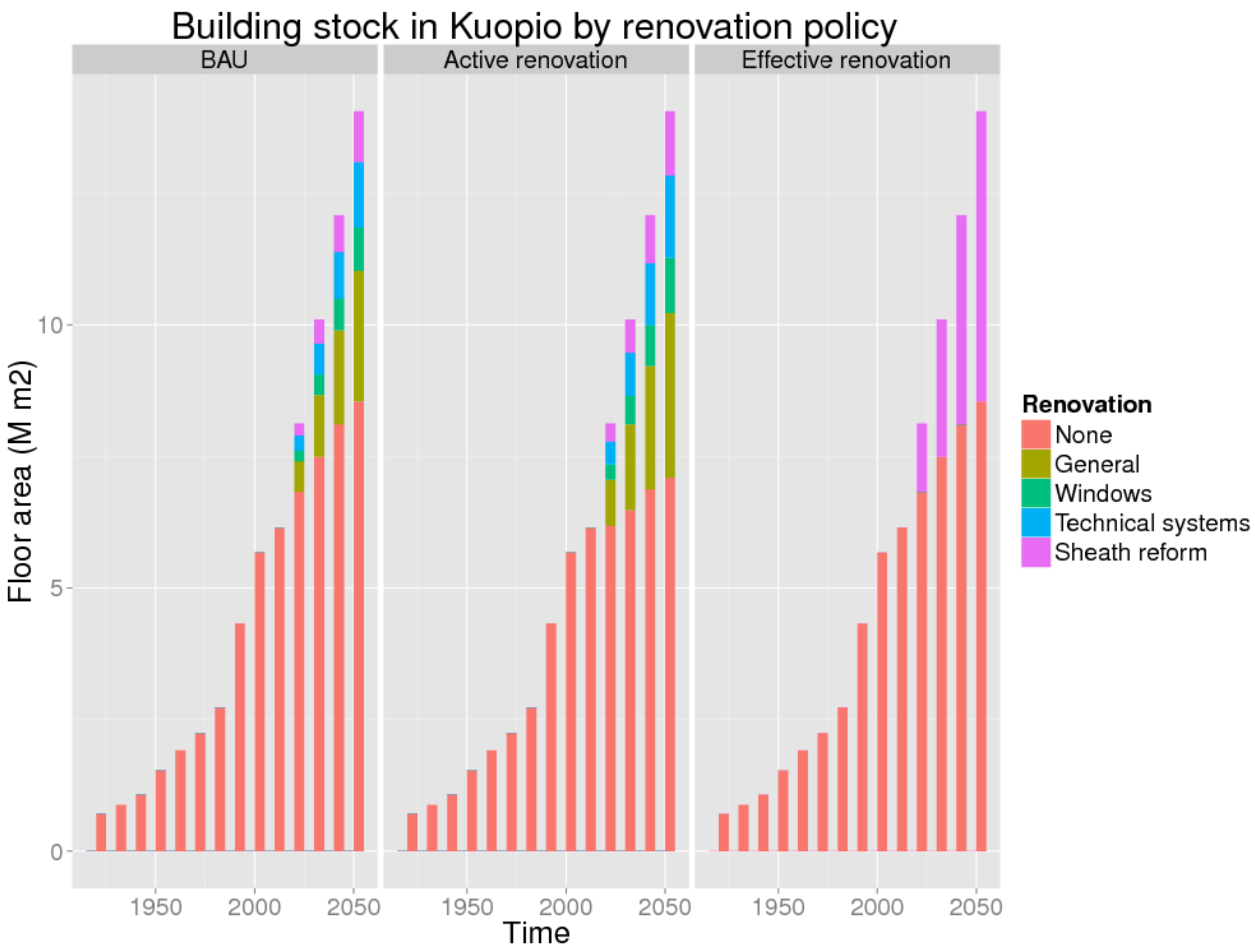
Building stock in Kuopio by renovation policy
BAU
Active renovation
Effective renovation
Floor area (M m2)
0
5
10
1950
2000
2050
Time
Renovation
None
General
Windows
Technical systems
Sheath reform

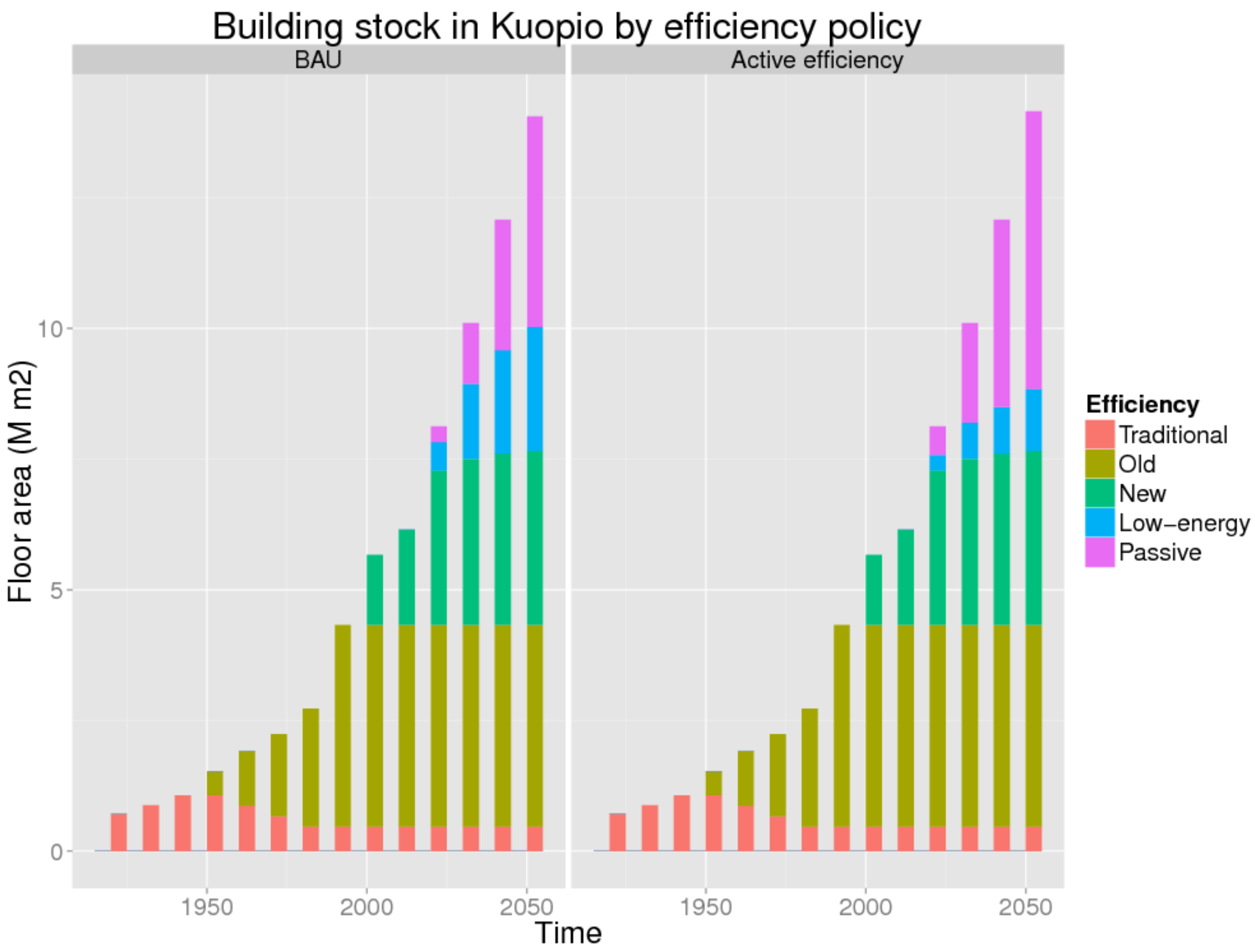
Building stock in Kuopio by efficiency policy
BAU
Active efficiency
Floor area (M m2)
10
5
0
1950
2000
2050
Time
Efficiency
Traditional
Old
New
Low−energy
Passive

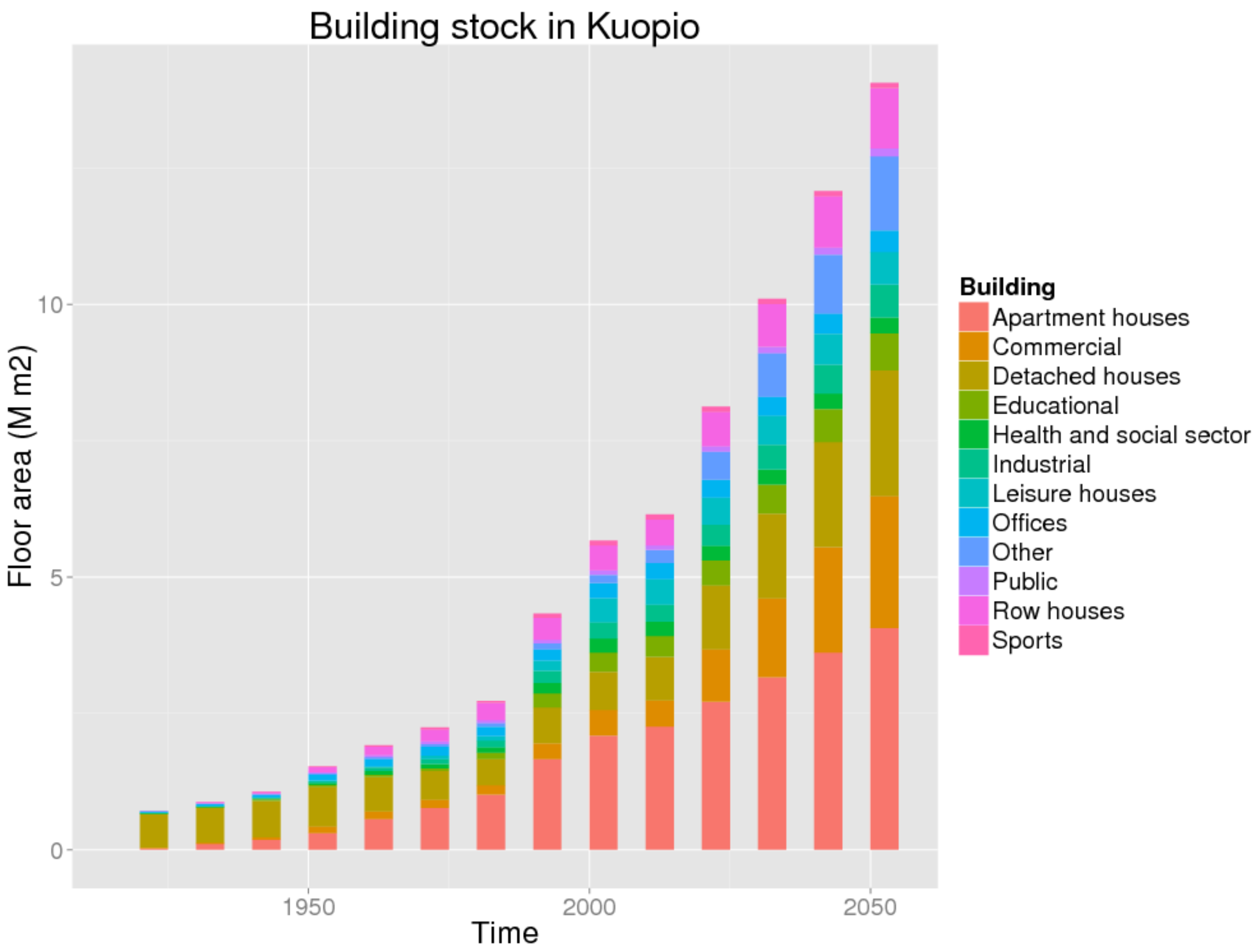
Building stock in Kuopio
Floor area (M m2)
0
5
10
1950
2000
2050
Time
Building
Apartment houses
Commercial
Detached houses
Educational
Health and social sector
Industrial
Leisure houses
Offices
Other
Public
Row houses
Sports

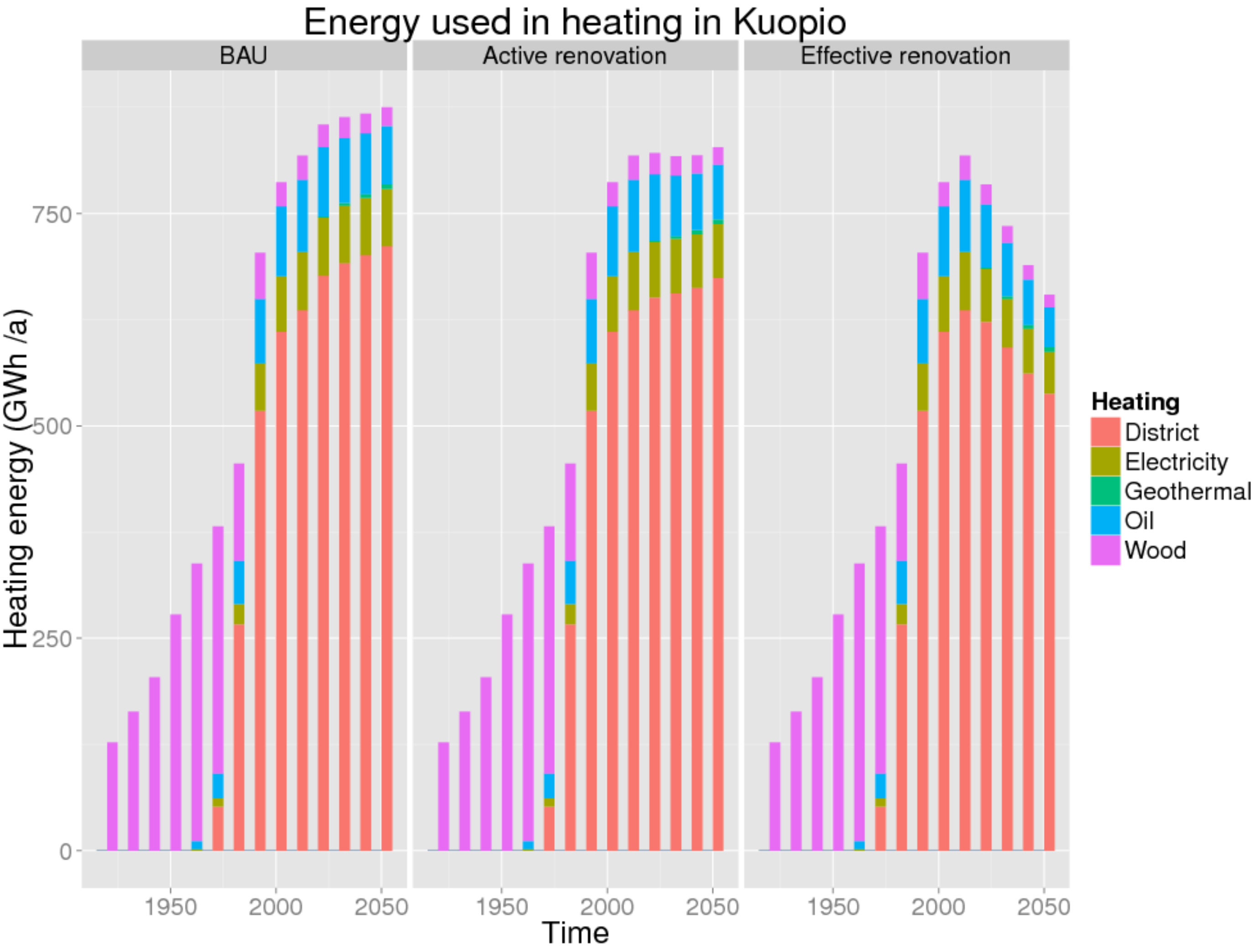
Energy used in heating in Kuopio
BAU
Active renovation
Effective renovation
Heating energy (GWh /a)
0
250
500
750
1950
2000
2050
Time
Heating
District
Electricity
Geothermal
Oil
Wood

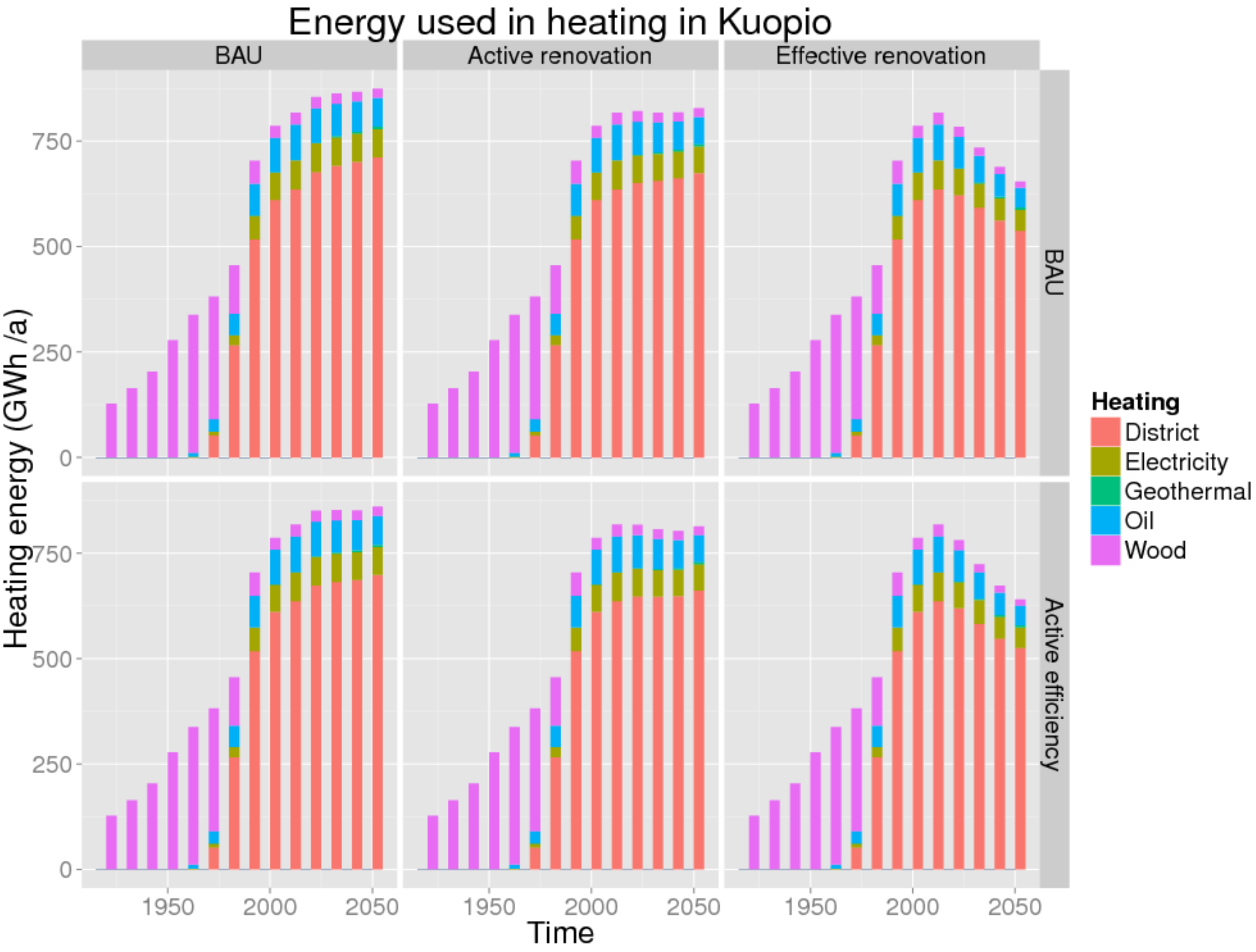
Energy used in heating in Kuopio
BAU
Active renovation
Effective renovation
BAU
Active efficiency
Heating energy (GWh /a)
0
250
500
750
1950
2000
2050
Time
Heating
District
Electricity
Geothermal
Oil
Wood

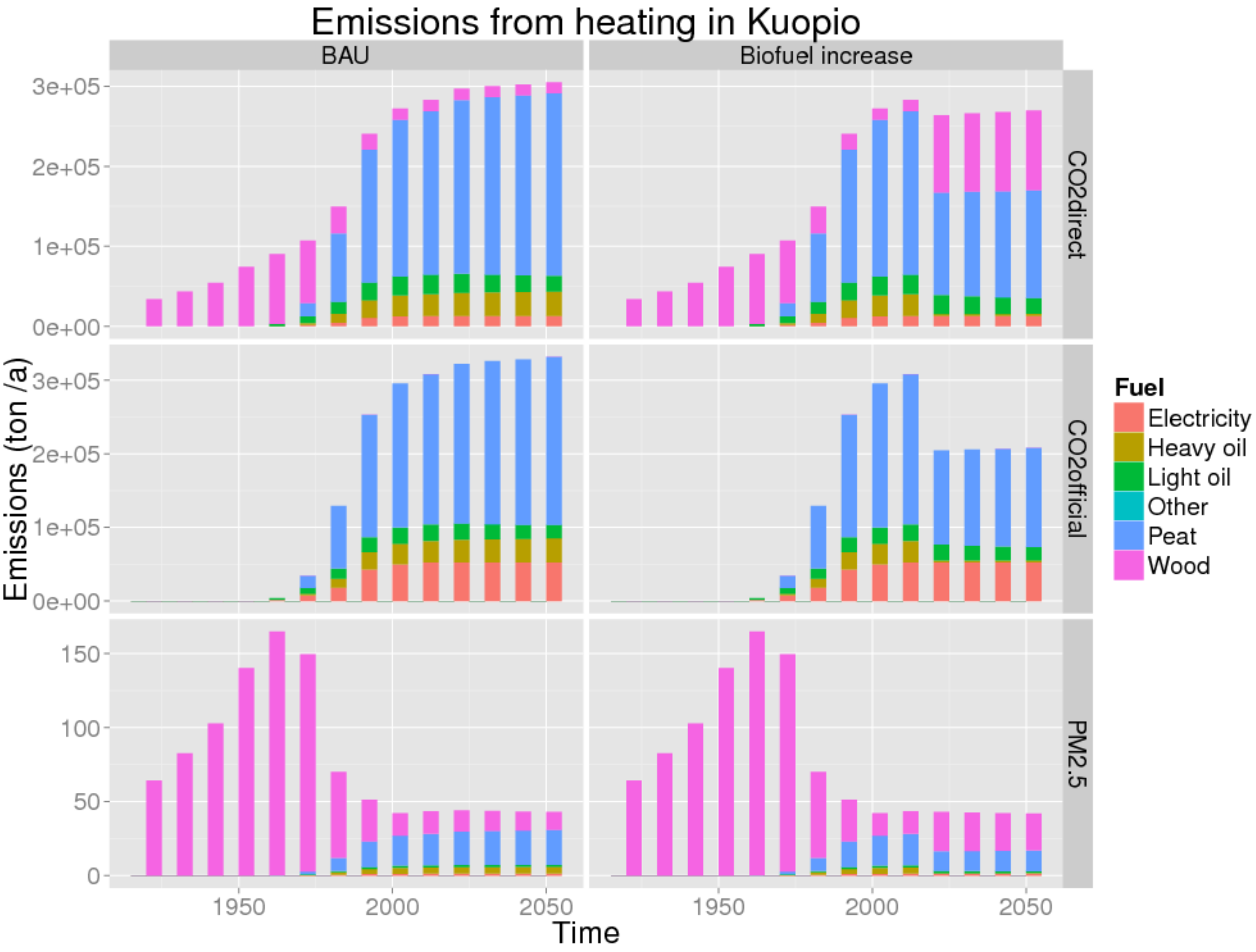
Emissions from heating in Kuopio
BAU
Biofuel increase
CO2direct
CO2official
PM2.5
Emissions (ton /a)
Time
3e+05
2e+05
1e+05
0e+00
150
100
50
0
1950
2000
2050
Fuel
Electricity
Heavy oil
Light oil
Other
Peat
Wood

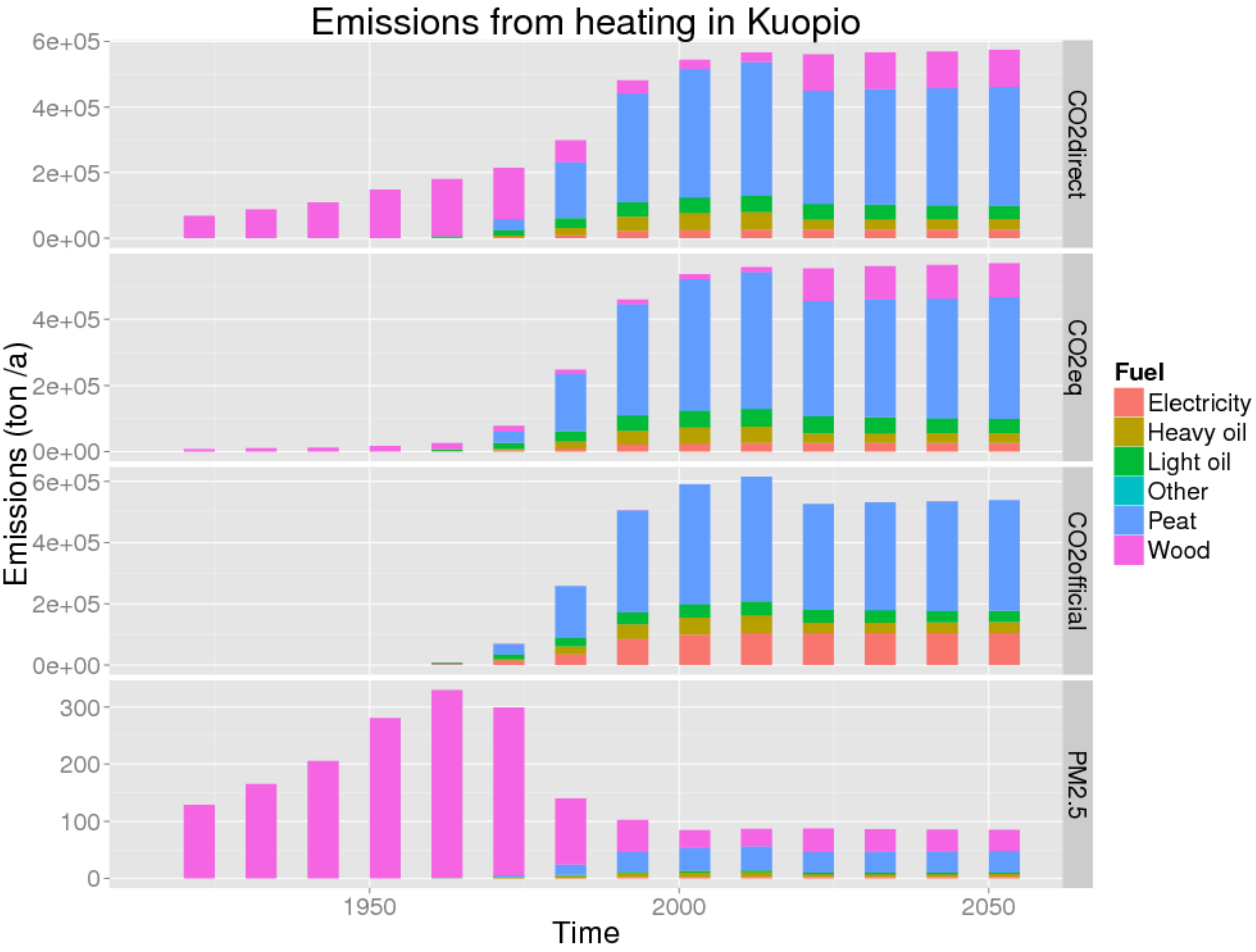
Emissions from heating in Kuopio
Emissions (ton /a)
6e+05
4e+05
2e+05
0e+00
CO2direct
CO2eq
CO2official
PM2.5
300
200
100
0
1950
2000
2050
Time
Fuel
Electricity
Heavy oil
Light oil
Other
Peat
Wood

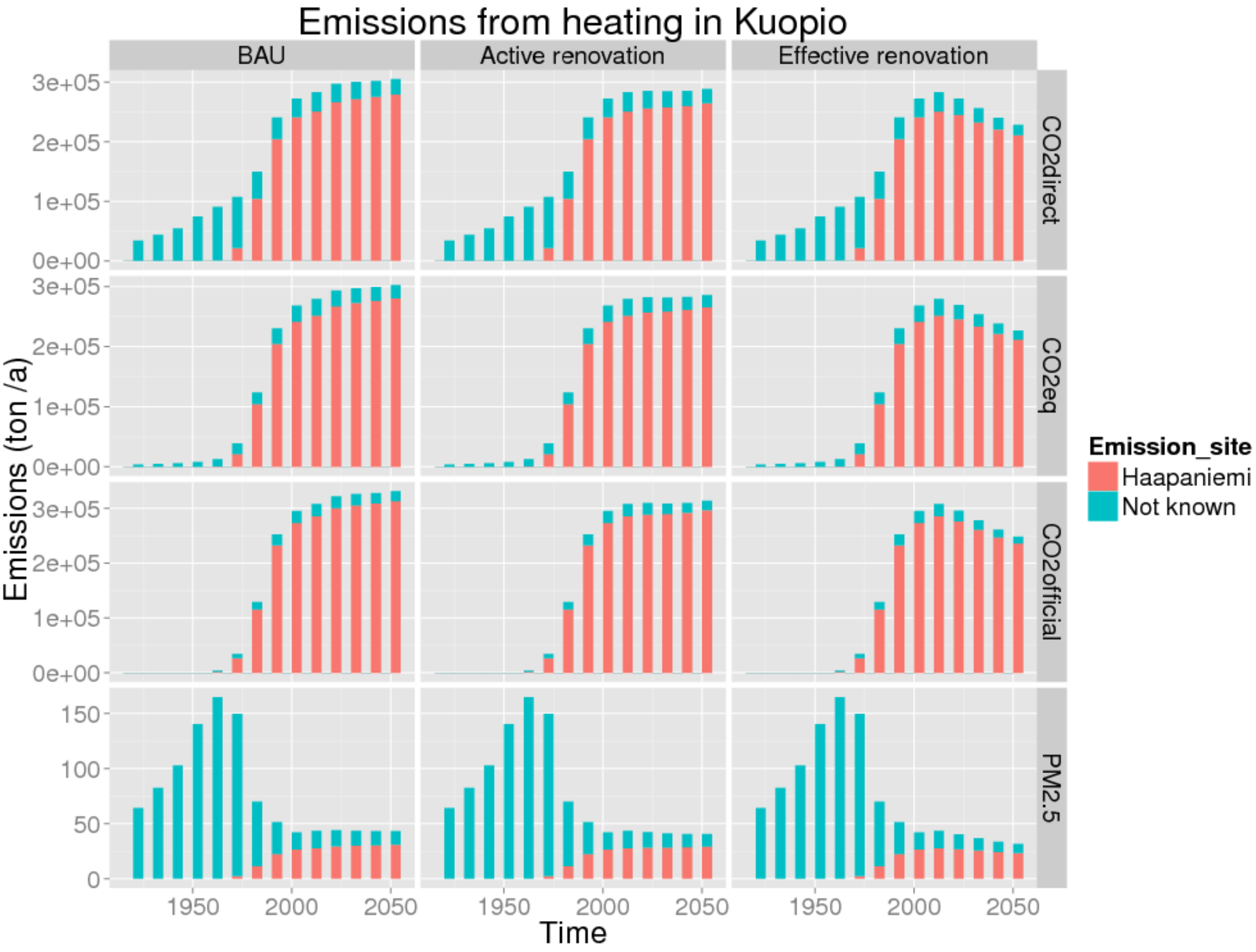
Emissions from heating in Kuopio
BAU
Active renovation
Effective renovation
CO2direct
CO2eq
CO2official
PM2.5
Emissions (ton /a)
Time
3e+05
2e+05
1e+05
0e+00
150
100
50
0
1950
2000
2050
Emission_site
Haapaniemi
Not known

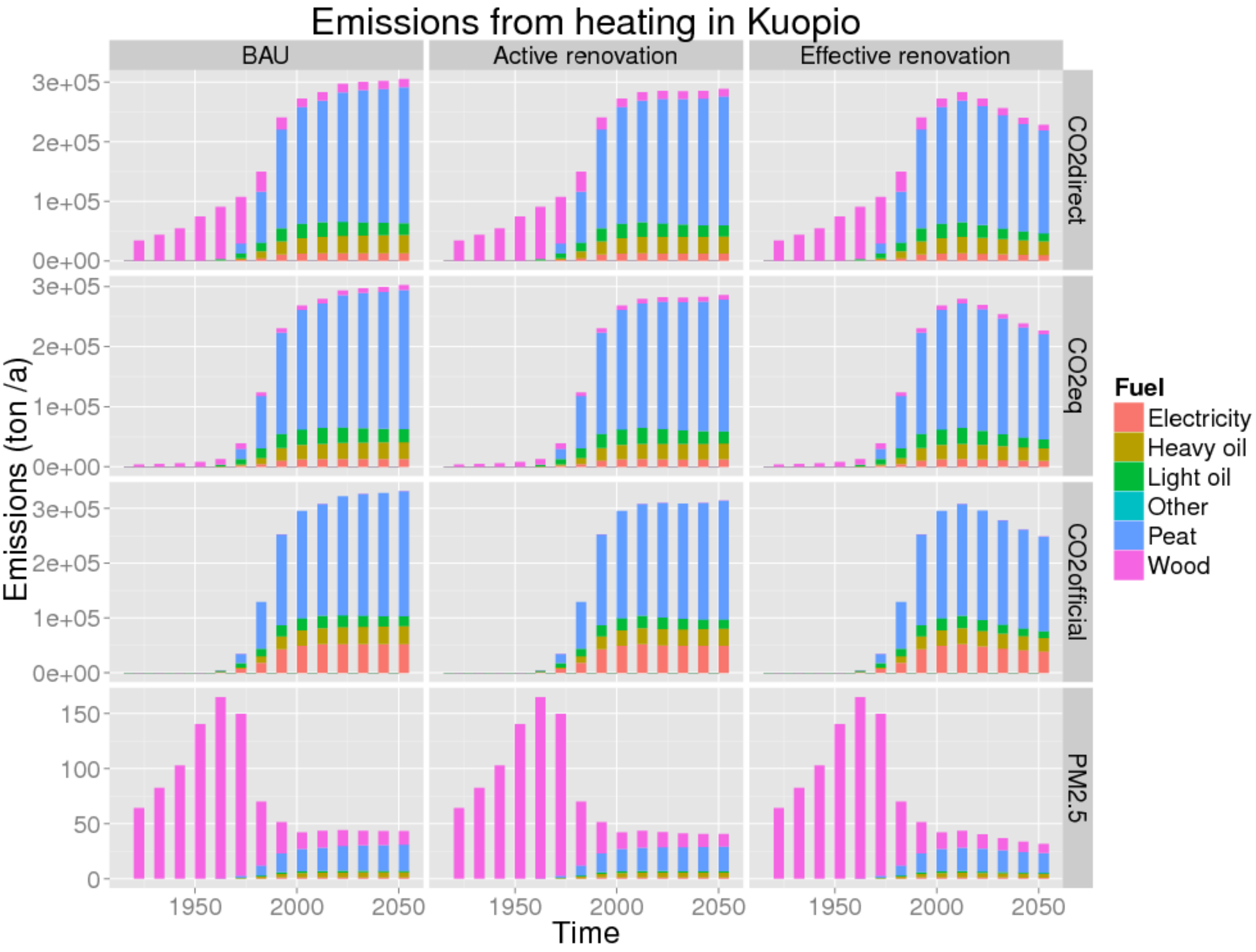
Emissions from heating in Kuopio
BAU
Active renovation
Effective renovation
CO2direct
CO2eq
CO2official
PM2.5
Emissions (ton /a)
3e+05
2e+05
1e+05
0e+00
150
100
50
0
1950
2000
2050
Time
Fuel
Electricity
Heavy oil
Light oil
Other
Peat
Wood

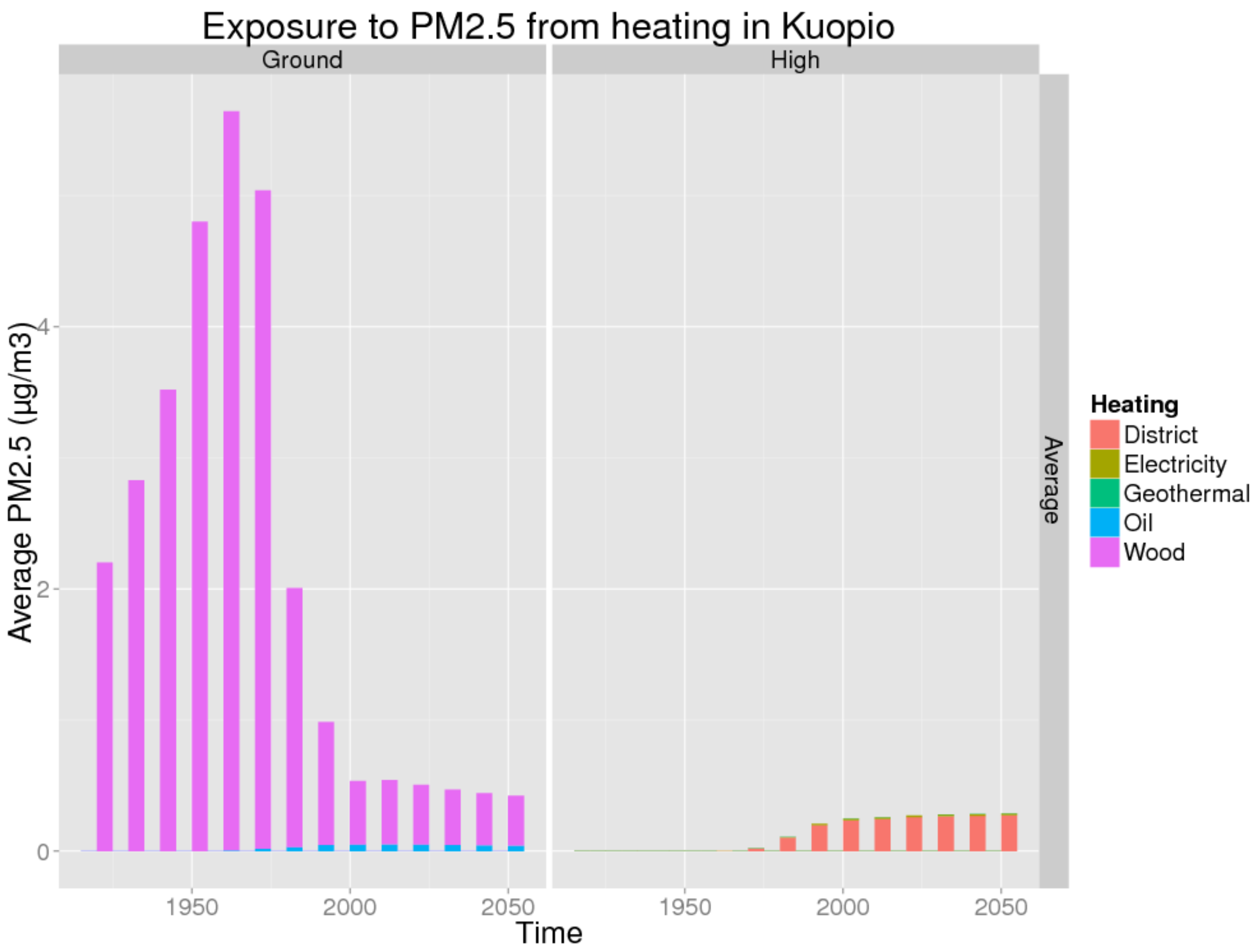
Exposure to PM2.5 from heating in Kuopio
Ground
High
Average
Average PM2.5 (µg/m3)
0
2
4
1950
2000
2050
1950
2000
2050
Time
Heating
District
Electricity
Geothermal
Oil
Wood

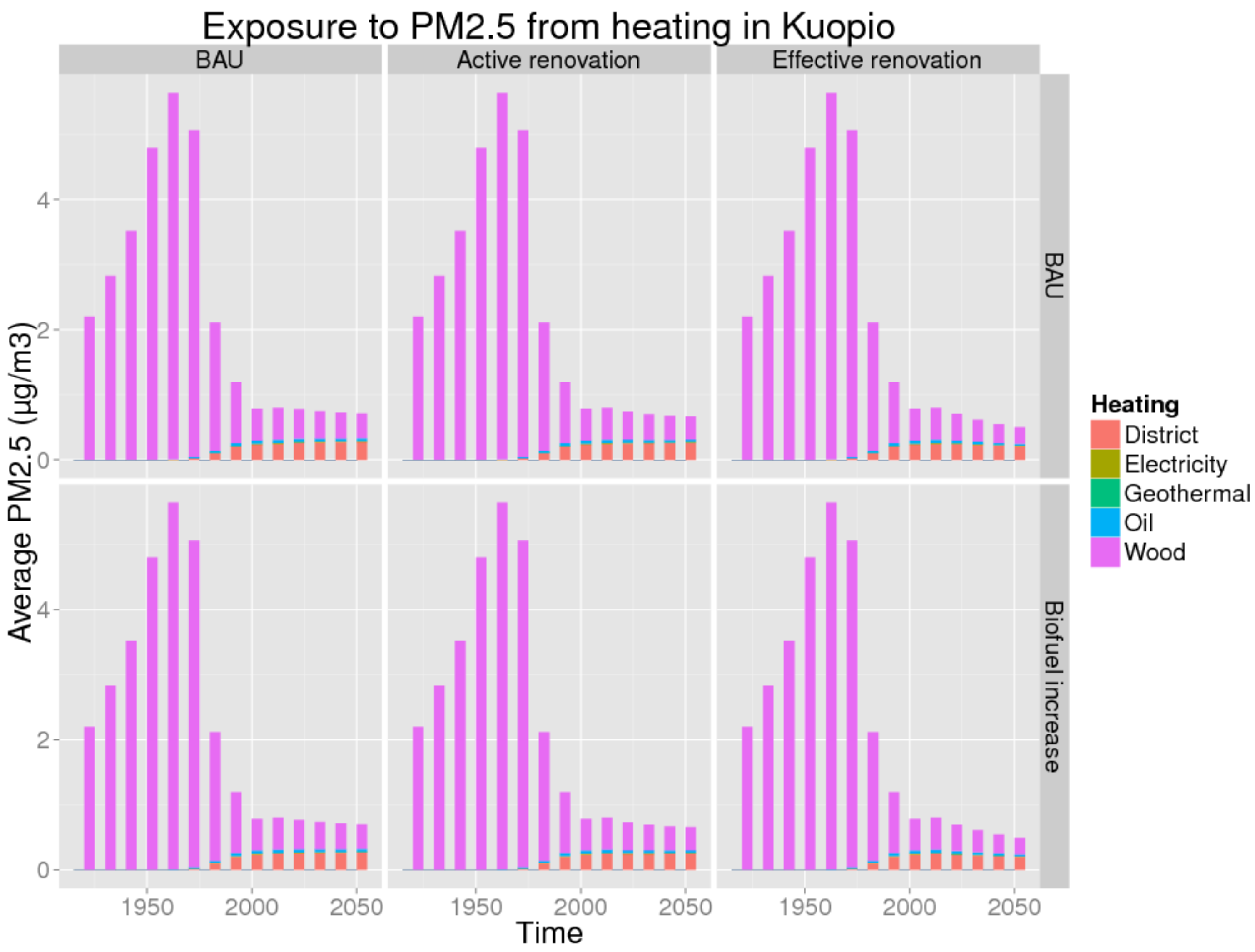
Exposure to PM2.5 from heating in Kuopio
BAU
Active renovation
Effective renovation
BAU
Biofuel increase
Average PM2.5 (µg/m3)
0
2
4
1950
2000
2050
Time
Heating
District
Electricity
Geothermal
Oil
Wood

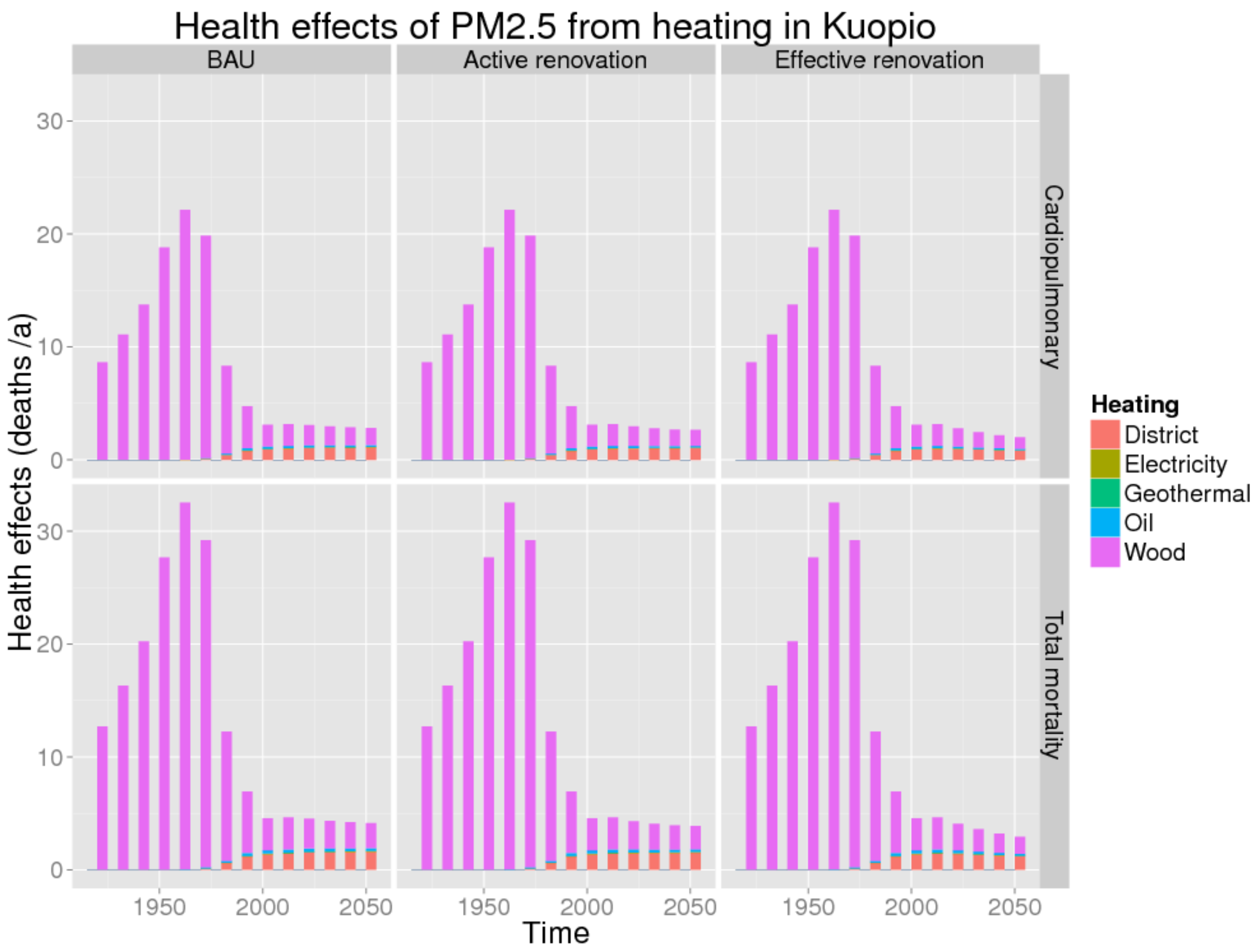
Health effects of PM2.5 from heating in Kuopio
BAU
Active renovation
Effective renovation
Cardiopulmonary
Total mortality
Health effects (deaths /a)
0
10
20
30
1950
2000
2050
Time
Heating
District
Electricity
Geothermal
Oil
Wood

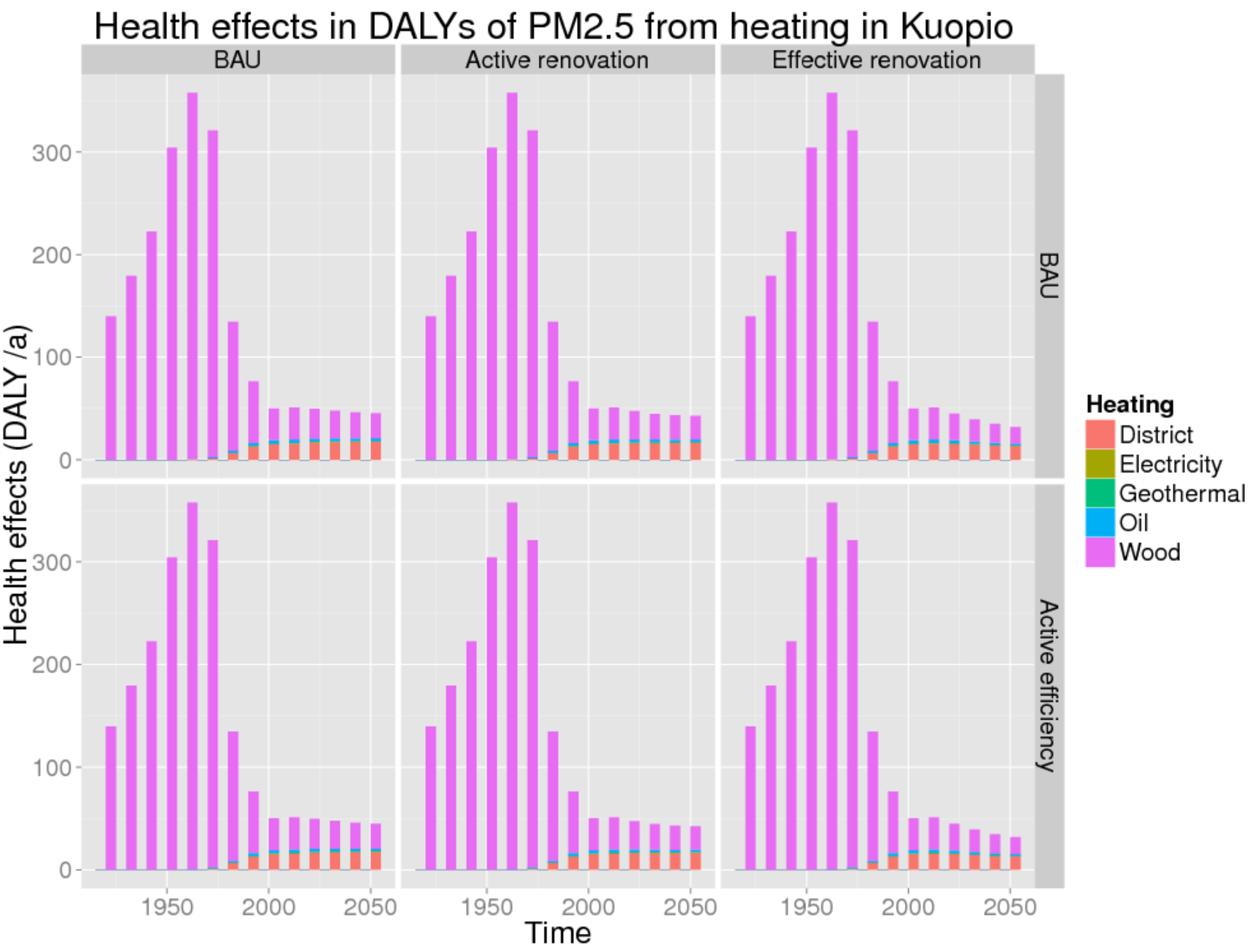
Health effects in DALYs of PM2.5 from heating in Kuopio
BAU
Active renovation
Effective renovation
BAU
Active efficiency
Health effects (DALY /a)
0
100
200
300
1950
2000
2050
Time
Heating
District
Electricity
Geothermal
Oil
Wood

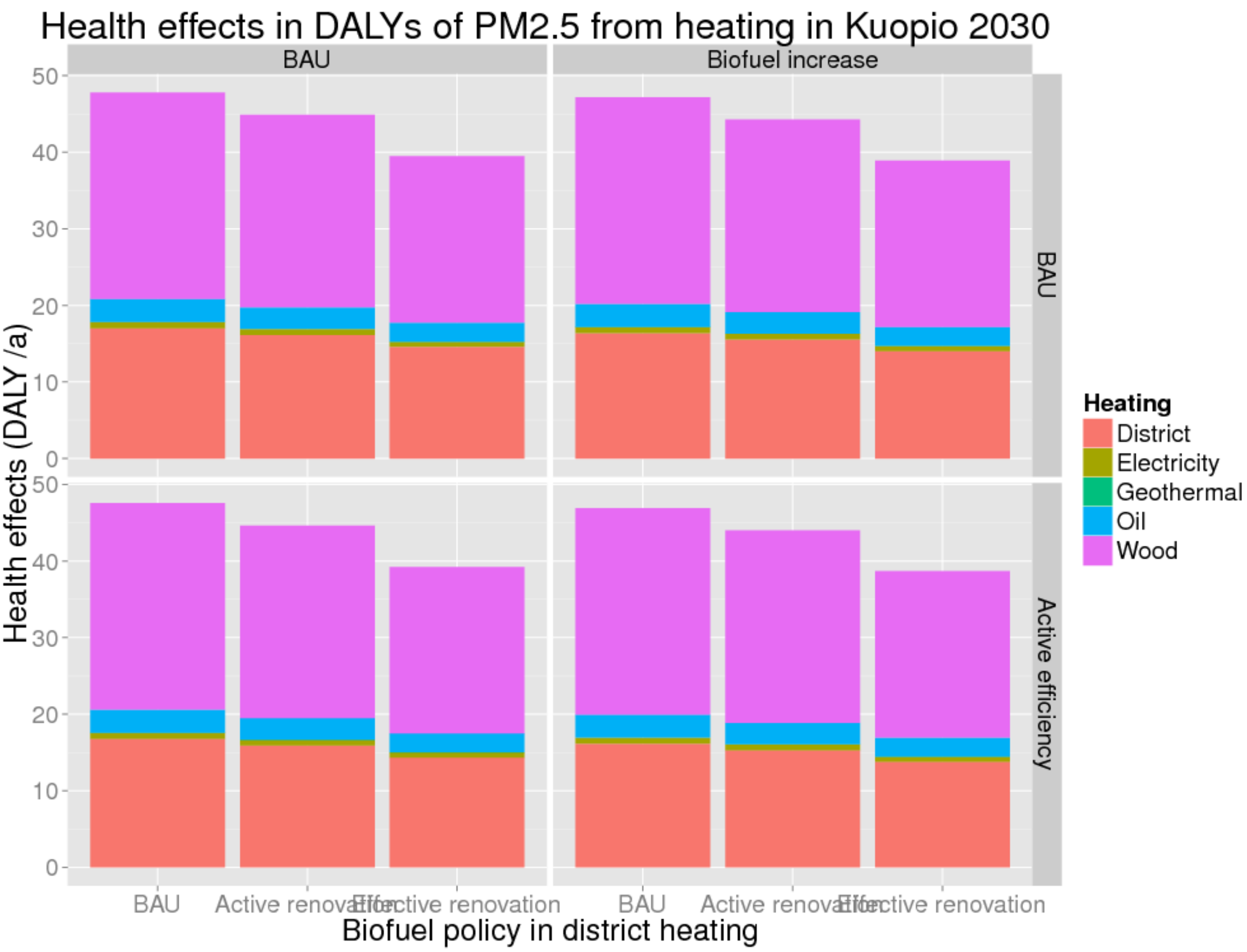
Health effects in DALYs of PM2.5 from heating in Kuopio 2030
BAU
Biofuel increase
BAU
Active efficiency
Health effects (DALY /a)
0
10
20
30
40
50
BAU
Active renovation
Effective renovation
Biofuel policy in district heating
Heating
District
Electricity
Geothermal
Oil
Wood
