# Supplementary material for: Building-related health impacts in European and Chinese cities: a scalable assessment method
Source: Environ Health. 2015 Dec 14;14:93. doi: 10.1186/s12940-015-0082-z (PMC4678713; doi:10.1186/s12940-015-0082-z)
Supplement: Additional file 3: — Model run for Basel. R-model run for Basel case with the R-code and the results. (PDF 1558 kb) [file 12940_2015_82_MOESM3_ESM.pdf]

R-tools

Ran at 2015-07-24 05:22:22

```
> wiki_username <- "Jouni"
> library(OpasnetUtils)
> library(ggplot2)
> library(maptools)
> library(RColorBrewer)
> library(classInt)
> #library(OpasnetUtilsExt)
> library(RgoogleMaps)
> ### THIS CODE IS FROM PAGE [[Climate change policies in Basel]] (Op_en5480, code_name = "")
> ########### TECHNICAL PARAMETERS
> openv.setN(0) # use medians instead of whole sampled distributions
> objects.latest("Op_en6007", code_name = "answer") # [[OpasnetUtils/Drafts]] findrest
> obstime <- Ovariable("obstime", data = data.frame(Obsyear = factor(seq(2010, 2050, 10), ordered = TRUE), Result = 1))
> BS <- 24 # base_size for graph font
> figstofile <- FALSE
> saveobjects <- TRUE
> finnish <- FALSE
> suomenna <- function(ova) {
+ if(class(ova) == "ovariable") out <- ova@output else out <- ova
+ if("Heating" %in% colnames(out)) {
+ out$Heating <- as.factor(out$Heating)
+ levels(out$Heating)[levels(out$Heating) == "Long-distance heating"] <- "District"
+ }
+ if("Response" %in% colnames(out)) {
+ out$Response <- as.factor(out$Response)
+ levels(out$Response)[levels(out$Response) == "Cardiopulmonary mortality"] <- "Cardiopulmonary"
+ }
+ out$Time <- as.numeric(as.character(out$Time))
+ return(out)
+ }
> ##################### Decisions
> decisions <- opbase.data('Op_en5480') # [[Climate change policies in Basel]]
> DecisionTableParser(decisions)
> # Remove previous decisions, if any.
> forgetDecisions <- function() {
+ for(i in ls(envir = openv)) {
+ if("dec_check" %in% names(openv[[i]])) openv[[i]]$dec_check <- FALSE
+ }
+ return(cat("Decisions were forgotten.\n"))
+ }
> forgetDecisions()
Decisions were forgotten.

> ############################ Submodels and data
> objects.latest("Op_en7044", code_name = "initiate") # [[Buildings in Basel]]
> # Contains stockBuildings, changeBuildings, emissionLocations, heatingShares, renovationRate, renovationShares
> objects.latest("Op_en6289", code_name = "buildingstest") # [[Building model]] # Generic building model.
> ### Energy and emissions
> objects.latest("Op_en5488", code_name = "energyUseAnnual") # [[Energy use of buildings]] energyUse
> objects.latest("Op_en5488", code_name = "efficiencyShares") # [[Energy use of buildings]]
> objects.latest("Op_en2791", code_name = "emissionstest") # [[Emission factors for burning processes]] emissions
> objects.latest("Op_en2791", code_name = "emissionFactors") # [[Emission factors for burning processes]]
> objects.latest("Op_en7044", code_name = "fuelShares") # [[Buildings in Basel]]
> objects.latest("Op_en5141", code_name = "fuelUse") # [[Energy balance]]
> ## Exposure and health assessment
> objects.latest("Op_en5813", code_name = "exposure") # [[Intake fractions of PM]] uses Humbert iF as default.
> objects.latest('Op_en2261', code_name = 'totcases') # [[Health impact assessment]] totcases and dependencies.
> objects.latest('Op_en5461', code_name = 'DALYs') # [[Climate change policies and health in Kuopio]] DALYs, DW, L
> population <- 192000 # Contains only the Basel city, i.e. assumes no exposure outside city.
> # population <- 700000 # Contains the Basel metropolitan area, as that is exposed to PM2.5.
> # Note: the population size does NOT affect the health impact as it cancels out. However, it DOES affect
> # exposure estimates.
> ###################### MODEL CALCULATIONS
> renovationRate <- EvalOutput(renovationRate) * 10 # Rates for 10-year periods
> renovationShares <- EvalOutput(renovationShares)
> stockBuildings <- EvalOutput(stockBuildings)
> changeBuildings <- EvalOutput(changeBuildings)
> buildings <- EvalOutput(buildings)
[1] "Column RenovationPolicy treated with fillna (difference between buil and renovate)."

> buildings@output$RenovationPolicy <- factor(
+ buildings@output$RenovationPolicy,
+ levels = c("BAU", "Active renovation", "Total renovation"),
+ ordered = TRUE
+ )
> buildings@output$EfficiencyPolicy <- factor(
```

```
+ buildings@output$EfficiencyPolicy,
+ levels = c("BAU", "Active efficiency"),
+ ordered = TRUE
+ )
> energyUse <- EvalOutput(energyUse)
> fuelUse <- EvalOutput(fuelUse)
> fuelUse <- fuelUse * 1E-3 *3600 # kWh -> MJ
> emissions <- EvalOutput(emissions)
> emissions@output$Time <- as.numeric(as.character(emissions@output$Time))
> exposure <- EvalOutput(exposure)
> exposure@output <- exposure@output[exposure@output$Area == "Urban" , ] # Basel is an urban area,
> # rather than rural or average.
> totcases <- EvalOutput(totcases)
> totcases@output$Time <- as.numeric(as.character(totcases@output$Time))
> totcases <- oapply(totcases, cols = c("Age", "Sex"), FUN = sum)
> DALYs <- EvalOutput(DALYs)
> ############## OUTPUT GRAPHS AND TABLES
> bui <- oapply(buildings * 1E-6, cols = c("City_area", "buildingsSource"), FUN = sum)
> bui <- suomenna(truncateIndex(bui, cols = "Heating", bins = 3))
> ggplot(subset(bui, EfficiencyPolicy == "BAU" & RenovationPolicy == "BAU"), aes(x = Time, weight = buildingsResult, fill = Heating)) +
geom_bar(binwidth = 5) +
+ theme_gray(base_size = BS) +
+ labs(
+ title = "Building stock in Basel by heating",
+ x = "Time",
+ y = "Floor area (M m2)"
+ )
> if(figstofile) ggsave("Figure6.eps", width = 8, height = 7)
>
> ggplot(subset(bui, EfficiencyPolicy == "BAU"), aes(x = Time, weight = buildingsResult, fill = Renovation)) + geom_bar(binwidth = 5) +
+ facet_grid(. ~ RenovationPolicy) + theme_gray(base_size = BS) +
+ labs(
+ title = "Building stock in Basel by renovation policy",
+ x = "Time",
+ y = "Floor area (M m2)"
+ )
> ggplot(subset(bui, RenovationPolicy == "BAU"), aes(x = Time, weight = buildingsResult, fill = Efficiency)) + geom_bar(binwidth = 5) +
+ facet_grid(. ~ EfficiencyPolicy) + theme_gray(base_size = BS) +
+ labs(
+ title = "Building stock in Basel by efficiency policy",
+ x = "Time",
+ y = "Floor area (M m2)"
+ )
> ggplot(subset(bui, RenovationPolicy == "BAU" & EfficiencyPolicy == "BAU"), aes(x = Time, weight = buildingsResult, fill = Heating)) +
geom_bar(binwidth = 5) +
+ theme_gray(base_size = BS) +
+ labs(
+ title = "Building stock in Basel",
+ x = "Time",
+ y = "Floor area (M m2)"
+ )
> ggplot(subset(bui, RenovationPolicy == "BAU" & EfficiencyPolicy == "BAU"), aes(x = Time, weight = buildingsResult, fill = Building)) +
geom_bar(binwidth = 5) +
+ theme_gray(base_size = BS) +
+ labs(
+ title = "Building stock in Basel",
+ x = "Time",
+ y = "Floor area (M m2)"
+ )
> # Plot energy need and emissions
> hea <- oapply(energyUse * 1E-6, cols = c("City_area", "buildingsSource"), FUN = sum)
> hea <- suomenna(truncateIndex(hea, cols = "Heating", bins = 3))
> ggplot(hea, aes(x = Time, weight = energyUseResult, fill = Heating)) + geom_bar(binwidth = 5) +
+ facet_wrap( ~ RenovationPolicy) + theme_gray(base_size = BS) +
+ labs(
+ title = "Energy used in heating in Basel",
+ x = "Time",
+ y = "Heating energy (GWh /a)"
+ )
> emis <- suomenna(truncateIndex(emissions, cols = "Emission_site", bins = 5))
> ggplot(subset(emis, EfficiencyPolicy == "BAU" & FuelPolicy == "BAU"), aes(x = Time, weight = emissionsResult, fill = Emission_site)) +
geom_bar(binwidth = 5) +
+ facet_grid(Pollutant ~ RenovationPolicy, scale = "free_y") + theme_gray(base_size = BS) +
+ labs(
+ title = "Emissions from heating in Basel",
+ x = "Time",
+ y = "Emissions (ton /a)"
+ )
> ggplot(subset(emis, EfficiencyPolicy == "BAU" & RenovationPolicy == "BAU"), aes(x = Time, weight = emissionsResult, fill = Fuel)) +
geom_bar(binwidth = 5) +
+ facet_grid(Pollutant ~ FuelPolicy, scale = "free_y") + theme_gray(base_size = BS) +
+ labs(
```

```
+ title = "Emissions from heating in Basel",
+ x = "Time",
+ y = "Emissions (ton /a)"
+ )
> ggplot(subset(emis, EfficiencyPolicy == "BAU" & FuelPolicy == "BAU"), aes(x = Time, weight = emissionsResult, fill = Fuel)) + geom_bar(binwidth = 5)
+
+ facet_grid(Pollutant ~ RenovationPolicy, scale = "free_y") + theme_gray(base_size = BS) +
+ labs(
+ title = "Emissions from heating in Basel",
+ x = "Time",
+ y = "Emissions (ton /a)"
+ )
> expo <- suomenna(exposure)
> ggplot(subset(expo, RenovationPolicy == "BAU" & EfficiencyPolicy == "BAU" & FuelPolicy == "BAU"), aes(x = Time, weight = exposureResult, fill =
Heating)) + geom_bar(binwidth = 5) + facet_grid(Area ~ Emission_height) + theme_gray(base_size = BS) +
+ labs(
+ title = "Exposure to PM2.5 from heating in Basel",
+ x = "Time",
+ y = "Average PM2.5 (µg/m3)"
+ )
> ggplot(subset(expo, EfficiencyPolicy == "BAU"), aes(x = Time, weight = exposureResult, fill = Heating)) + geom_bar(binwidth = 5) +
facet_grid(FuelPolicy ~ RenovationPolicy) + theme_gray(base_size = BS) +
+ labs(
+ title = "Exposure to PM2.5 from heating in Basel",
+ x = "Time",
+ y = "Average PM2.5 (µg/m3)"
+ )
> cases <- suomenna(truncateIndex(totcases, cols = "Heating", bins = 3))
> ggplot(subset(cases, EfficiencyPolicy == "BAU" & FuelPolicy == "BAU"), aes(x = Time, weight = totcasesResult, fill = Heating))+geom_bar(binwidth = 5)
+
+ facet_grid(Response ~ RenovationPolicy) +
+ scale_x_continuous(breaks = c(2010, 2030, 2050)) +
+ theme_gray(base_size = BS) +
+ labs(
+ title = "Health effects of PM2.5 from heating in Basel",
+ x = "Time",
+ y = "Health effects (deaths /a)"
+ )
> if(figstofile) ggsave("Figure8.eps", width = 11, height = 7)
> cat("Total DALYs/a by different combinations of policy options.\n")
Total DALYs/a by different combinations of policy options.

> dal <- suomenna(DALYs)
> dal <- subset(dal, Response == "Total mortality")
> oprint(aggregate(dal["DALYsResult"], by = dal[c("Time", "EfficiencyPolicy", "RenovationPolicy", "FuelPolicy")], FUN = sum))
```

	Time	EfficiencyPolicy	RenovationPolicy	FuelPolicy	DALYsResult
1	2010.00	BAU	BAU	BAU	91.74
2	2020.00	BAU	BAU	BAU	90.39
3	2030.00	BAU	BAU	BAU	88.79
4	2040.00	BAU	BAU	BAU	87.26
5	2050.00	BAU	BAU	BAU	85.87
6	2010.00	Active efficiency	BAU	BAU	91.74
7	2020.00	Active efficiency	BAU	BAU	90.35
8	2030.00	Active efficiency	BAU	BAU	88.71
9	2040.00	Active efficiency	BAU	BAU	87.16
10	2050.00	Active efficiency	BAU	BAU	85.76
11	2010.00	BAU	Active renovation	BAU	91.74
12	2020.00	BAU	Active renovation	BAU	87.89
13	2030.00	BAU	Active renovation	BAU	84.48
14	2040.00	BAU	Active renovation	BAU	81.70
15	2050.00	BAU	Active renovation	BAU	79.50
16	2010.00	Active efficiency	Active renovation	BAU	91.74
17	2020.00	Active efficiency	Active renovation	BAU	87.85
18	2030.00	Active efficiency	Active renovation	BAU	84.40
19	2040.00	Active efficiency	Active renovation	BAU	81.61
20	2050.00	Active efficiency	Active renovation	BAU	79.39
21	2010.00	BAU	Total renovation	BAU	91.74
22	2020.00	BAU	Total renovation	BAU	67.88
23	2030.00	BAU	Total renovation	BAU	68.02
24	2040.00	BAU	Total renovation	BAU	68.28
25	2050.00	BAU	Total renovation	BAU	68.59

26	2010.00	Active efficiency	Total renovation	BAU	91.74
27	2020.00	Active efficiency	Total renovation	BAU	67.84
28	2030.00	Active efficiency	Total renovation	BAU	67.94
29	2040.00	Active efficiency	Total renovation	BAU	68.18
30	2050.00	Active efficiency	Total renovation	BAU	68.49
31	2010.00	BAU	BAU	Biofuel increase	91.74
32	2020.00	BAU	BAU	Biofuel increase	98.32
33	2030.00	BAU	BAU	Biofuel increase	96.58
34	2040.00	BAU	BAU	Biofuel increase	94.92
35	2050.00	BAU	BAU	Biofuel increase	93.41
36	2010.00	Active efficiency	BAU	Biofuel increase	91.74
37	2020.00	Active efficiency	BAU	Biofuel increase	98.28
38	2030.00	Active efficiency	BAU	Biofuel increase	96.49
39	2040.00	Active efficiency	BAU	Biofuel increase	94.82
40	2050.00	Active efficiency	BAU	Biofuel increase	93.29
41	2010.00	BAU	Active renovation	Biofuel increase	91.74
42	2020.00	BAU	Active renovation	Biofuel increase	95.61
43	2030.00	BAU	Active renovation	Biofuel increase	91.90
44	2040.00	BAU	Active renovation	Biofuel increase	88.88
45	2050.00	BAU	Active renovation	Biofuel increase	86.49
46	2010.00	Active efficiency	Active renovation	Biofuel increase	91.74
47	2020.00	Active efficiency	Active renovation	Biofuel increase	95.56
48	2030.00	Active efficiency	Active renovation	Biofuel increase	91.81
49	2040.00	Active efficiency	Active renovation	Biofuel increase	88.78
50	2050.00	Active efficiency	Active renovation	Biofuel increase	86.37
51	2010.00	BAU	Total renovation	Biofuel increase	91.74
52	2020.00	BAU	Total renovation	Biofuel increase	73.90
53	2030.00	BAU	Total renovation	Biofuel increase	74.00
54	2040.00	BAU	Total renovation	Biofuel increase	74.27
55	2050.00	BAU	Total renovation	Biofuel increase	74.61
56	2010.00	Active efficiency	Total renovation	Biofuel increase	91.74
57	2020.00	Active efficiency	Total renovation	Biofuel increase	73.85
58	2030.00	Active efficiency	Total renovation	Biofuel increase	73.91
59	2040.00	Active efficiency	Total renovation	Biofuel increase	74.17
60	2050.00	Active efficiency	Total renovation	Biofuel increase	74.50

```
> ggplot(subset(dal, FuelPolicy == "BAU"), aes(x = Time, weight = DALYsResult, fill = Heating))+geom_bar(binwidth = 5) +
+ facet_grid(EfficiencyPolicy ~ RenovationPolicy) +
+ theme_gray(base_size = BS) +
+ labs(
+ title = "Health effects in DALYs of PM2.5 from heating in Basel",
+ x = "Time",
+ y = "Health effects (DALY /a)"
+ )
> ggplot(subset(dal, Time == 2030), aes(x = FuelPolicy, weight = DALYsResult, fill = Heating))+geom_bar() +
+ facet_grid(EfficiencyPolicy ~ RenovationPolicy) +
+ theme_gray(base_size = BS) +
+ labs(
+ title = "Health effects in DALYs of PM2.5 from heating in Basel 2030",
+ x = "Biofuel policy in district heating",
+ y = "Health effects (DALY /a)"
+ )
> if(TRUE) {
+ koord <- tidy(opbase.data("Op_en7044", subset = "Locations of postal codes"))
+ colnames(koord) <- c("Emission_site", "X", "Y")
+ koord$Result <- 1
+ koord <- Ovariable("koord", output = koord, marginal = c(TRUE, TRUE, TRUE, FALSE))
+
+ emis <- emissions * koord
+
+ emis@output <- subset(emis@output,
+ RenovationPolicy == "BAU" &
+ EfficiencyPolicy == "BAU" &
+ FuelPolicy == "BAU" &
+ Pollutant == "PM2.5" &
+ Time == "2020"
+ )
+ emis <- oapply(emis, INDEX = c("Emission_site", "X", "Y"), FUN = sum)
```

```
+
+ MyRmap(
+ ova2spat(
+ emis,
+ coord = c("X", "Y"),
+ proj4string = "+init=epsg:21781"
+ ), # Swiss Land Survey uses CH1903
+ # http://spatialreference.org/ref/epsg/21782/
+ # http://en.wikipedia.org/wiki/Swiss_coordinate_system
+ plotvar = "Result",
+ legend_title = "PM2.5 emissions (ton/a)",
+ numbins = 4,
+ pch = 19,
+ cex = sqrt(result(emis)) * 3
+ )
+ # Map saved manually to .eps with width = 1280, height = 960 px.
+ }
[1] "http://maps.google.com/maps/api/staticmap?
center=47.5581571977573,7.59956129760629&zoom=13&size=640x480&maptype=mobile&format=png32&sensor=true"

> if(saveobjects) {
+ objects.put(list = ls())
+ cat(c("All objects archived. Write down the key of the run to retrieve them with objects.get. Objects: ",
+ ls(), "\n"))
+ }
All objects archived. Write down the key of the run to retrieve them with objects.get. Objects: ana2ova bgexposure BS bui buildings BW cases
changeBuildings collapsemarg dal DALYs DecefficiencyShares DecfuelShares decisions DecrenovationRate disincidence dose dummy DW efficiencyRatio
efficiencyShares emis emissionFactors emissionLocations emissions energyFactor energyUse ERF ERF_diox ERF_env ERF_mehg ERF_omega3 expo
exposure figstofile findrest finnish forgetDecisions frexposed fuelShares fuelSharesgeneric fuelUse hea heatingShares iF koord L makeTimeline MyPlotKML
MyPointKML MyRmap obstime ograph orbind2 ova2spat population renovationRate renovationRatio renovationShares RR saveobjects stockBuildings
suomenna testforrow threshold threshold_diox threshold_env threshold_mehg threshold_omega3 timepoints timing totcases truncateIndex wiki_username
```

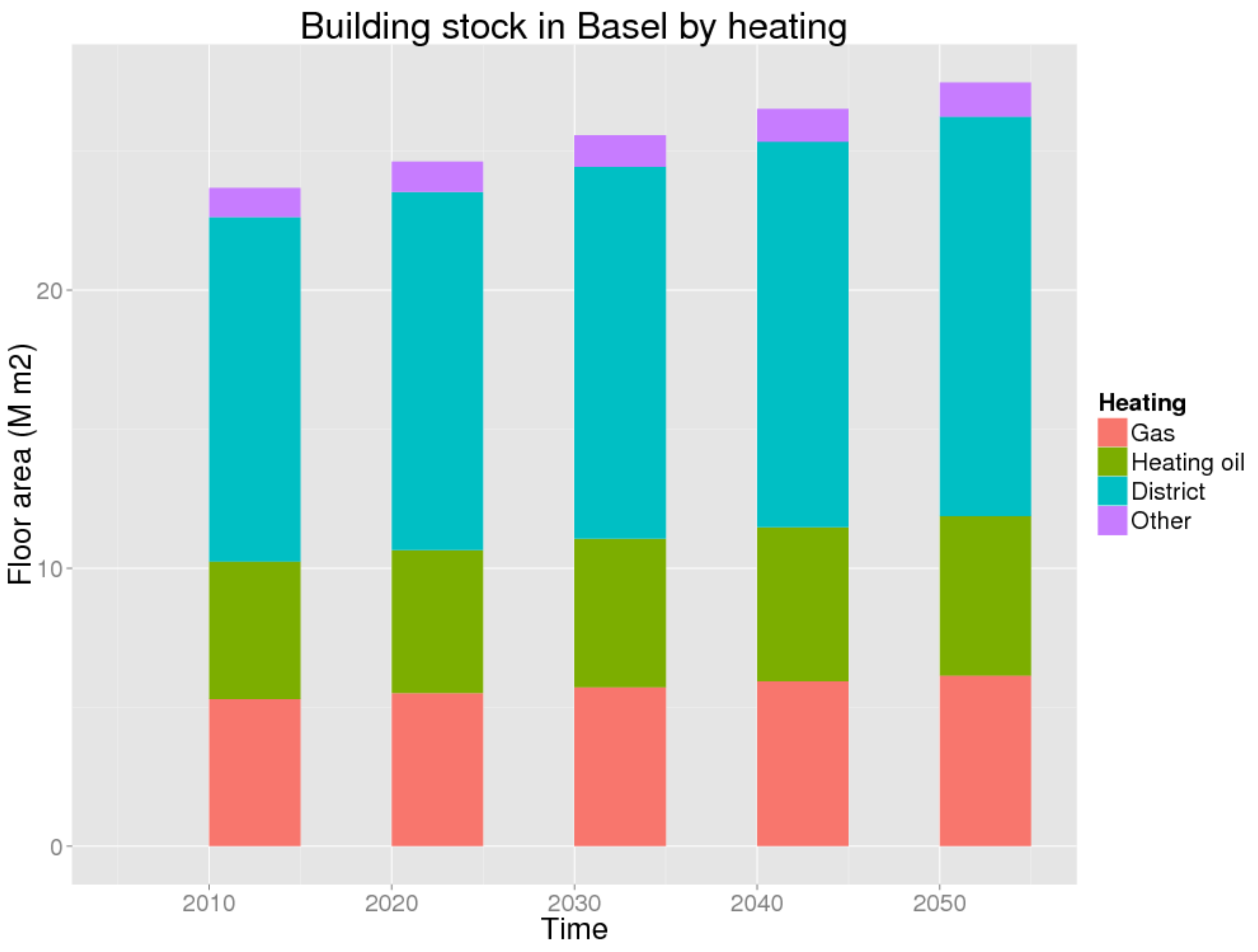

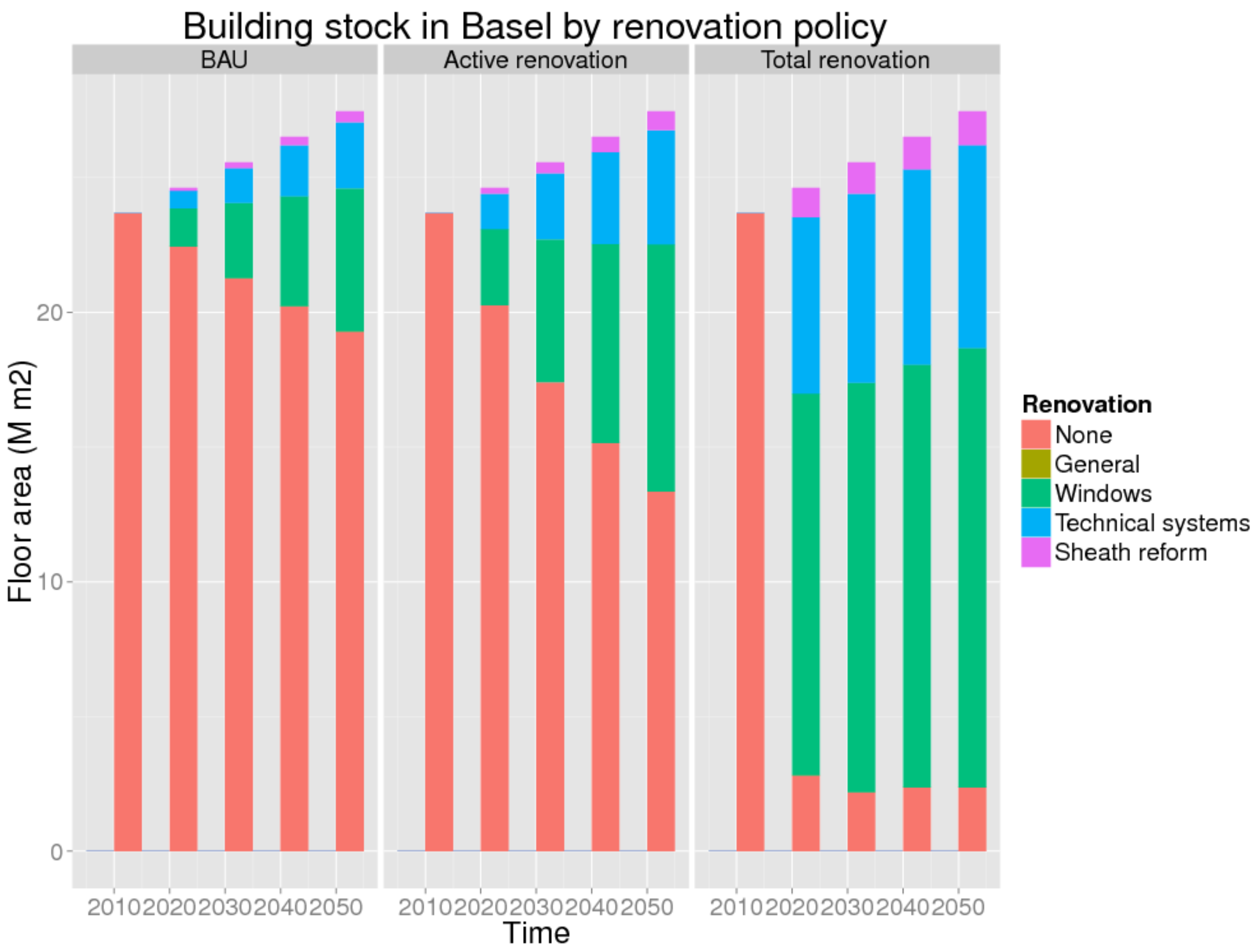
Building stock in Basel by renovation policy
BAU
Active renovation
Total renovation
Floor area (M m2)
0
10
20
2010
2020
2030
2040
2050
Time
Renovation
None
General
Windows
Technical systems
Sheath reform

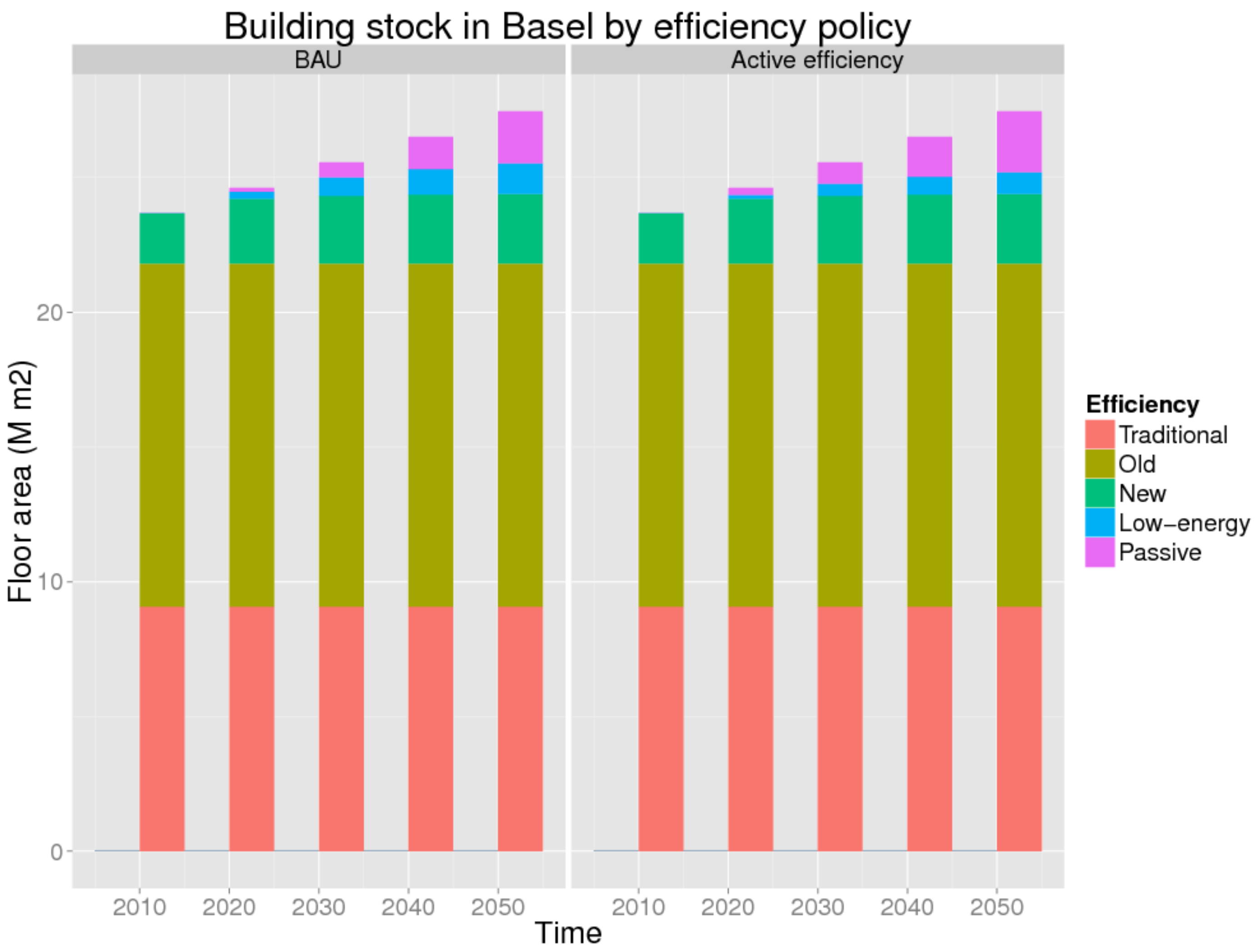
Building stock in Basel by efficiency policy
BAU
Active efficiency
Floor area (M m2)
0
10
20
2010
2020
2030
2040
2050
Time
Efficiency
Traditional
Old
New
Low−energy
Passive

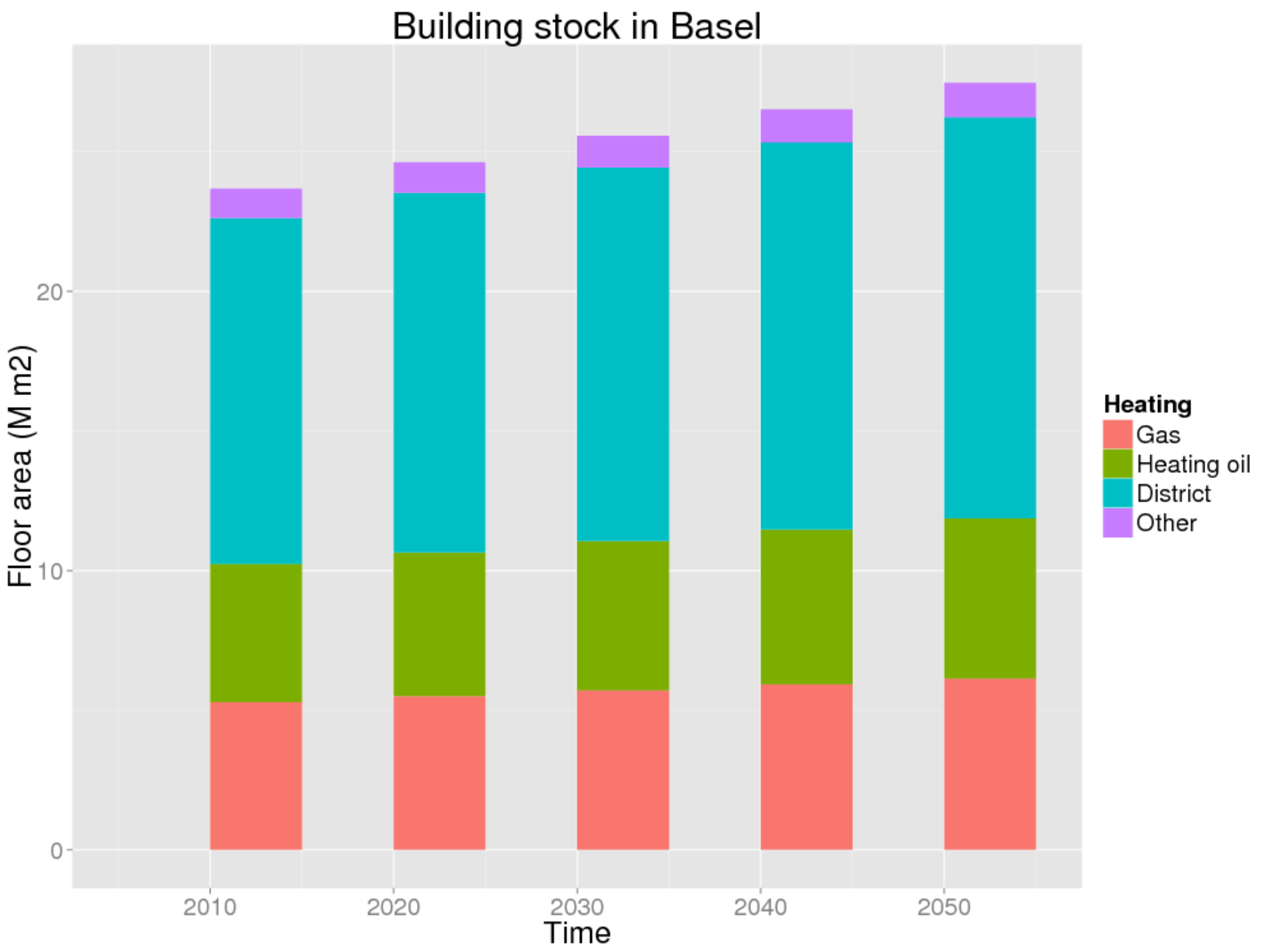
Building stock in Basel
Floor area (M m2)
0
10
20
2010
2020
2030
2040
2050
Time
Heating
Gas
Heating oil
District
Other

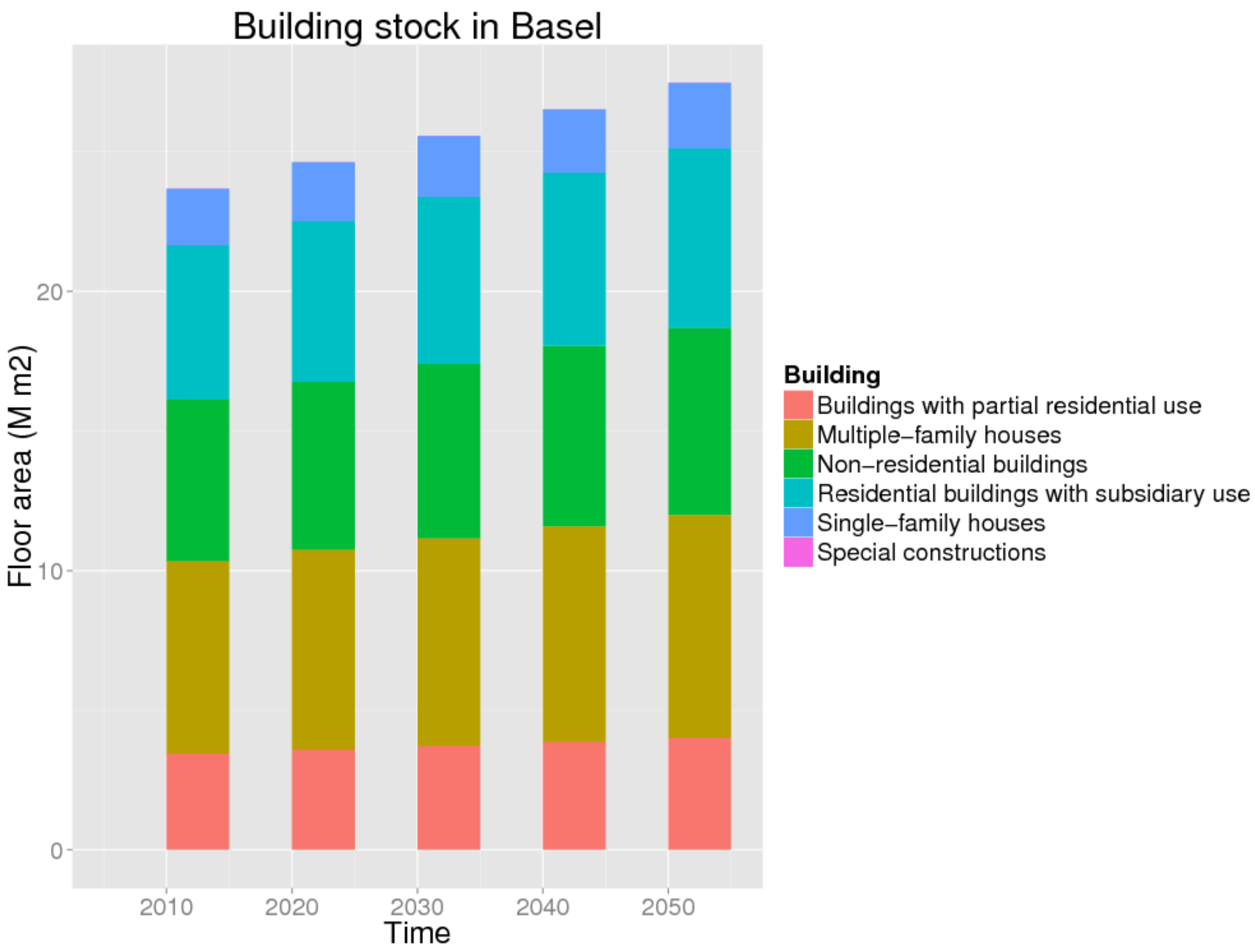
Building stock in Basel
Floor area (M m2)
0
10
20
2010
2020
2030
2040
2050
Time
Building
Buildings with partial residential use
Multiple−family houses
Non−residential buildings
Residential buildings with subsidiary use
Single−family houses
Special constructions

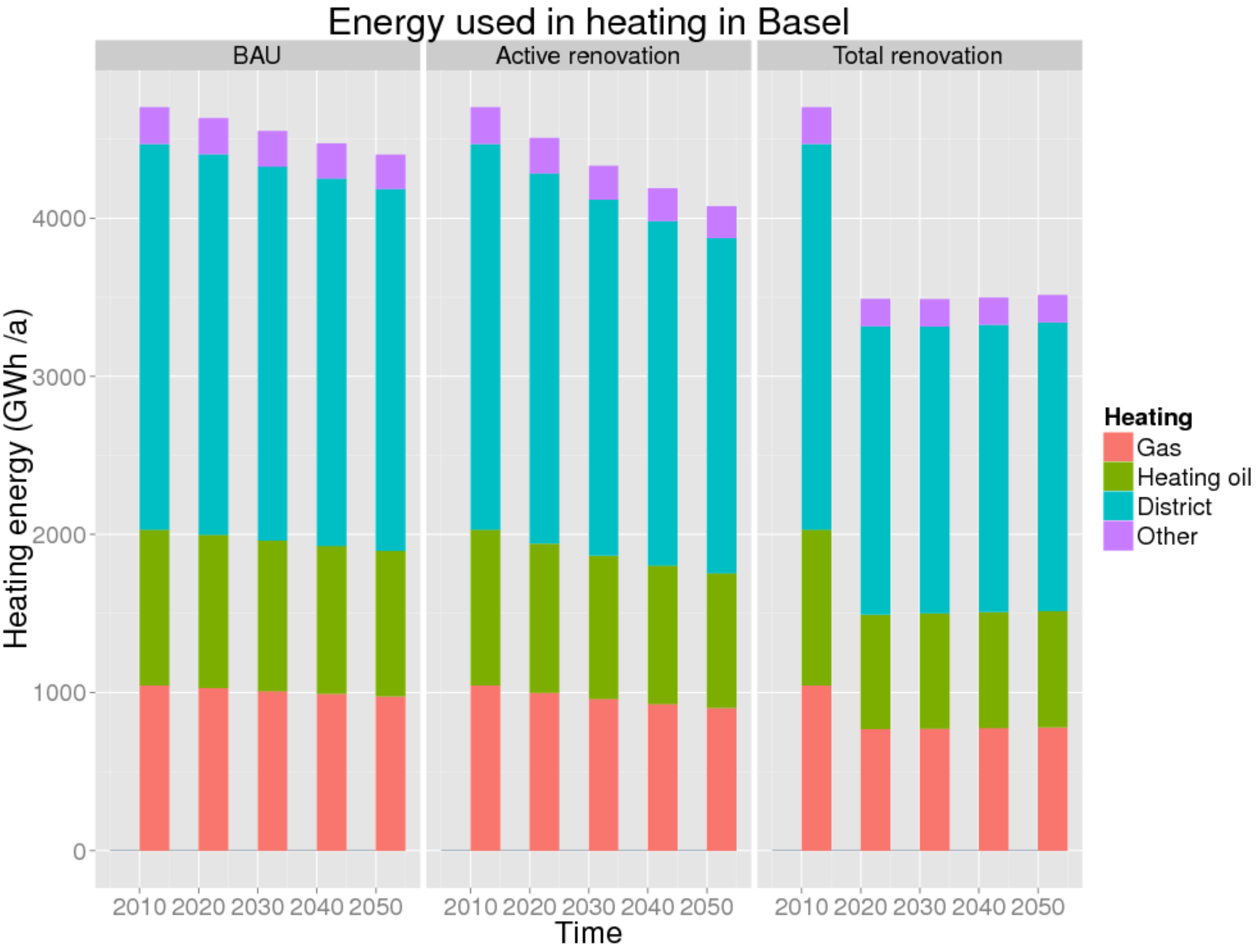

Energy used in heating in Basel
BAU
Active renovation
Total renovation
Heating energy (GWh /a)
0
1000
2000
3000
4000
2010 2020 2030 2040 2050
2010 2020 2030 2040 2050
2010 2020 2030 2040 2050
Time
Heating
Gas
Heating oil
District
Other

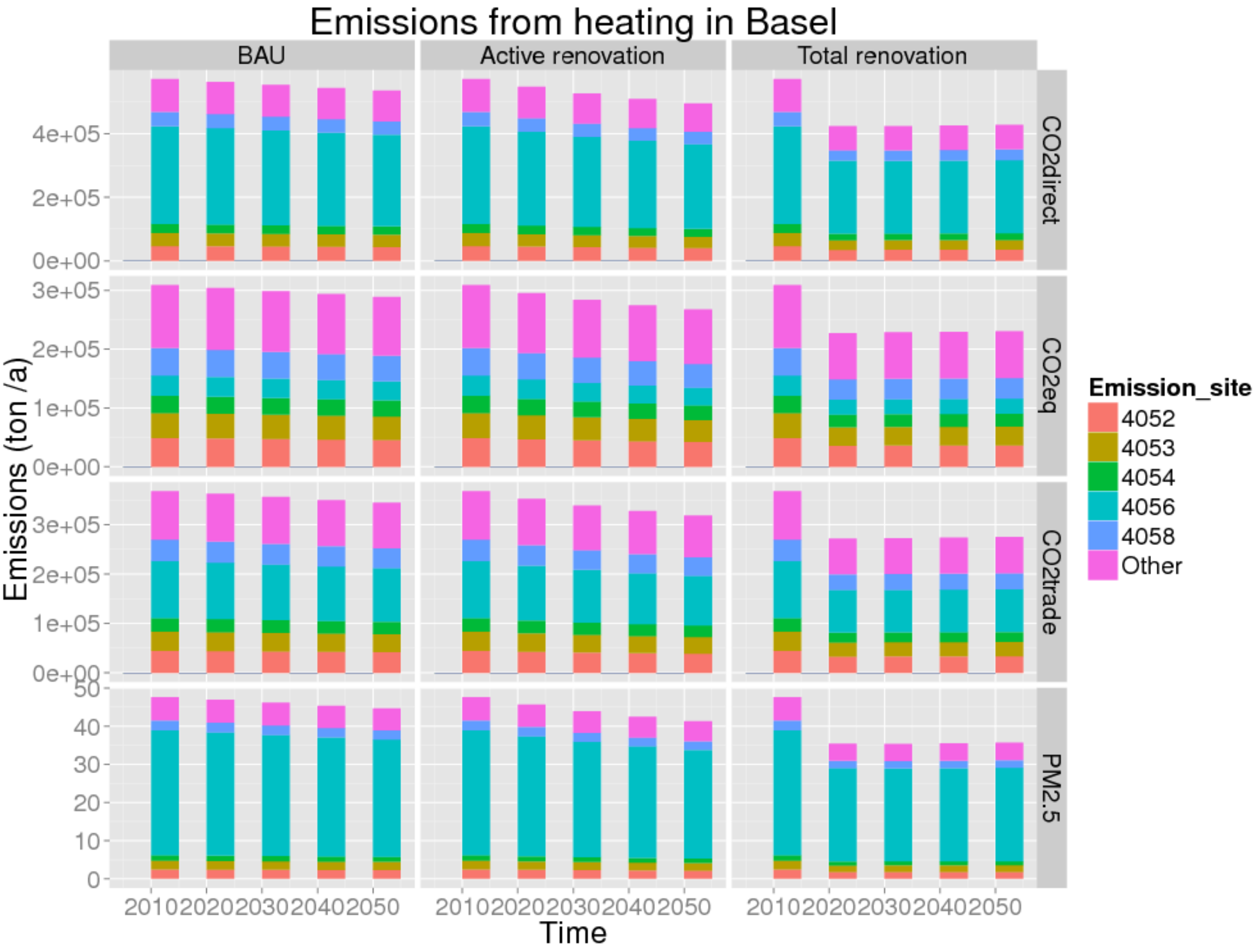
Emissions from heating in Basel
BAU
Active renovation
Total renovation
CO2direct
CO2eq
CO2trade
PM2.5
Emissions (ton /a)
Time
2010
2020
2030
2040
2050
4e+05
2e+05
0e+00
3e+05
1e+05
50
40
30
20
10
0
Emission_site
4052
4053
4054
4056
4058
Other

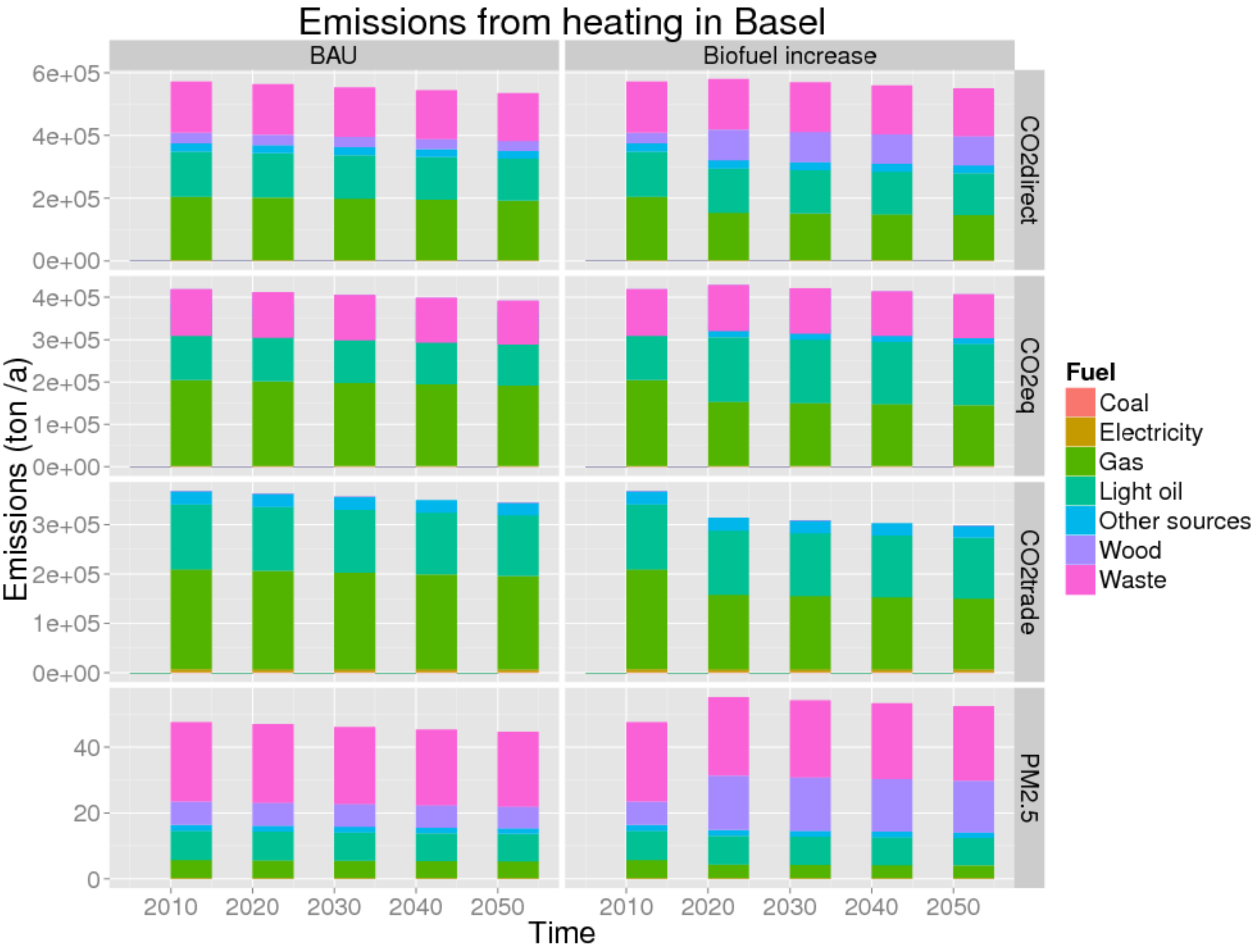
Emissions from heating in Basel
BAU
Biofuel increase
CO2direct
CO2eq
CO2trade
PM2.5
Emissions (ton /a)
6e+05
4e+05
2e+05
0e+00
3e+05
1e+05
40
20
0
2010
2020
2030
2040
2050
Time
Fuel
Coal
Electricity
Gas
Light oil
Other sources
Wood
Waste

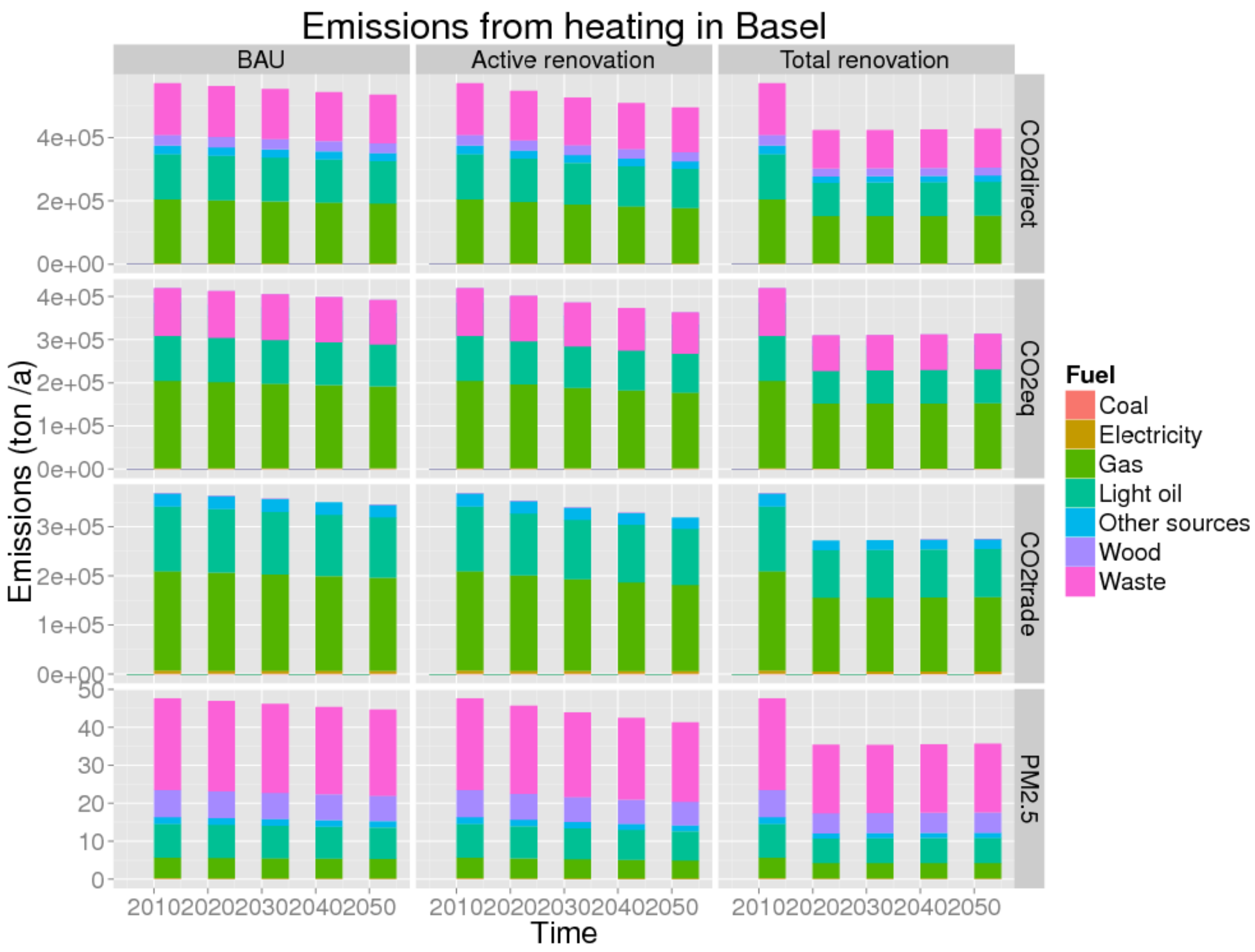
Emissions from heating in Basel
BAU
Active renovation
Total renovation
CO2direct
CO2eq
CO2trade
PM2.5
Emissions (ton /a)
4e+05
2e+05
0e+00
3e+05
1e+05
50
40
30
20
10
0
2010
2020
2030
2040
2050
Time
Fuel
Coal
Electricity
Gas
Light oil
Other sources
Wood
Waste

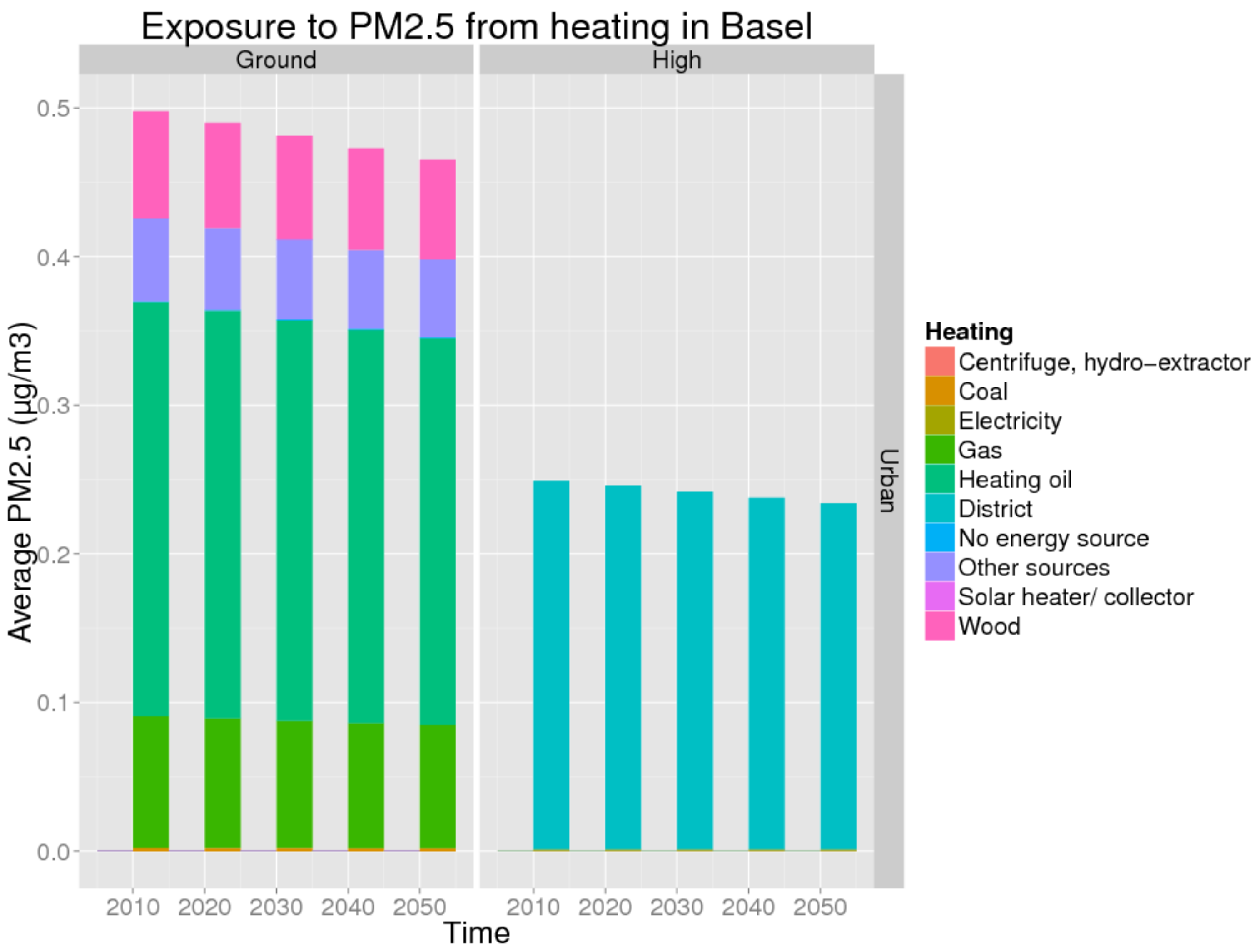
Exposure to PM2.5 from heating in Basel
Ground
High
Urban
0.5
0.4
0.3
0.2
0.1
0.0
Average PM2.5 (µg/m3)
2010 2020 2030 2040 2050
2010 2020 2030 2040 2050
Time
Heating
Centrifuge, hydro−extractor
Coal
Electricity
Gas
Heating oil
District
No energy source
Other sources
Solar heater/ collector
Wood

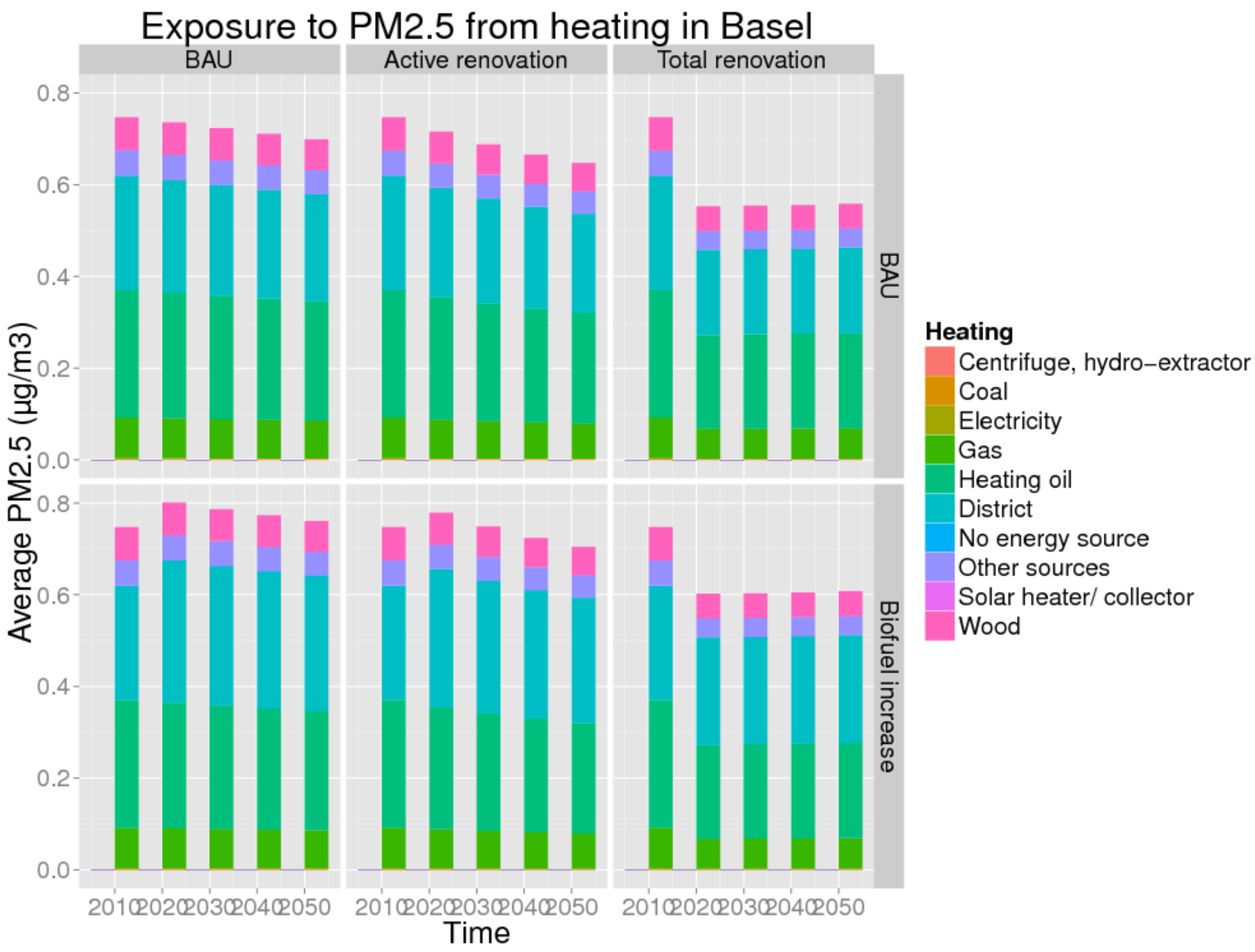
Exposure to PM2.5 from heating in Basel
BAU
Active renovation
Total renovation
BAU
Biofuel increase
Average PM2.5 (µg/m3)
0.0
0.2
0.4
0.6
0.8
2010
2020
2030
2040
2050
Time
Heating
Centrifuge, hydro−extractor
Coal
Electricity
Gas
Heating oil
District
No energy source
Other sources
Solar heater/ collector
Wood

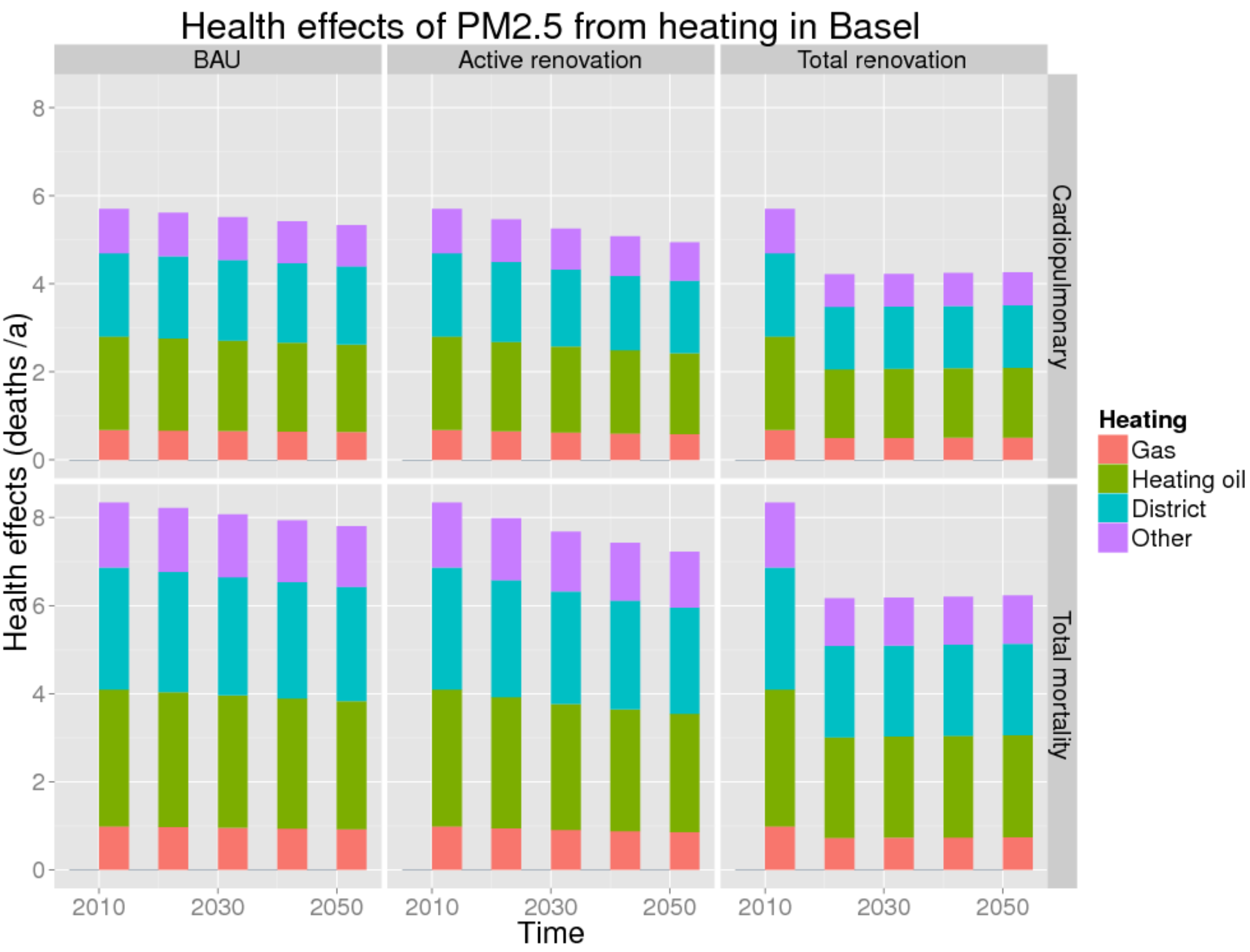
Health effects of PM2.5 from heating in Basel
BAU
Active renovation
Total renovation
Cardiopulmonary
Total mortality
Health effects (deaths /a)
0
2
4
6
8
2010
2030
2050
Time
Heating
Gas
Heating oil
District
Other

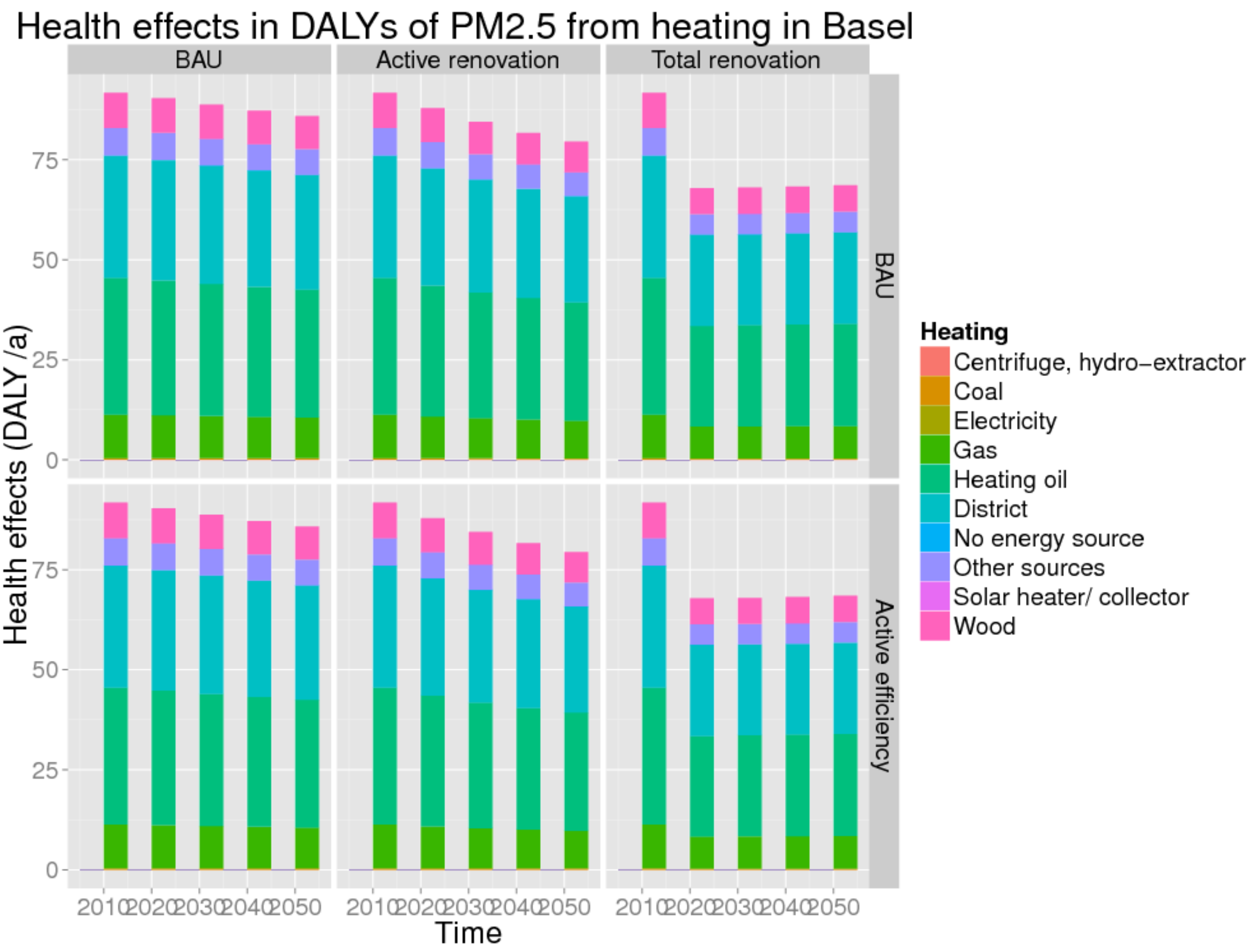
Health effects in DALYs of PM2.5 from heating in Basel
BAU
Active renovation
Total renovation
BAU
Active efficiency
Health effects (DALY /a)
0
25
50
75
2010
2020
2030
2040
2050
Time
Heating
Centrifuge, hydro−extractor
Coal
Electricity
Gas
Heating oil
District
No energy source
Other sources
Solar heater/ collector
Wood

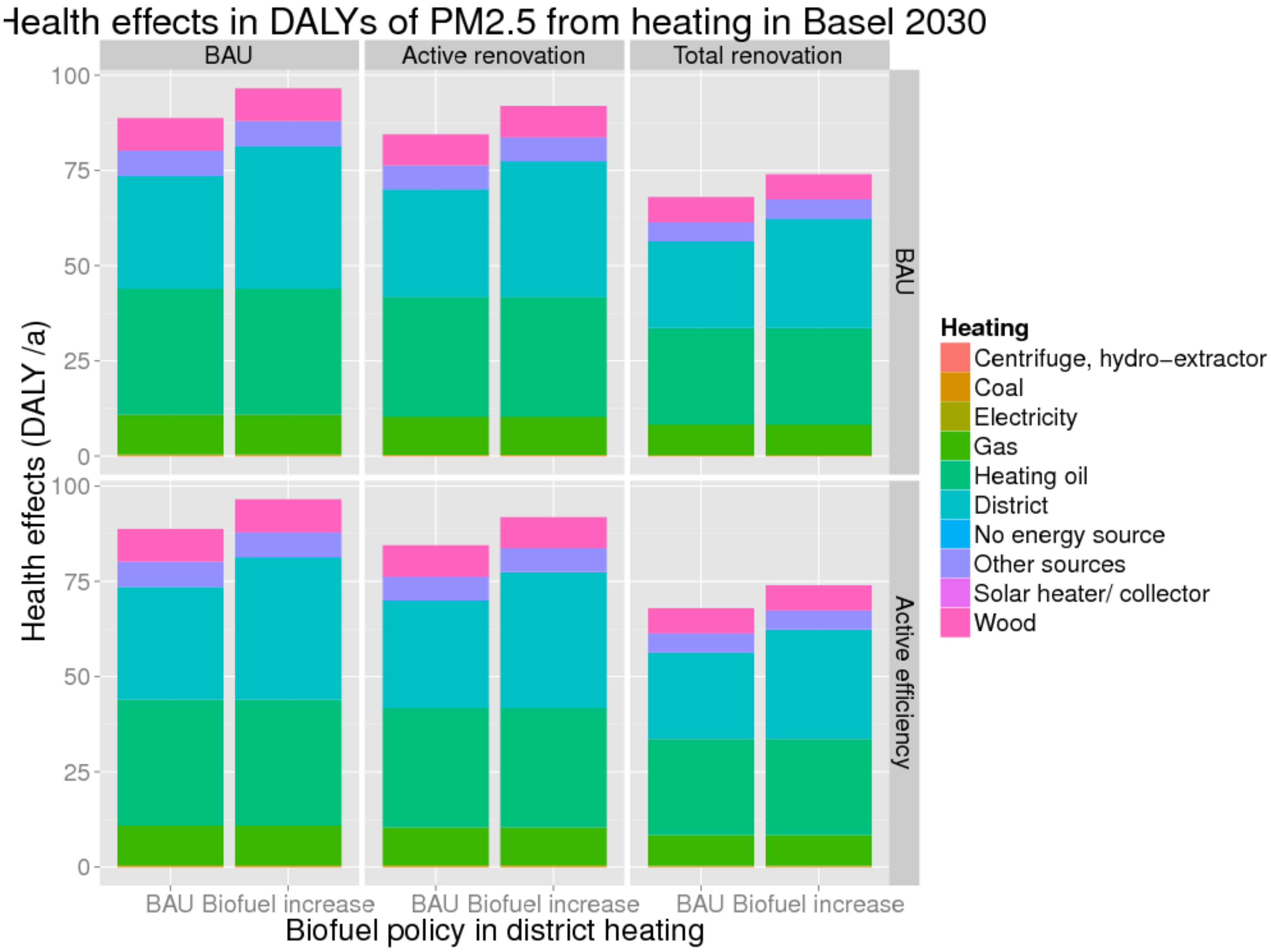

Health effects in DALYs of PM2.5 from heating in Basel 2030
BAU
Active renovation
Total renovation
BAU
Active efficiency
Health effects (DALY /a)
0
25
50
75
100
BAU Biofuel increase
BAU Biofuel increase
BAU Biofuel increase
Biofuel policy in district heating
Heating
Centrifuge, hydro−extractor
Coal
Electricity
Gas
Heating oil
District
No energy source
Other sources
Solar heater/ collector
Wood

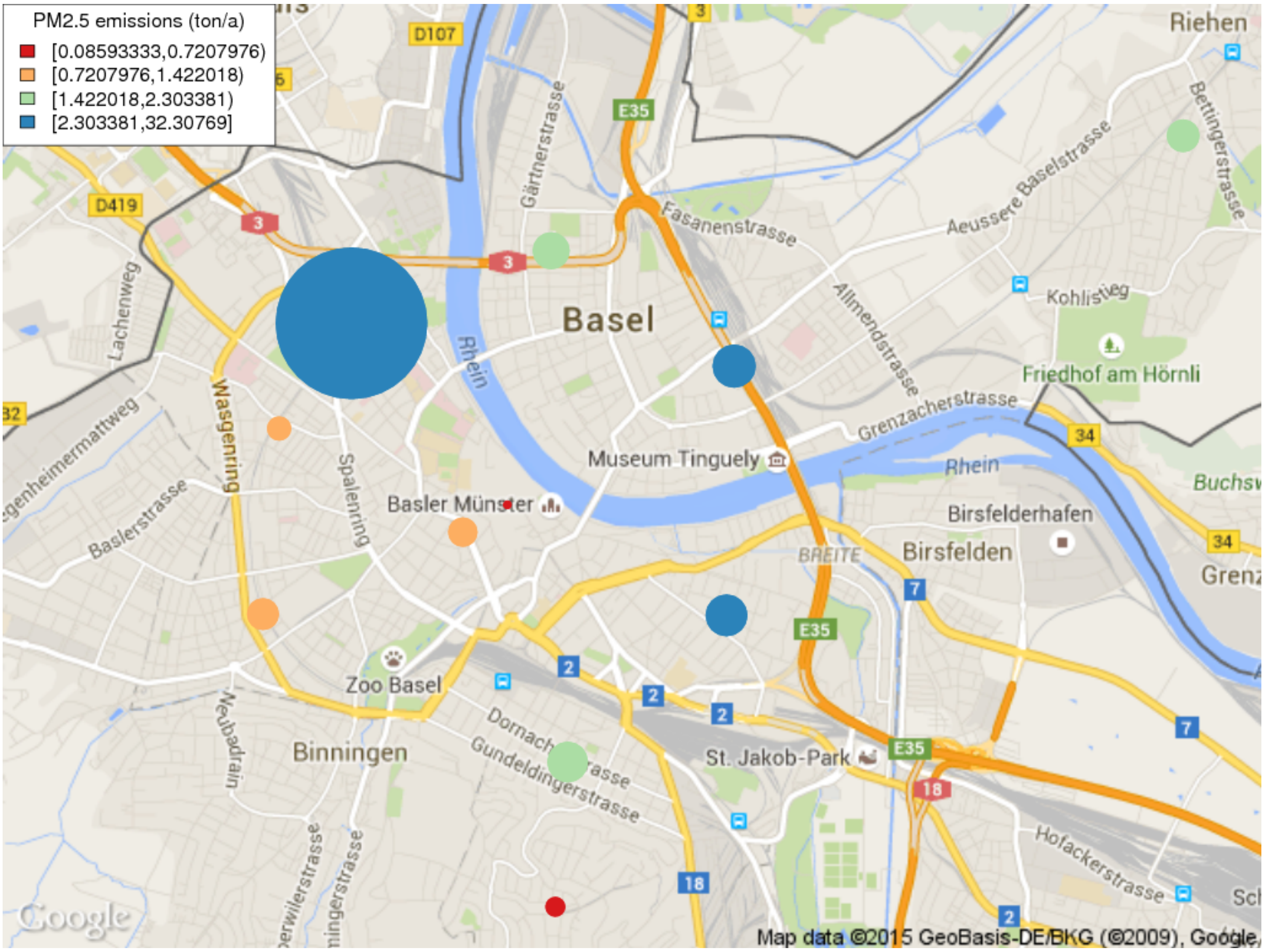
PM2.5 emissions (ton/a)
[0.08593333,0.7207976)
[0.7207976,1.422018)
[1.422018,2.303381)
[2.303381,32.30769]
Basel
Riehen
Rhein
Museum Tinguely
Basler Münster
Birsfelderhafen
Birsfelden
Zoo Basel
Binningen
St. Jakob-Park
Friedhof am Hörnli
Grenzacherstrasse
Fasanenstrasse
Allmendstrasse
Aeussere Baselstrasse
Kohlistieg
Bettingerstrasse
Gärtnerstrasse
Spalenring
Wasgenring
Baslerstrasse
Lachenweg
Neubadrain
Dornacherstrasse
Gundeldingerstrasse
Hofackerstrasse
BREITE
Map data ©2015 GeoBasis-DE/BKG (©2009), Google
