# Supplementary material for: Building-related health impacts in European and Chinese cities: a scalable assessment method
Source: Environ Health. 2015 Dec 14;14:93. doi: 10.1186/s12940-015-0082-z (PMC4678713; doi:10.1186/s12940-015-0082-z)
Supplement: Additional file 4: — Importance analysis for Kuopio with 1000 iterations. R-model run for the importance analysis for Kuopio case with the R-code and results. (PDF 245 kb) [file 12940_2015_82_MOESM4_ESM.pdf]

R-tools

Ran at 2015-07-26 22:48:47

```
> wiki_username <- "Jouni"
> num <- 10
> ### THIS CODE IS FROM PAGE [[Climate change policies and health in Kuopio]] (Op_en5461, code_name = "")
> library(OpasnetUtils)
> library(ggplot2)
> ### Technical parameters
> openv.setN(750)
> #rm(list = ls()) # Remove existing objects (necessary on your own computer)
> saveobjects <- TRUE
> objects.latest("Op_en6007", code_name = "answer") # [[OpasnetUtils/Drafts]] findrest
> obstime <- Ovariable("obstime", data = data.frame(Obsyear = factor(seq(2010, 2030, 10), ordered = TRUE), Result = 1))
> ## Additional index needed in followup of ovariables efficiencyShares and stockBuildings
> year <- Ovariable("year", data = data.frame(
+ Constructed = factor(
+ c("1799-1899", "1900-1909", "1910-1919", "1920-1929", "1930-1939", "1940-1949",
+ "1950-1959", "1960-1969", "1970-1979", "1980-1989", "1990-1999",
+ "2000-2010", "2011-2019", "2020-2029", "2030-2039", "2040-2049"
+ ),
+ ordered = TRUE
+ ),
+ Time = c(1880, 1910 + 0:14 * 10),
+ Result = 1
+ ))
> ###################### Decisions
> decisions <- opbase.data('Op_en5461', subset = "Decisions") # [[Climate change policies and health in Kuopio]]
> DecisionTableParser(decisions)
> # Remove previous decisions, if any.
> forgetDecisions <- function() {
+ for(i in ls(envir = openv)) {
+ if("dec_check" %in% names(openv[[i]])) openv[[i]]$dec_check <- FALSE
+ }
+ return(cat("Decisions were forgotten.\n"))
+ }
> forgetDecisions()
Decisions were forgotten.

> ############################ IMPORT DATA AND MODELS
> objects.latest("Op_en5417", code_name = "initiate") # [[Population of Kuopio]]
> objects.latest("Op_en5932", code_name = "initiatetest") # [[Building stock in Kuopio]] Building ovariables:
> objects.latest("Op_en6289", code_name = "buildingstest") # [[Building model]] # Generic building model.
> ###################### Energy and emissions
> objects.latest("Op_en5488", code_name = "energyUseAnnual") # [[Energy use of buildings]] energyUse
> objects.latest("Op_en5488", code_name = "efficiencyShares") # [[Energy use of buildings]]
> objects.latest("Op_en2791", code_name = "emissionstest") # [[Emission factors for burning processes]]
> objects.latest("Op_en2791", code_name = "emissionFactors") # [[Emission factors for burning processes]]
> objects.latest("Op_en7328", code_name = "emissionLocations") # [[Kuopio energy production]]
> objects.latest("Op_en7328", code_name = "fuelShares") # [[Kuopio energy production]]
> objects.latest("Op_en5141", code_name = "fuelUse") # [[Energy balance]]
> ## Exposure and health assessment
> objects.latest("Op_en5813", code_name = "exposure") # [[Intake fractions of PM]] uses Humbert iF as default.
> objects.latest('Op_en2261', code_name = 'totcases') # [[Health impact assessment]] totcases and dependencies.
> objects.latest('Op_en5461', code_name = 'DALYs') # [[Climate change policies and health in Kuopio]] DALYs, DW, L
> ##################### CALCULATIONS
> constructionAreas <- EvalOutput(constructionAreas)
> constructionAreas@output$City_area <- "City centre"# We are not interested in locations in this analysis.
> constructionAreas <- oapply(constructionAreas, cols = "", FUN = sum)
> renovationRate <- EvalOutput(renovationRate) * 10 # Rates for 10-year periods
> renovationShares <- EvalOutput(renovationShares)
> stockBuildings <- EvalOutput(stockBuildings)
> stockBuildings@output$City_area <- "City centre"
> stockBuildings@output$Building <- "Apartment houses"
> stockBuildings <- oapply(stockBuildings, cols = c(""), FUN = sum)
> changeBuildings <- EvalOutput(changeBuildings)
[1] "Column Iter treated with fillna (difference between historical buildings and construction)." [1] "Column EfficiencyPolicy treated with fillna (difference between historical buildings and construction)."

> changeBuildings@output$City_area <- "City centre"
> changeBuildings@output$Building <- "Apartment houses"
> changeBuildings@output <- changeBuildings@output[changeBuildings@output$EfficiencyPolicy == "BAU" , ]
> changeBuildings <- oapply(changeBuildings, cols = c(""), FUN = sum)
> buildings <- EvalOutput(buildings)
[1] "Column RenovationPolicy treated with fillna (difference between buil and renovate)."

> buildings@output <- buildings@output[buildings@output$Time == "2030" , ]
> energyUse <- EvalOutput(energyUse)
> energyUse <- oapply(energyUse, cols = c(
```

```
+ "Efficiency",
+ "Renovation"
+ ), FUN = sum)
> fuelUse <- EvalOutput(fuelUse)
> fuelUse <- fuelUse * 1E-3 *3600 # kWh -> MJ
> fuelUse <- oapply(fuelUse, cols = c(
+ "Time"
+ ), FUN = sum)
> emissions <- EvalOutput(emissions)
> emissions <- oapply(emissions, cols = c(
+ "Fuel",
+ "City_area",
+ "Emission_site",
+ "Heating"
+ ), FUN = sum)
> population <- 1E+5 # stockBuildings is using another population to divide floor area into City areas.
> exposure <- EvalOutput(exposure)
> exposure@output <- exposure@output[exposure@output$Area == "Average" , ] # Kuopio is an average area,
> # rather than rural or urban.
> exposure <- oapply(exposure, cols = c(
+ "Emission_height",
+ "Area"
+ ), FUN = sum)
> totcases <- EvalOutput(totcases)
> totcases <- oapply(totcases, cols = c("Age", "Sex"), FUN = sum)
> DALYs <- EvalOutput(DALYs)
> cost <- Ovariable("cost",
+ dependencies = data.frame(Name = c("DALYs", "emissions")),
+ formula = function(...) {
+ dals <- DALYs
+ dals@output <- dals@output[dals@output$Time == "2030" , ]
+ dals <- oapply(DALYs, INDEX = c("EfficiencyPolicy", "RenovationPolicy", "FuelPolicy", "Iter"), FUN = sum)
+ emi <- emissions
+ emi@output <- emi@output[emi@output$Pollutant == "CO2direct" & emi@output$Time == "2030" , ]
+ emi <- oapply(emissions, INDEX = c("EfficiencyPolicy", "RenovationPolicy", "FuelPolicy", "Iter"), FUN = sum)
+ cost <- dals * 50000 + emi * 15
+ bau <- cost
+ bau@output <- subset(bau@output, FuelPolicy == "BAU" & RenovationPolicy == "BAU" & EfficiencyPolicy == "BAU")
+ bau <- unkeep(bau, cols = c( "EfficiencyPolicy", "RenovationPolicy", "FuelPolicy"), prevresults = TRUE)
+ bau <- bau * Ovariable(
+ output = data.frame(Objective = c("Direct", "BAU comparison"), Result = c(0, 1)),
+ marginal = c(TRUE, FALSE)
+ )
+ cost <- cost - bau
+ return(cost)
+ }
+ )
> t1 <- subset(construction@output, Building == "Apartment houses")
> t2 <- subset(efficiencyRatio@output, Efficiency == "New")
> t3 <- subset(efficiencyShares@output, Efficiency == "New" & Time == "2030" & EfficiencyPolicy == "BAU")
> t4 <- subset(emissionFactors@output, Fuel == "Peat" & Pollutant == "PM2.5")
> t5 <- subset(emissionFactors@output, Fuel == "Peat" & Pollutant == "CO2direct")
> t6 <- subset(energyFactor@output, Building == "Apartment houses" & Heating == "District")
> t7 <- subset(ERF@output, Exposure_agent == "PM2.5" & Response == "Total mortality")
> t8 <- subset(heatingShares@output, Heating == "District" & Building == "Apartment houses" & Time == "2030")
> t9 <- subset(renovationShares@output, RenovationPolicy == "Active renovation" & Renovation == "Sheath reform" & Obsyear == "2030")
> testvariable <- Ovariable("testvariable", data = data.frame(
+ Iter = c(
+ t1$Iter,
+ t2$Iter,
+ t3$Iter,
+ t4$Iter,
+ t5$Iter,
+ t6$Iter,
+ t7$Iter,
+ t8$Iter,
+ t9$Iter
+ ),
+ Variable = c(
+ rep("Construction of apartment houses", openv$N),
+ rep("Efficiency ratio", openv$N),
+ rep("Efficiency shares", openv$N),
+ rep("PM2.5 emission factor", openv$N),
+ rep("CO2 emission factor", openv$N),
+ rep("Energy factor of apartment houses", openv$N),
+ rep("Exposure-response funtion of PM2.5", openv$N),
+ rep("Future heating shares", openv$N),
+ rep("Shares of renovation types", openv$N)
+ ),
+ Result = c(
+ t1$constructionResult,
+ t2$efficiencyRatioResult,
```

```
+ t3$efficiencySharesResult,
+ t4$emissionFactorsResult,
+ t5$emissionFactorsResult,
+ t6$energyFactorResult,
+ t7$ERFResult,
+ t8$heatingSharesResult,
+ t9$renovationSharesResult
+ )
+ ))
> tornado <- Ovariable("tornado",
+ dependencies = data.frame(Name = c("cost", "testvariable")),
+ formula = function(...) {
+ test <- cost * testvariable
+ indices <- unique(test@output[test@marginal & ! colnames(test@output) %in% "Iter"])
+ out <- data.frame()
+ for(i in 1:nrow(indices)) {
+ temp <- merge(test, indices[i,])@output
+ temp <- cor(
+ temp[[paste(cost@name, "Result", sep = "")]],
+ temp[[paste(testvariable@name, "Result", sep = "")]],
+ method = "spearman"
+ )
+ out <- rbind(out, data.frame(indices[i,], Result = temp))
+ }
+ return(out)
+ }
+ )
> tornado <- EvalOutput(tornado)
> ggplot(tornado@output, aes(x = Variable, y = tornadoResult, colour = Objective)) +
+ geom_point(position = "jitter", size = 2)+coord_flip() + theme_gray(base_size = 24) +
+ labs(
+ title = "Importance diagram with direct or incremental cost",
+ y = "Spearman correlation vs. cost",
+ x = "Uncertain input variable to correlate"
+ )
> cortable <- tornado@output
> # Remove those that actually are not probabilistic
> cortable <- cortable[!cortable$Variable %in% c("CO2 emission factor", "Energy factor of apartment houses") , ]
> cortable <- reshape(
+ cortable,
+ v.names = "tornadoResult",
+ timevar = "Objective",
+ idvar = c("FuelPolicy", "RenovationPolicy", "EfficiencyPolicy", "Variable"),
+ drop = c("costSource", "testvariableSource", "tornadoSource"),
+ direction = "wide"
+ )
> cat("Spearman correlations between the outcome (cost) and probabilistic input variables. Cost is either A) direct cost or B) incremental compared with BAU.\n")
Spearman correlations between the outcome (cost) and probabilistic input variables. Cost is either A) direct cost or B) incremental compared with BAU.

> oprint(cortable)
```

	EfficiencyPolicy	RenovationPolicy	FuelPolicy	Variable	tornadoResult.Direct	tornadoResult.BAU comparison
1	Active efficiency	Active renovation	BAU	Construction of apartment houses	-0.03	-0.05
2	Active efficiency	Active renovation	BAU	Efficiency ratio	0.05	0.00
3	Active efficiency	Active renovation	BAU	Efficiency shares	-0.06	-0.06
4	Active efficiency	Active renovation	BAU	PM2.5 emission factor	0.03	-0.02
7	Active efficiency	Active renovation	BAU	Exposure-response funtion of PM2.5	0.74	0.71
8	Active efficiency	Active renovation	BAU	Future heating shares	0.00	0.01
9	Active efficiency	Active renovation	BAU	Shares of renovation types	-0.01	-0.01
19	BAU	Active renovation	BAU	Construction of apartment houses	0.04	0.03
20	BAU	Active renovation	BAU	Efficiency ratio	0.24	-0.10
21	BAU	Active renovation	BAU	Efficiency shares	-0.06	0.06
22	BAU	Active renovation	BAU	PM2.5 emission factor	0.23	-0.20
25	BAU	Active renovation	BAU	Exposure-response funtion of PM2.5	0.73	-0.75
26	BAU	Active renovation	BAU	Future heating shares	-0.01	0.01
27	BAU	Active renovation	BAU	Shares of renovation types	-0.04	-0.22
37	Active efficiency	BAU	BAU	Construction of apartment houses	-0.03	-0.05
38	Active efficiency	BAU	BAU	Efficiency ratio	0.05	0.00
39	Active efficiency	BAU	BAU	Efficiency shares	-0.06	-0.06
40	Active efficiency	BAU	BAU	PM2.5 emission factor	0.03	-0.02
				Exposure-response funtion of		

43	Active efficiency	BAU	BAU	PM2.5	0.74	0.71
44	Active efficiency	BAU	BAU	Future heating shares	0.00	0.01
45	Active efficiency	BAU	BAU	Shares of renovation types	-0.00	-0.00
55	BAU	BAU	BAU	Construction of apartment houses	0.03	
56	BAU	BAU	BAU	Efficiency ratio	0.24	
57	BAU	BAU	BAU	Efficiency shares	-0.06	
58	BAU	BAU	BAU	PM2.5 emission factor	0.23	
61	BAU	BAU	BAU	Exposure-response funtion of PM2.5	0.74	
62	BAU	BAU	BAU	Future heating shares	-0.01	
63	BAU	BAU	BAU	Shares of renovation types	-0.03	
73	Active efficiency	Effective renovation	BAU	Construction of apartment houses	-0.03	-0.05
74	Active efficiency	Effective renovation	BAU	Efficiency ratio	0.05	-0.01
75	Active efficiency	Effective renovation	BAU	Efficiency shares	-0.06	-0.06
76	Active efficiency	Effective renovation	BAU	PM2.5 emission factor	0.03	-0.03
79	Active efficiency	Effective renovation	BAU	Exposure-response funtion of PM2.5	0.74	0.71
80	Active efficiency	Effective renovation	BAU	Future heating shares	0.00	0.01
81	Active efficiency	Effective renovation	BAU	Shares of renovation types	0.01	0.01
91	BAU	Effective renovation	BAU	Construction of apartment houses	0.04	0.03
92	BAU	Effective renovation	BAU	Efficiency ratio	0.26	-0.08
93	BAU	Effective renovation	BAU	Efficiency shares	-0.07	0.04
94	BAU	Effective renovation	BAU	PM2.5 emission factor	0.24	-0.17
97	BAU	Effective renovation	BAU	Exposure-response funtion of PM2.5	0.72	-0.77
98	BAU	Effective renovation	BAU	Future heating shares	-0.01	0.01
99	BAU	Effective renovation	BAU	Shares of renovation types	0.01	0.20
109	Active efficiency	Active renovation	Biofuel increase	Construction of apartment houses	-0.03	-0.05
110	Active efficiency	Active renovation	Biofuel increase	Efficiency ratio	0.04	-0.00
111	Active efficiency	Active renovation	Biofuel increase	Efficiency shares	-0.06	-0.06
112	Active efficiency	Active renovation	Biofuel increase	PM2.5 emission factor	0.02	-0.03
115	Active efficiency	Active renovation	Biofuel increase	Exposure-response funtion of PM2.5	0.74	0.71
116	Active efficiency	Active renovation	Biofuel increase	Future heating shares	0.00	0.01
117	Active efficiency	Active renovation	Biofuel increase	Shares of renovation types	-0.01	-0.01
127	BAU	Active renovation	Biofuel increase	Construction of apartment houses	0.03	-0.04
128	BAU	Active renovation	Biofuel increase	Efficiency ratio	0.20	-0.27
129	BAU	Active renovation	Biofuel increase	Efficiency shares	-0.06	0.07
130	BAU	Active renovation	Biofuel increase	PM2.5 emission factor	0.15	-0.53
133	BAU	Active renovation	Biofuel increase	Exposure-response funtion of PM2.5	0.77	-0.26
134	BAU	Active renovation	Biofuel increase	Future heating shares	-0.01	0.02
135	BAU	Active renovation	Biofuel increase	Shares of renovation types	-0.04	-0.05
145	Active efficiency	BAU	Biofuel increase	Construction of apartment houses	-0.03	-0.05
146	Active efficiency	BAU	Biofuel increase	Efficiency ratio	0.04	-0.00
147	Active efficiency	BAU	Biofuel increase	Efficiency shares	-0.06	-0.06
148	Active efficiency	BAU	Biofuel increase	PM2.5 emission factor	0.02	-0.02
151	Active efficiency	BAU	Biofuel increase	Exposure-response funtion of PM2.5	0.74	0.71

152	Active efficiency	BAU	Biofuel increase	Future heating shares	0.00	0.01
153	Active efficiency	BAU	Biofuel increase	Shares of renovation types	-0.00	-0.00
163	BAU	BAU	Biofuel increase	Construction of apartment houses	0.03	-0.04
164	BAU	BAU	Biofuel increase	Efficiency ratio	0.19	-0.27
165	BAU	BAU	Biofuel increase	Efficiency shares	-0.06	0.06
166	BAU	BAU	Biofuel increase	PM2.5 emission factor	0.15	-0.52
169	BAU	BAU	Biofuel increase	Exposure-response funtion of PM2.5	0.77	-0.04
170	BAU	BAU	Biofuel increase	Future heating shares	-0.01	0.01
171	BAU	BAU	Biofuel increase	Shares of renovation types	-0.02	0.01
181	Active efficiency	Effective renovation	Biofuel increase	Construction of apartment houses	-0.03	-0.05
182	Active efficiency	Effective renovation	Biofuel increase	Efficiency ratio	0.04	-0.01
183	Active efficiency	Effective renovation	Biofuel increase	Efficiency shares	-0.06	-0.06
184	Active efficiency	Effective renovation	Biofuel increase	PM2.5 emission factor	0.02	-0.04
187	Active efficiency	Effective renovation	Biofuel increase	Exposure-response funtion of PM2.5	0.74	0.71
188	Active efficiency	Effective renovation	Biofuel increase	Future heating shares	0.00	0.01
189	Active efficiency	Effective renovation	Biofuel increase	Shares of renovation types	0.01	0.01
199	BAU	Effective renovation	Biofuel increase	Construction of apartment houses	0.04	-0.02
200	BAU	Effective renovation	Biofuel increase	Efficiency ratio	0.22	-0.25
201	BAU	Effective renovation	Biofuel increase	Efficiency shares	-0.06	0.06
202	BAU	Effective renovation	Biofuel increase	PM2.5 emission factor	0.15	-0.44
205	BAU	Effective renovation	Biofuel increase	Exposure-response funtion of PM2.5	0.76	-0.53
206	BAU	Effective renovation	Biofuel increase	Future heating shares	-0.01	0.02
207	BAU	Effective renovation	Biofuel increase	Shares of renovation types	0.01	0.12

```
> if(saveobjects) {
+ objects.put(list = ls())
+ cat(c("All objects archived. Write down the key of the run to retrieve them with objects.get. Objects: ",
+ ls(), "\n"))
+ }
```

All objects archived. Write down the key of the run to retrieve them with objects.get. Objects: ana2ova bgexposure buildings buildingTypes BW changeBuildings collapsemarg construction constructionAreas cortable cost DALYs DecefficiencyShares DecfuelShares decisions DecrenovationRate DecrenovationShares disincidence dose dummy DW efficiencyRatio efficiencyShares emissionFactors emissionLocations emissions energyFactor energyUse ERF ERF_diox ERF_env ERF_mehg ERF_omega3 exposure findrest forgetDecisions frexposed fuelShares fuelSharesgeneric fuelUse heatingShares iF L makeTimeline MyPlotKML MyPointKML MyRmap num obstime ograph orbind2 ova2spat population renovationRate renovationRatio renovationShares RR saveobjects stockBuildings t1 t2 t3 t4 t5 t6 t7 t8 t9 testforrow testvariable threshold threshold_diox threshold_env threshold_mehg threshold_omega3 timepoints timing tornado totcases truncateIndex wiki_username year

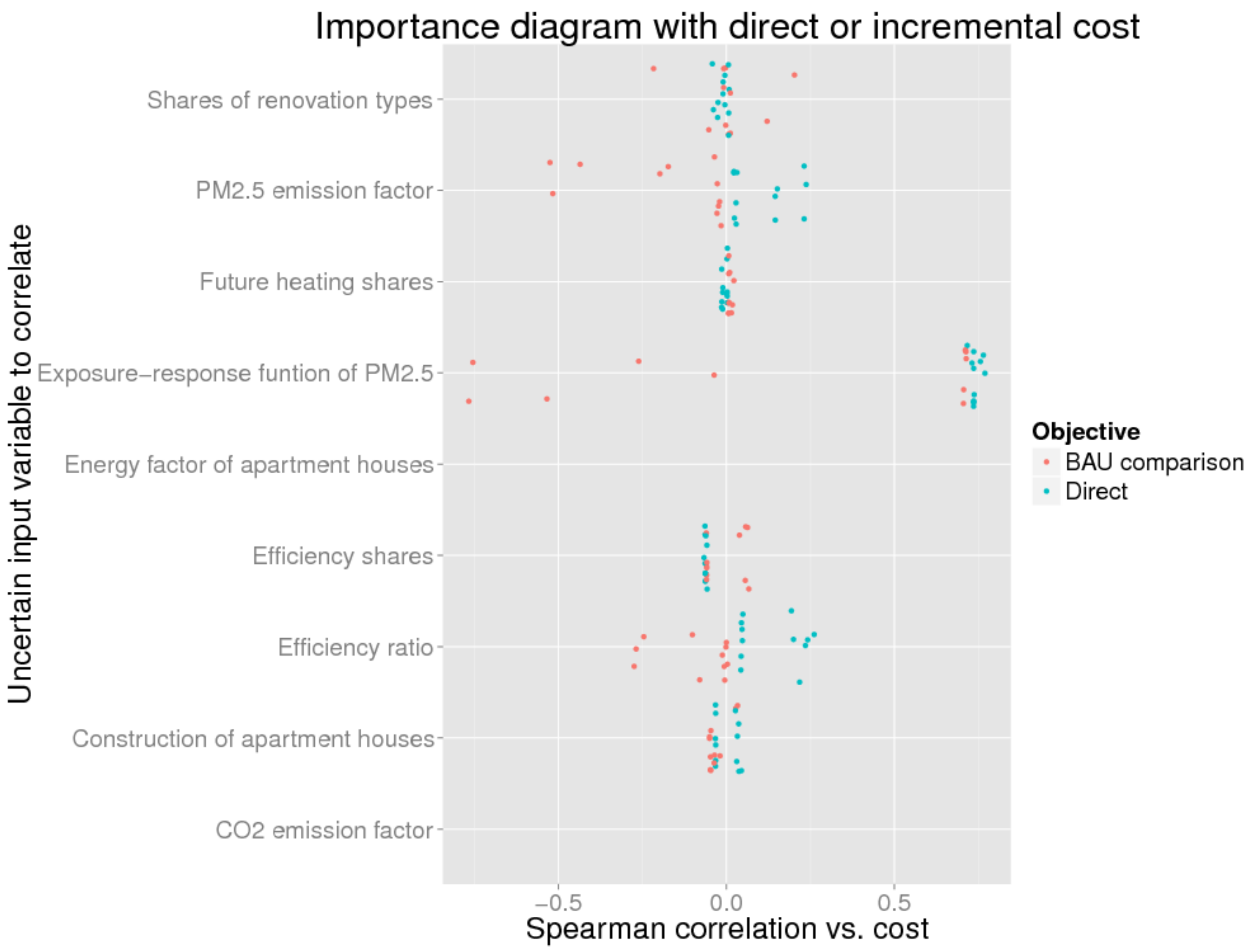
Importance diagram with direct or incremental cost
Uncertain input variable to correlate
Shares of renovation types
PM2.5 emission factor
Future heating shares
Exposure−response funtion of PM2.5
Energy factor of apartment houses
Efficiency shares
Efficiency ratio
Construction of apartment houses
CO2 emission factor
−0.5
0.0
0.5
Spearman correlation vs. cost
Objective
BAU comparison
Direct
